# Synthesis and Late-Stage Modification of (−)-Doliculide Derivatives Using Matteson’s Homologation Approach

**DOI:** 10.3390/md22040165

**Published:** 2024-04-08

**Authors:** Markus Tost, Uli Kazmaier

**Affiliations:** Organic Chemistry, Saarland University, Campus Building C4.2, D-66123 Saarbruecken, Germany; markus.tost@uni-saarland.de

**Keywords:** actin binder, click chemistry, doliculide, Matteson homologation, SAR studies

## Abstract

(−)-Doliculide, a marine cyclodepsipeptide derived from the Japanese sea hare, *Dolabella auricularia*, exhibits potent cytotoxic properties, sparking interest in the field of synthetic chemistry. It is comprised of a peptide segment and a polyketide moiety, rendering it amenable to Matteson’s homologation methodology. This technique facilitates the diversification of the distinctive polyketide side chain, thereby permitting the introduction of functional groups in late stages for modifications of the derived compounds and studies on structure–activity relationships.

## 1. Introduction

**Figure 1:**
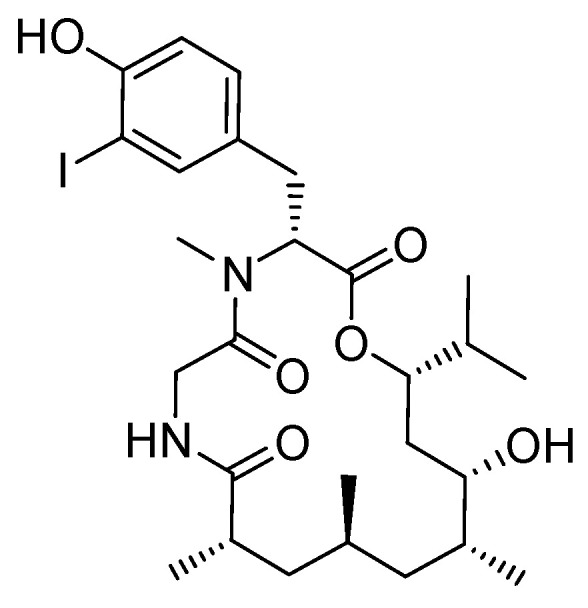
(−)-Doliculide.

Isolation of (−)-doliculide (Figure 1) from the Japanese sea hare, *Dolabella auricularia,* was first reported almost 30 years ago by Yamada et al., along with its potent cytotoxicity against HeLa-S_3_ cells (IC_50_ = 1 ng/mL) [1]. The mollusk itself may not necessarily be the producer of this cyclic depsipeptide, but metabolites isolated from *Dolabella auricularia* have been shown to originate from cyanobacteria and are therefore of dietary origin [2]. Due to their biological activities as anticancer agents, naturally derived cyclopeptides are interesting candidates for drug development, and so is doliculide [3,4,5].

Doliculide was found to act as a potent actin binder with a higher cell-membrane permeability than phalloidin and, thus, initiates actin aggregation, leading to inhibition of proliferation and apoptosis [6,7,8,9,10]. Subtoxic doses of doliculide lead to a transient change in reversible cytoskeleton dynamics and induction of premature senescence in p53 wild-type cells [11]. In a proof-of-concept study, doliculide was revealed to be a subtype-selective antagonist of the prostanoid E receptor 3 as a macromolecular target [12]. While the peptide and polyketide moieties of doliculide are both of some significance in actin binding, most synthetic derivatizations so far have focused on the peptide moiety, e. g., modifications in the (*R*)-Tyr-moiety, substitution of the iodo-(*R*)-Tyr part to Trp, or Gly to (*S*)-Ala [9,13,14].

Removal of the hydroxyl group within the polyketide moiety resulted in a notable sixfold reduction in cytotoxic activity against HeLa-S3 cells. Conversely, complete removal of the polyketide or all associated functionalities yielded derivatives that were inactive [13].

**Figure 2:**
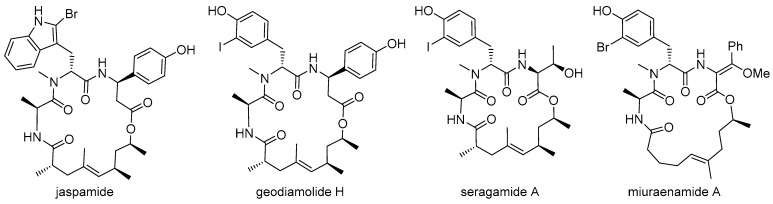
Structurally related F-Actin-stabilizing natural products.

It was shown that doliculide stabilizes F-actin in a similar way to structurally related natural products such as jaspamide [15], geodiamolide [16], seragamide [17], or miuraenamide [18,19,20] (Figure 2) [21]. Even the double-bound geometry in the miuraenamides has no significant effect on cytotoxicity [22]. All these cyclodepsipeptides are hybrids of a small peptide fragment and a variably substituted polyketide unit. Here, an α- or β-amino acid can be incorporated, which might be substituted or unsaturated. In the case of doliculide, this third amino acid is even missing, while the polyketide fragment is longer compared to most of the other natural products (except for the miuraenamides). This prompted our assumption that the isopropyl group at the terminus of the polyketide may be a replacement for the third amino acid, and that this may also be variable.

## 2. Results and Discussion

As our group is experienced in the syntheses of such peptide–polyketide conjugates [23,24,25,26], this assumption led us to develop a straightforward synthesis of doliculide, and associated derivatives, based on Matteson homologation [27,28,29], allowing late-stage variation at exactly this position [30]. In an initial modification, it was demonstrated that replacing the isopropyl group by the smaller methyl group led to almost no change in its cytotoxic activity. In principle, Matteson homologation should allow for the synthesis of a variety of derivatives simply by varying the nucleophiles in the homologation steps [30,31,32,33]. Therefore, we decided to also incorporate allyl and propargyl moieties via Matteson homologation, which could then be further used for late-stage modification—generating a variety of doliculide derivatives for structure–activity relationship (SAR) studies.

### 2.1. Preparation of Modified Polyketide Fragments

**Scheme 1:**
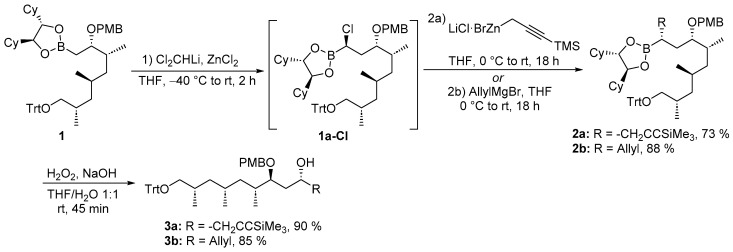
Syntheses of the polyketide fragments **3**.

Synthesis of the polyketide fragments started from the previously described boronic ester **1** [30], which was subjected to Matteson homologation with Cl_2_CHLi to obtain homologated α-chloroboronic ester, **1a-Cl** (Scheme 1). To facilitate preparation of **2a**, compound **1a-Cl** was quickly worked up before it was reacted with a Knochel-type propargylic zinc reagent [34,35], which gave better yields than the corresponding Grignard reagent. Furthermore, **1a-Cl** was reacted with AllylMgBr in a one-pot fashion to deliver **2b**. Both boronic esters, **2a** and **2b**, were subsequently oxidized to the corresponding polyketide fragments, **3a** and **3b**.

### 2.2. Syntheses of the Doliculide Core Structure

**Scheme 2:**
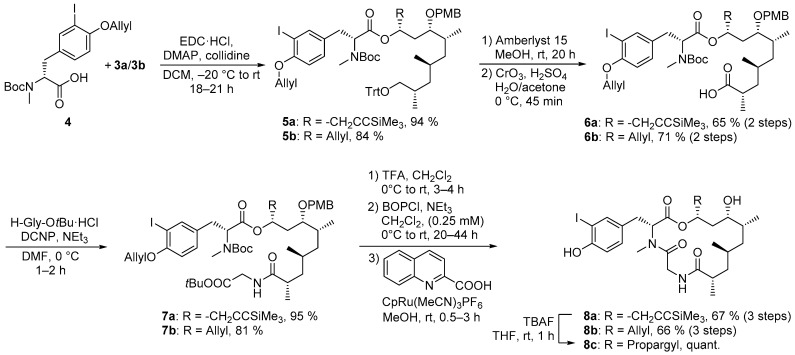
Finalization of the precursors **8** for late-stage modifications.

Steglich esterification of the alcohols, **3**, with modified (*R*)-Tyr, **4**, delivered the esters, **5**. Subsequent trityl deprotection and Jones oxidation generated acids, **6**, in acceptable yield even in the presence of the acid-labile Boc- and PMB-protecting groups (Scheme 2). The acids were then coupled with glycine *tert*-butyl ester to produce the peptides, **7**. After acidic cleavage of the Boc-, PMB- and *tert*-butyl-protection groups, cyclization using BOPCl (bis(2-oxo-3-oxazolidinyl)phosphinic chloride) as activator provided the corresponding cyclic peptides [36]. Unexpectedly, acidic treatment did not result in cleavage of the TMS-alkyne. Finally, CpRu(MeCN)_3_PF_6_-mediated removal of the allyl protecting group gave rise to both the TMS-protected alkyne, **8a**, and the allyl derivative, **8b** [22,37,38]. TMS deprotection of **8a** was achieved by treatment with TBAF and **8c** was obtained quantitatively.

### 2.3. Modification of the Doliculide Core Structure

**Scheme 3:**
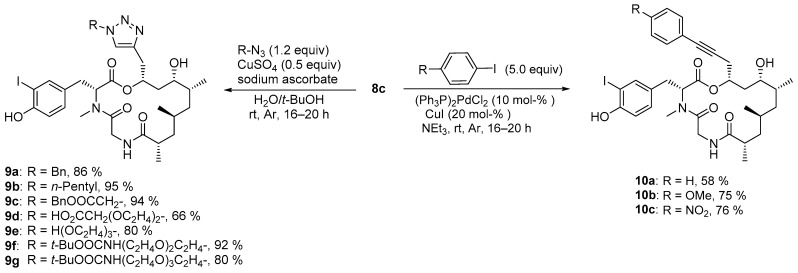
Modifications of doliculide derivative **8c**.

Precursor **8c** was then subjected to Cu(I)-catalyzed azide-alkyne cycloaddition (CuAAC) with various azides, yielding triazoles, **9**, in generally good yields (Scheme 3) [39,40]. Only the significantly more polar acid, **9d**, was obtained in somewhat lower yield.

In addition, alkyne **8c** was also used for Sonogashira couplings with several aryl iodides [41]. An excess of aryl iodide was used to prevent side-reactions which could occur with the electron-rich aryl iodide of the tyrosine moiety of **8c**. Good yields of **10** were obtained with both electron-rich and electron-poor iodoarenes.

**Scheme 4:**
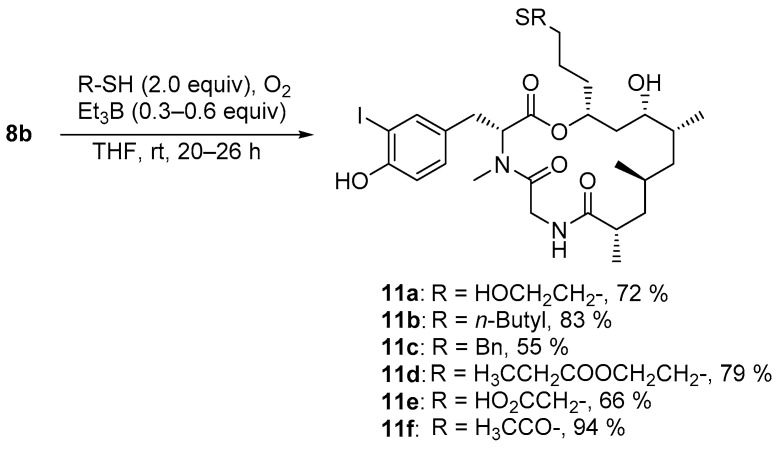
Thiol-ene click reactions with precursor **8b**.

Allyl derivative **8b** was subjected to a series of BEt_3_/O_2_-mediated radical thiol-ene reactions, giving rise to several functionalized thioethers, **11** (Scheme 4) [42,43]. Thioacetic acid could also be implemented, leading to the thioester **11f** in high yield. Only with ester and acid functionalities in the thiol component, the conversion was observed to be incomplete and, therefore, another 0.3 equiv. BEt_3_ were subsequently added, leading to full conversion.

## 3. Cytotoxicity Evaluation of New Doliculide Derivatives

Finally, with the doliculide derivates now available, we undertook SAR studies to determine the impact of the modifications at the end of the polyketide chain. The cytotoxicity of the derivatives **8**–**11** was investigated towards five different cancer cell lines. The IC_50_ values are given in Table 1 and compared to synthetic doliculide (**A**, entry 1).

Of all derivatives tested, only doliculide showed a high toxicity against most cancer cell lines. It was the only active compound against HepG2 (human hepatocellular carcinoma), and only propargyl-derivative **8c** showed any significant activity, although tenfold lower (entry 4). All other derivatives were almost inactive. Clearly, this cell line is extremely sensitive towards modification at this position. This derivative also showed good activity against U-2 OS (human bone osteosarcoma). Two triazole derivatives, **9a** and **9b**, were found to be the most active compounds against CHO-K1 (mutagenized Chinese hamster ovary carcinoma) (entries 5 and 6). Against KB3.1 (human epidermoid carcinoma cell line), they were comparably active with A or alkyne 10a. The thioethers **11b** and **11c** were found to be the most active compounds in this series (entries 16 and 17). The human colon carcinoma cell line (HCT-116) was found to be by far the most sensitive cell line. IC_50_ values in the low nM range, and comparable to A, were obtained with several doliculide derivatives such as **8a**, **8b**, **10a**, **10c** or **11c** (entries 2, 3, 12, 14 and 17).

## 4. Materials and Methods

The General Synthetic Methods were as follows: All air- or moisture-sensitive reactions were carried out in dried glassware (>100 °C) under an atmosphere of nitrogen or argon. Anhydrous solvents were purchased from Acros Organics (now Thermo Fisher Scientific, Waltham, MA, USA) or dried before use (THF was distilled over Na/benzophenone, diisopropylamine over CaH_2_, MeOH distilled with Magnesium and degassed via freeze–pump–thaw). ZnCl_2_ was fused in vacuo at 0.1 mbar prior to use. Ethyl acetate (EtOAc), petroleum ether and n-pentane were additionally distilled before use. Reactions that required cooling were cooled using conventional methods (ice/water for 0 °C, dry ice/acetone for −40 °C or −78 °C). Reactions that required heating above rt were heated using an oil bath. Reactions were monitored by NMR or analytical TLC, which was performed on precoated silica gel on Macherey-Nagel (Dueren, NRW, Germany) TLC-PET foils (Polygram^®^ SIL G/UV_254_). Visualization was accomplished with UV-light (254 nm), KMnO4 solution or Ce(IV)/ammonium molybdate solution. The products were purified by flash chromatography on Macherey-Nagel 60 silica gel columns (0.063–0.2 mm or 0.04–0.063 mm) or by automated flash chromatography on Büchi (Flawil, Switzerland) Pure C-815 Chromatography System and prepacked Teledyne Isco (Thousand Oaks, CA, USA) silica gel cartridges (RediSep Rf normal- Macherey-Nagelphase silica flash 30–70 µm columns). Reversed-phase flash chromatography was accomplished by automated flash chromatography on Büchi Reveleris Prep Chromatography System and Büchi FlashPure Select C18 30 µm spherical cartridges or Cole-Parmer Telos (Vernon Hills, IL, USA) C18 cartridges. Preparative HPLC was performed on a Büchi Reveleris Prep Chromatography System using a Phenomenex (Danaher Corporation, Washington, DC, USA) Luna C18(2) 100 Å column (250 × 21.1 mm, 5 µm). ^1^H- and ^13^C-NMR spectra were recorded with a Bruker (Billerica, MA, USA) AV II 400 [400 MHz (1H), 100 MHz (13C)], a Bruker AV 500 [500 MHz, (^1^H), 125 MHz (^13^C)] or a Bruker Avance Neo 500 [500 MHz, (^1^H), 125 MHz (^13^C)] spectrometer in CDCl_3_ or DMSO-D_6_. NMR spectra were evaluated using NMR Processor Version 12.01 from Advanced Chemistry Development Inc. (ACD/Labs, Toronto, ON, Canada) or Bruker TopSpin Version 4.1.1. Chemical shifts are reported in ppm relative to Si(CH_3_)_4_ and the solvent residual peak was used as the internal standard. Selected signals for the minor diastereomers/rotamers are extracted from the spectra of the isomeric mixture. Multiplicities are reported as bs (broad signal), s (singlet), d (doublet), t (triplet), q (quartet) and m (multiplet). Signals marked with * in ^13^C-NMR give broad signals. Structural assignments were made with additional information from gCOSY, gNOESY, gHSQC, or gHMBC experiments. Melting points were determined with a melting-point apparatus MEL-TEMP II by Laboratory Devices (Auburn, CA, USA) and are uncorrected. High-resolution mass spectra (HRMS) were recorded with a Finnigan (now Thermo Fisher Scientific) MAT95 spectrometer using the CI technique (CI) or a Bruker Daltonics 4G hr-ToF (ESI-ToF) or a Bruker solariX using the ESI technique (ESI-FTICR). Other HRMS were measured on a Thermo Fisher Scientific Orbitrap Q exactive mass spectrometer, equipped with a heated ESI source and a Thermo Finnigan (Thermo Fisher Scientific) Ultimate3000 HPLC (ESI-Q exactive). Optical rotations were measured in CHCl_3_ with a Krüss (Hamburg, Germany) polarimeter P8000 T80 in thermostated (20 °C ± 1 °C) cuvettes and are given in 10^−1^degcm^2^g^-1^. The radiation source used was a sodium vapor lamp (λ = 589 nm). The concentrations are given in g/100 mL.

The general procedure for Cu(I)-catalyzed azide alkyne cycloadditions (CuAAC) was as follows: The alkyne (1.0 equiv) and azide (1.2 equiv) were dissolved in a 1:1 mixture of *t*-BuOH and H_2_O (0.05 M) in a 1.5 mL vial. Under a gentle stream of argon, sodium ascorbate (0.6 equiv, 1 M in H_2_O), and copper(II) sulfate (0.5 equiv, 1 M in H_2_O) were added, the vial sealed and the typically light-brownish suspension stirred overnight at rt (typically 16–20 h). The reaction mixture was concentrated and the residue purified by column chromatography.

The general procedure for Sonogashira cross-coupling of terminal alkyne was as follows: The alkyne (1.0 equiv), bis(triphenylphosphine)palladium(II) chloride (0.1 equiv), and copper(I) iodide (0.2 equiv) were added in a 1.5 mL vial and gently purged with argon for 5 min. Triethylamine (0.15 M) and the iodoarene (5.0 equiv) were added under a gentle stream of argon, the vial closed and the typically white suspension stirred overnight (typically 16–20 h) after which the reaction typically turned black. The suspension was diluted with MeCN, filtrated through a syringe filter (0.2 µm, PTFE), the solvent removed in vacuo, and the residue was subjected to chromatographic purification.

The general procedure for thiol-ene click reaction was as follows: The alkene (1.0 equiv) was gently purged with nitrogen and dissolved in THF abs. (0.1 M) in a 1.5 mL vial. Thiol (2.0 equiv) and Et_3_B (0.3 equiv, 1 M in hexane) were subsequently added, and the vial closed. Initiation of the reaction was performed by addition of air (0.4 mL) via a syringe and the reaction mixture stirred overnight at rt (typically 20–26 h). The solvent was removed and the crude product was purified by chromatography.

### 4.1. Synthesis of the Polyketide Fragments

#### 4.1.1. Synthesis of {(4R,6S,7R,9R,11S)-4-[(4S,5S)-4,5-Dicyclohexyl-1,3,2-dioxaborolan-2-yl]-6-[(4-methoxybenzyl)oxy]-7,9,11-trimethyl-12-(trityloxy)dodec-1-yn-1-yl}trimethylsilane (**2a**)

Nucleophile solution: Lithium chloride (746 mg, 17.6 mmol, 1.10 equiv) was fused under reduced pressure. Zinc (2.09 g, 32.0 mmol, 2.0 equiv, dust) was added and the mixture suspended in anhydrous THF (16.0 mL). 1,2-Dibromoethane (28.0 µL, 320 µmol, 0.02 equiv) was added and briefly heated to reflux. It was cooled to rt and trimethylsilyl chloride (102 µL, 800 µmol, 0.05 equiv) was added. The mixture was briefly heated to reflux again and cooled to rt. (3-bromoprop-1-yn-1-yl)trimethylsilane [34] (3.06 g, 16.0 mmol, 1.0 equiv) in anhydrous THF (16.0 mL) was added dropwise (exothermic) and stirred for 1 h dat rt. The stirrer was stopped, and the nucleophile solution decanted. Titration of the solution^1^ gave a concentration of 0.36 M (approximately 73% of theory). LDA-solution: Diisopropylamine (1.37 mL, 9.63 mmol, 1.35 equiv) was dissolved in anhydrous THF (3.43 mL, 2.81 M) and cooled to −40 °C (acetone/dry ice). *n*-Butyllithium (3.57 mL, 8.91 mmol, 1.25 equiv, 2.5 M in hexane) was added dropwise, stirred for 10 min at −40 °C, warmed to rt and further stirred for 20 min. Homologation: Boronic ester **1** [30] (5.60 g, 7.13 mmol, 1.0 equiv) and DCM (1.38 mL, 21.4 mmol, 3.0 equiv) were dissolved in anhydrous THF (9.98 mL, 1.4 mL/mmol, 0.71 M) and cooled to −40 °C (acetone/dry ice). The previously prepared LDA solution was slowly added at this temperature and stirred for further 10 min after complete addition. A solution of zinc chloride (3.89 g, 28.5 mmol, 4.0 equiv) in anhydrous THF (17.1 mL, 0.6 mL/mmol, 1.67 M) was added, the reaction mixture warmed to rt and stirred for 2 h. Isolation and substitution: The reaction mixture was worked up with saturated NH_4_Cl and *n*-pentane. The phases were separated, and the aqueous phase extracted twice with *n*-pentane. The combined organic extracts were dried over Na_2_SO_4_ and the solvent removed under reduced pressure. The resulting residue was taken up in anhydrous THF (28.5 mL, 0.25 M), cooled to 0 °C and the previously (freshly) prepared nucleophile solution (21.8 mL, 7.84 mmol, 1.1 equiv) was added dropwise. It was slowly warmed to rt and stirred for 18 h. The reaction mixture was worked up with saturated NH_4_Cl and *n*-pentane. The phases were separated, the aqueous phase extracted twice with *n*-pentane and dried over Na_2_SO_4_. The solvent removed under reduced pressure and the resulting residue was subjected to chromatographic purification (SiO_2_, petroleum ether:EtOAc 98:2–95:5). Boronic ester **2a** (4.67 g, 7.13 mmol, 73%) was obtained as colorless resin. R_f_ (**2a**) = 0.22 (silica, petroleum ether:EtOAc 95:5). ${\left[\alpha \right]}_{D}^{20}$ = −33.0 (c = 1.0, CHCl_3_). ^1^H-NMR (400 MHz, CDCl_3_): δ = 0.13 (s, 9 H), 0.81 (d, *J* = 6.9 Hz, 3 H), 0.83 (d, *J* = 6.6 Hz, 1 H), 0.88–1.12 (m, 9 H), 1.13–1.21 (m, 6 H), 1.21–1.30 (m, 4 H), 1.35 (m, 1 H), 1.51 (m, 1 H), 1.54–1.61 (m, 5 H), 1.67 (m, 2 H), 1.71–1.81 (m, 6 H), 1.85 (m, 1 H), 1.95 (m, 1 H), 2.37 (m, 2 H), 2.83 (dd, *J* = 8.7, 6.9 Hz, 1 H), 2.99 (dd, *J* = 8.7, 5.1 Hz, 1 H), 3.40 (m, 1 H), 3.78–2-82 (m, 5 H), 4.34 (d, *J* = 11.1 Hz, 1 H), 4.47 (d, *J* = 11.1 Hz, 1 H), 6.84 (d, *J* = 8.7 Hz, 2 H), 7.20–7.26 (m, 5 H), 6.33 (m, 6 H), 7.46 (m, 6 H) ppm. ^13^C-NMR (100 MHz, CDCl_3_): δ = 0.00, 14.2, 18.4, 19.2*, 21.1, 22.2, 25.6, 25.8, 26.2, 27.3, 27.3, 28.4, 30.0, 31.1, 31.5, 40.5, 41.3, 42.8, 55.0, 68.2, 81.1, 83.3, 84.5, 85.9, 107.3, 113.4, 126.5, 127.4, 128.5, 128.9, 131.2, 144.3, 158.7 ppm. HRMS (ESI-FTICR) calcd. for C_59_H_85_BNO_5_Si^+^ [M+NH_4_]^+^: 925.63209 found 925.63421.

#### 4.1.2. Synthesis of (4S,5S)-4,5-Dicyclohexyl-2-{(4R,6S,7R,9R,11S)-6-[(4-methoxybenzyl)oxy]-7,9,11-trimethyl-12-(trityloxy)dodec-1-en-4-yl}-1,3,2-dioxaborolane (**2b**)

LDA-solution: Diisopropylamine (800 µL, 5.61 mmol, 1.35 equiv) was dissolved in anhydrous THF (830 µL, 6.75 M) and cooled to −40 °C (acetone/dry ice). *n*-Butyllithium (3.24 mL, 5.19 mmol, 1.25 equiv, 1.6 M in hexane) was added dropwise, stirred for 10 min at −40 °C, warmed to rt and further stirred for 20 min. Homologation: Boronic ester **1** (3.26 g, 4.15 mmol, 1.0 equiv) and DCM (802 µL, 12.5 mmol, 3.0 equiv) were dissolved in anhydrous THF (5.81 mL, 1.4 mL/mmol, 0.71 M) and cooled to −40 °C (acetone/dry ice). The previously prepared LDA solution was slowly added at this temperature and stirred for further 10 min after complete addition. A solution of zinc chloride (2.26 g, 16.6 mmol, 4.0 equiv) in anhydrous THF (9.97 mL, 0.6 mL/mmol, 1.67 M) was added, the reaction mixture warmed to rt and stirred for 2 h. Substitution: The reaction mixture was cooled to 0 °C (ice/water) and allylmagnesium chloride (10.4 mL, 10.4 mmol, 2.5 equiv 1.0 M in diethyl ether) was slowly added. It was warmed to rt and stirred for 18 h. The reaction mixture was worked up with saturated NH_4_Cl and *n*-pentane; the phases were separated and the aqueous phase extracted twice with *n*-pentane. The combined organic extracts were dried over Na_2_SO_4_ and the solvent removed under reduced pressure. The resulting residue was chromatographed (SiO_2_, *n*-pentane:EtOAc 98:2–95:5) and boronic ester **2b** (3.09 g, 3.64 mmol, 88%) obtained as colorless resin. R_f_ (**2b**) = 0.13 (silica, *n*-pentane:EtOAc = 97:3). ${\left[\alpha \right]}_{D}^{20}$ = −35.2 (c = 1.0, CHCl_3_). ^1^H-NMR (400 MHz, CDCl_3_): δ = 0.74 (d, *J* = 6.7 Hz, 3 H), 0.76–0.81 (m, *J* = 6.5 Hz, 4 H), 0.83–1.04 (m, *J* = 6.6 Hz, 8 H), 1.06–1.23 (m, 9 H_b_), 1.32 (ddd, *J* = 13.1, 7.5, 6.0 Hz, 1 H), 1.37–1.58 (m, 6 H), 1.64 (m, 2 H), 1.67–1.84 (m, 7 H), 1.90 (m, 1 H), 2.14 (ddddd, *J* = 13.8, 6.8, 6.8, 1.6, 1.6 Hz, 1 H), 2.19 (ddddd, *J*= 13.8, 6.8, 6.8, 1.6, 1.6 Hz, 1 H), 2.79 (dd, *J* = 8.6, 6.8 Hz, 1 H), 2.95 (dd, *J* = 8.7, 5.0 Hz, 1 H), 3.27 (m, 1 H), 3.74 (m, 2 H), 3.76 (s, 3 H), 4.29 (d, *J* = 11.2 Hz, 1 H), 4.43 (d, *J* = 11.1 Hz, 1 H), 4.90 (ddt, *J* = 10.1, 1.6, 1.6 Hz, 1 H), 4.97 (ddt, *J* = 17.0, 1.6, 1.6 Hz, 1 H), 5.78 (dddd, *J* = 17.0, 10.1, 6.9, 6.9 Hz, 1 H), 6.81 (d, *J* = 8.6 Hz, 2 H), 7.16–7.22 (m, 5 H), 7.26 (m, 6 H), 7.43 (m, 6 H) ppm. ^13^C-NMR (100 MHz, CDCl_3_): δ = 14.1, 18.8, 19.3*, 21.4, 25.9, 26.0, 26.5, 27.7, 27.7, 28.6, 30.7, 31.4, 31.7, 36.7, 41.1, 41.4, 43.1, 55.2, 68.3, 70.8, 81.8, 83.5, 86.1, 113.6, 114.9, 126.8, 127.6, 128.7, 129.4, 131.3, 138.7, 144.5, 158.9 ppm. HRMS (ESI-Q exactive) calcd. for C_56_H_76_BO_5_^+^ [M+H]^+^: 838.5702 found 838.5651.

#### 4.1.3. Synthesis of (4R,6S,7R,9R,11S)-6-[(4-Methoxybenzyl)oxy]-7,9,11-trimethyl-1-(trimethylsilyl)-12-(trityloxy)dodec-1-yn-4-ol (**3a**)

Boronic ester **2a** (4.70 g, 5.18 mmol, 1.0 equiv) was dissolved in THF (10.3 mL, 0.5 M) and cooled to 0 °C. Hydrogen peroxide (2.40 mL, 25.8 mmol, 5.0 equiv, 33% in H_2_O) and sodium hydroxide (1.03 g, 25.8 mmol, 5.0 equiv) dissolved in H_2_O (10.3 mL, 2.5 M) were added. The reaction mixture was warmed to rt and stirred for 60 min. Saturated NaCl solution was added, and the mixture extracted thrice with diethyl ether. The combined organic extracts were dried over Na_2_SO_4_ and the solvent removed under reduced pressure. The residue was chromatographed (SiO_2_, petroleum ether:EtOAc 95:5–7:3) and **3a** (3.30 g, 4.63 mmol, 90%) obtained as colorless resin. In another fraction, (*S*,*S*)-Dicyclohexylethanediol (753 mg, 3.33 mmol, 64%) was obtained as colorless needles. R_f_ (**3a**) = 0.59 (silica, *n*-pentane:EtOAc 8:2). ${\left[\alpha \right]}_{D}^{20}$ = −19.0 (c = 1.0, CHCl_3_). ^1^H-NMR (400 MHz, CDCl_3_): δ = 0.15 (s, 9 H), 0.79 (d, *J* = 6.5 Hz, 1 H), 0.84 (d, *J* = 6.5 Hz, 3 H), 0.88–0.95 (m, 2 H), 1.00 (d, *J* = 6.5 Hz, 3 H), 1.23 (m, 1 H), 1.37 (m, 1 H), 1.45 (m, 1 H), 1.56 (m, 1 H), 1.67 (m, 1 H), 1.84 (m, 1 H), 1.99 (m, 1 H), 2.39 (m, 2 H), 2.66 (d, *J* = 4.2 Hz, 1 H), 2.82 (dd, *J* = 8.7, 6.6 Hz, 1 H), 2.99 (dd, *J* = 8.7, 5.0 Hz, 1 H), 3.55 (m, 1 H), 3.79 (s, 3 H), 3.95 (m, 1 H), 4.36 (d, *J* = 11.0 Hz, 1 H), 4.49 (d, *J* = 11.0 Hz, 1 H), 6.85 (d, *J* = 8.7 Hz, 2 H), 7.20–7.25 (m, 5 H), 7.29 (m, 6 H), 7.45 (m, 6 H) ppm. ^13^C-NMR (100 MHz, CDCl_3_): δ = 0.1, 14.4, 18.9, 21.2, 27.9, 28.8, 31.4, 31.5, 34.4, 41.3, 41.5, 55.3 (q, C-13), 67.4, 68.3, 70.8, 79.5, 86.2, 87.1, 103.6, 113.8, 126.8, 127.6, 128.8, 129.5, 130.6, 144.5, 159.2 ppm. HRMS (CI) calcd. for C_45_H_59_O_4_Si^+^ [M]^+^: 691.4177 found 691.4205.

#### 4.1.4. Synthesis of (4R,6S,7R,9R,11S)-6-[(4-Methoxybenzyl)oxy]-7,9,11-trimethyl-12-(trityloxy)dodec-1-en-4-ol (**3b**)

Boronic ester **2b** (3.00 g, 3.54 mmol, 1.0 equiv) was dissolved in THF (7.07 mL, 0.5 M) and cooled to 0 °C. Hydrogen peroxide (1.64 mL, 17.7 mmol, 5.0 equiv, 33% in H_2_O) and sodium hydroxide (707 mg, 17.7 mmol, 5.0 equiv) dissolved in H_2_O (7.07 mL, 2.5 M) were added. The reaction mixture was warmed to rt and stirred for 45 min. Saturated NaCl solution was added, and the mixture extracted thrice with diethyl ether. The combined organic extracts were dried over Na_2_SO_4_ and the solvent removed under reduced pressure. The residue was chromatographed (SiO_2_, *n*-pentane:EtOAc 9:1–8:2) and **3b** (1.87 g, 43.0 mmol, 85%) obtained as colorless resin. R_f_ (**3b**) = 0.50 (silica, *n*-pentane:EtOAc 8:2). ${\left[\alpha \right]}_{D}^{20}$ = −25.2 (c = 1.0, CHCl_3_). ^1^H-NMR (400 MHz, CDCl_3_): δ = 0.79 (d, *J* = 6.7 Hz, 3 H), 0.81–0.91 (m, *J* = 6.5 Hz, 5 H), 0.99 (d, *J* = 6.7 Hz, 3 H), 1.20 (ddd, *J* = 13.5, 6.5, 6.5 Hz, 1 H), 1.32–1.47 (m, 3 H), 1.59 (ddd, *J* = 14.4, 9.5, 2.3 Hz, 1 H), 1.82 (m, 1 H), 2.00 (m, 1 H), 2.19 (m, 2 H), 2.29 (d, *J* = 4.5 Hz, 1 H), 2.82 (dd, *J* = 8.7, 6.7 Hz, 1 H), 2.98 (dd, *J* = 8.7, 5.1 Hz, 1 H), 3.55 (ddd, *J* = 9.4, 4.6, 2.3 Hz, 1 H), 3.79 (s, 3 H), 3.85 (m, 1 H), 4.36 (d, *J* = 11.1 Hz, 1 H), 4.51 (d, *J* = 11.1 Hz, 1 H), 5.05–5.13 (m, 2 H), 5.80 (ddt, *J* = 17.4, 9.8, 7.2 Hz, 1 H), 6.86 (d, *J* = 8.7 Hz, 2 H), 7.18–7.25 (m, 5 H), 7.29 (m, 6 H), 7.45 (m, 6 H) ppm. ^13^C-NMR (100 MHz, CDCl_3_): δ = 14.5, 18.8, 21.1, 27.8, 31.3, 31.4, 35.0, 41.1, 41.6, 42.2, 55.3 (q, C-12), 67.9, 68.3, 70.4, 78.9, 86.1, 113.8, 117.6, 126.8, 127.6, 128.7, 129.5, 130.6, 135.1, 144.5, 159.2 ppm. HRMS (ESI-Q exactive) calcd. for C_42_H_52_O_4_K^+^ [M+K]^+^: 659.3497 found 659.3500.

### 4.2. Syntheses of the Doliculide Core Structure

#### 4.2.1. Synthesis of (4R,6S,7R,9R,11S)-6-[(4-Methoxybenzyl)oxy]-7,9,11-trimethyl-1-(trimethylsilyl)-12-(trityloxy)dodec-1-yn-4-yl (R)-3-[4-(allyloxy)-3-iodophenyl]-2-[(tert-butoxycarbonyl)(methyl)amino]propanoate (**5a**)

Carboxylic acid **4**[30] (4.23 g, 9.18 mmol, 2.0 equiv) and alcohol **3a** (3.27 g, 4.59 mmol, 1.0 equiv) were dissolved in anhydrous DCM (92.0 mL, 0.05 M) and cooled to −20 °C. DMAP (196 mg, 1.61 mmol, 0.35 equiv), EDC·HCl (1.76 g, 9.18 mmol, 2.0 equiv) and collidine (1.22 mL, 9.18 mmol, 2.0 equiv) were added and the reaction mixture stirred at -20 °C (acetone/cryostat) for 21 h. The reaction mixture was worked up with EtOAc and 1 M KHSO_4_. The organic phase was washed with saturated NaHCO_3_ and brine. The organic extract was dried over Na_2_SO_4_ and the solvent removed under reduced pressure. The residue was chromatographed (SiO_2_, petroleum ether:EtOAc 9:1–8:2) and ester **5a** (4.88 g, 4.30 mmol, 94%) obtained as colorless resin. R_f_ (**5a**) = 0.44 (silica, petroleum ether: EtOAc 8:2). ${\left[\alpha \right]}_{D}^{20}$ = −13.1 (c = 1.0, CHCl_3_). ^1^H-NMR (500 MHz, 373 K, DMSO-D_6_): δ = 0.13 (s, 9 H), 0.79 (d, *J* = 6.6 Hz, 3 H), 0.82 (d, *J* = 6.6 Hz, 3 H), 0.87–0.92 (m, 2 H), 1.18 (m, 1 H), 1.32 (s, 9 H), 1.37 (m, 1 H), 1.44 (m, 1 H), 1.70–1.78 (m, 3 H), 1.93 (m, 1 H), 2.56 (m, 2 H), 2.68 (s, 3 H), 2.85 (dd, *J* = 8.9, 6.6 Hz, 1 H), 2.91 (m, 1 H), 2.98 (dd, *J* = 8.9, 5.0 Hz, 1 H), 3.10 (dd, *J* = 14.4, 5.0 Hz, 1 H), 3.30 (m, 1 H), 3.75 (s, 3 H), 4.24 (d, *J* = 11.0 Hz, 1 H), 4.40 (d, *J* = 11.0 Hz, 1 H), 4.57 (m, 2 H), 4.69 (dd, *J* = 10.4, 5.0 Hz, 1 H), 5.08 (m, 1 H), 5.25 (dd, *J* = 1.6, 10.7 Hz, 1 H), 5.46 (dd, *J* = 1.6, 17.3 Hz, 1 H), 6.03 (ddt, *J* = 17.3, 10.7, 5.0 Hz, 1 H), 6.85–6.90 (m, 3 H), 7.14 (dd, *J* = 8.2, 2.2 Hz, 1 H), 7.17–7.26 (m, 5 H), 7.30 (m, 6 H), 7.39 (m, 6 H), 7.61 (d, *J* = 2.2 Hz, 1 H) ppm. ^13^C-NMR (125 MHz, 373 K, DMSO-D_6_): δ = −0.6, 13.8, 17.9, 20.5, 24.8, 27.5, 27.5, 30.7, 31.2, 31.2, 32.6, 32.7, 40.1, 40.8, 54.7, 59.6, 67.7, 69.1, 69.5, 69.8, 77.4, 78.7, 85.5, 86.0, 86.5, 102.4, 112.7, 113.3, 116.5, 126.3, 127.1, 127.8, 128.4, 129.5, 130.5, 131.7, 132.7, 138.7, 143.7, 154.0, 155.2, 158.4, 169.2 ppm. HRMS (ESI-ToF) calcd. for C_44_H_67_INO_8_Si^+^ [M–Trt+H]^+^: 892.3675 found 892.3674.

#### 4.2.2. Synthesis of (4R,6S,7R,9R,11S)-6-[(4-Methoxybenzyl)oxy]-7,9,11-trimethyl-12-(trityloxy)dodec-1-en-4-yl (R)-3-[4-(allyloxy)-3-iodophenyl]-2-[(tert-butoxycarbonyl)(methyl)amino]propanoate (**5b**)

Carboxylic acid **4** (2.49 g, 5.39 mmol, 2.0 equiv) and alcohol **3b** (1.67 g, 2.69 mmol, 1.0 equiv) were dissolved in anhydrous DCM (53.9 mL, 0.05 M) and cooled to -20 °C. DMAP (115 mg, 943 µmol, 0.35 equiv), EDC·HCl (1.03 g, 5.39 mmol, 2.0 equiv) and collidine (718 µL, 5.39 mmol, 2.0 equiv) were added and the reaction mixture stirred at -20 °C (acetone/cryostat) for 18 h. The reaction mixture was worked up with EtOAc and 1 M KHSO_4_. The organic phase was washed with saturated NaHCO_3_ and brine. The organic extract was dried over Na_2_SO_4_ and the solvent removed under reduced pressure. The residue was chromatographed (SiO_2_, petroleum ether:EtOAc 9:1–8:2) and ester **5b** (2.41 g, 2.27 mmol, 84%) obtained as colorless resin. R_f_ (**5b**) = 0.35 (silica, *n*-pentane:EtOAc 8:2). ${\left[\alpha \right]}_{D}^{20}$ = –8.0 (c = 1.0, CHCl_3_). ^1^H-NMR (500 MHz, 373 K, DMSO-D_6_): δ = 0.77 (d, *J* = 6.7 Hz, 3 H), 0.82 (d, *J* = 6.4 Hz, 3 H), 0.83–0.91 (m, 2 H), 0.93 (d, 3 H), 1.16 (m, 1 H), 1.30–1.36 (m, 10 H), 1.42 (m, 1 H), 1.58 (m, 2 H), 1.73 (m, 1 H), 1.91 (m, 1 H), 2.33 (m, 2 H), 2.65 (s, 3 H), 2.85 (dd, *J* = 9.0, 6.4 Hz, 1 H), 2.90 (dd, *J* = 14.3, 10.0 Hz, 1 H), 2.97 (dd, *J* = 9.0, 5.3 Hz, 1 H), 3.08 (dd, *J* = 14.3, 5.3 Hz, 1 H), 3.27 (ddd, *J* = 8.1, 8.1, 3.8 Hz, 1 H), 3.75 (s, 3 H), 4.24 (d, *J* = 11.1 Hz, 1H), 4.38 (d, *J* = 11.1 Hz, 1 H), 4.57 (ddd, *J* = 5.0, 1.6, 1.8 Hz, 2 H), 4.65 (dd, *J* = 10.0, 5.3 Hz, 1 H), 5.02–5.13 (m, 3 H), 5.25 (ddt, *J* = 10.4, 1.6, 1.6 Hz, 1 H), 5.45 (ddt, *J* = 17.2, 1.8, 1.6 Hz, 1 H), 5.73 (ddt, *J* = 17.1, 10.2, 7.0 Hz, 1 H), 6.02 (ddt, *J* = 17.3, 10.4, 5.1 Hz, 1 H), 6.84–6.90 (m, 3 H), 7.14 (dd, *J* = 8.4, 2.1 Hz, 1 H), 7.19 (d, *J* = 8.5 Hz, 2 H), 7.23 (tt, *J* = 7.3, 1.2 Hz, 3 H), 7.30 (m, 6 H), 7.40 (m, 6 H), 7.80 (d, *J* = 2.0 Hz, 1 H) ppm. ^13^C-NMR (125 MHz, 373 K, DMSO-D_6_): δ = 13.8, 17.9, 20.5, 27.5, 27.5, 30.7, 31.1, 31.1, 32.6, 33.2, 38.1, 40.0, 40.8, 54.7, 59.6, 67.8, 69.1, 69.9, 71.2, 77.7, 78.7, 85.5, 86.0, 112.7, 113.3, 116.6, 117.0, 126.3, 127.1, 127.8, 128.4, 129.5, 130.5, 131.8, 132.7, 133.0, 138.6, 143.7, 154.0, 155.2, 158.4, 169.3 ppm. HRMS (ESI-Q exactive) calcd. for C_60_H_74_INO_8_Na^+^ [M+Na]^+^: 1086.4351 found 1086.4358.

#### 4.2.3. Synthesis of (4R,6S,7R,9R,11S)-12-Hydroxy-6-[(4-methoxybenzyl)oxy]-7,9,11-trimethyl-1-(trimethylsilyl)dodec-1-yn-4-yl (R)-3-[4-(allyloxy)-3-iodophenyl]-2-[(tert-butoxycarbonyl)(methyl)amino]propanoate (**5a-1**)

Trityl ether **5a** (2.52 g, 2.22 mmol, 1.0 equiv) was dissolved in MeOH (44.4 mL, 0.05 M). Amberlyst 15 (2.52 g, 100 m%) was added at rt and gently stirred for 20 h. EtOAc was added and the solid Amberlyst 15 was removed by filtration and stirred in ethyl acetate for 1 h. Amberlyst was again removed by filtration and both organic phases combined and washed with H_2_O. The aqueous phase was extracted with ethyl acetate and the combined organic extracts were dried over Na_2_SO_4_. After removal of the solvent under reduced pressure, the residue was subjected to chromatographic purification (silica, *n*-pentane:EtOAc 7:3) and alcohol **5a-1** (1.69 g, 1.90 mmol, 85%) obtained as colorless resin. R_f_ (**5a-1**) = 0.33 (silica, *n*-pentane:EtOAc 7:3). ${\left[\alpha \right]}_{D}^{20}$ = −16.6 (c = 0.5, CHCl_3_). ^1^H-NMR (500 MHz, 373 K, DMSO-D_6_): δ = 0.14 (s, 9 H), 0.83 (ddd, *J* = 13.8, 7.2, 7.2 Hz, 1 H), 0.85 (d, *J* = 6.6 Hz, 3 H), 0.86 (d, *J* = 6.9 Hz, 3 H), 0.89 (d, *J* = 6.6 Hz, 3 H), 0.94 (ddd, *J* = 13.8, 7.2, 7.2 Hz, 1 H), 1.23 (ddd, *J* = 13.8, 6.4, 6.4 Hz, 1 H), 1.30–1.37 (m, 10 H), 1.53–1.63 (m, 2 H), 1.71–1.77 (m, 2 H), 2.02 (m, 1 H), 2.54–2.60 (m, 2 H), 2.68 (s, 3 H), 2.91 (dd, *J* = 14.4, 10.4 Hz, 1 H), 3.11 (dd, *J* = 14.4, 5.0 Hz, 1 H), 3.16 (dd, *J* = 10.4, 6.6 Hz, 1 H), 3.30 (dd, *J* = 10.2, 5.2 Hz, 1 H), 3.33 (m, 1 H), 3.76 (s, 3 H), 4.27 (d, *J* = 11.3 Hz, 1 H), 4.44 (d, *J* = 11.3 Hz, 1 H), 4.58 (dt, *J* = 5.0, 1.6, 1.6 Hz, 2 H), 4.70 (dd, *J* = 10.4, 5.0 Hz, 1 H), 5.09 (m, 1 H), 5.26 (ddt, *J* = 10.6, 1.6, 1.6 Hz, 1 H), 5.46 (ddt, *J* = 17.3, 1.8, 1.8 Hz, 1 H), 6.03 (ddt, *J* = 17.3, 10.6, 4.9 Hz, 1 H), 6.87–6.92 (m, 3 H), 7.16 (dd, *J* = 8.5 Hz, *J* = 2.2 Hz, 1 H), 7.23 (m, 2 H), 7.61 (d, *J* = 2.2 Hz, 1 H) ppm. ^13^C-NMR (125 MHz, 373 K, DMSO-D6): δ = −0.5, 13.9, 17.4, 20.6, 24.9, 27.3, 27.5, 31.0, 31.0, 32.6, 32.6, 32.6, 40.2, 40.7, 54.6, 59.6, 66.0, 69.1, 59.6, 69.8, 77.2, 78.8, 86.0, 86.5, 102.5, 112.7, 113.3, 116.6, 128.5, 129.6, 130.5, 131.7, 132.8, 138.7, 155.2, 155.2, 158.4, 169.3 ppm. HRMS (ESI-ToF) calcd. for C_44_H_67_INO_8_Si^+^ [M+H]^+^: 892.3675 found 892.3660.

#### 4.2.4. Synthesis of (4R,6S,7R,9R,11S)-12-Hydroxy-6-[(4-methoxybenzyl)oxy]-7,9,11-trimethyldodec-1-en-4-yl (R)-3-[4-(allyloxy)-3-iodophenyl]-2-[(tert-butoxycarbonyl)(methyl)amino]propanoate (**5b-1**)

Trityl ether **5b** (2.32 g, 2.18 mmol, 1.0 equiv) was dissolved in MeOH (43.7 mL, 0.05 M). Amberlyst 15 (2.32 g, 100 m%) was added at rt and gently stirred for 20 h. EtOAc was added and the solid Amberlyst 15 was removed by filtration and stirred in ethyl acetate for 1 h. Amberlyst was again removed by filtration and both organic phases combined and washed with H_2_O. The aqueous phase was extracted with ethyl acetate and the combined organic extracts were dried over Na_2_SO_4_. After removal of the solvent under reduced pressure, the residue was subjected to chromatographic purification (silica, *n*-pentane:EtOAc 7:3) and alcohol **5b-1** (1.61 g, 1.94 mmol, 89%) obtained as colorless resin. R_f_ (**5b-1**) = 0.33 (silica, *n*-pentane:EtOAc 7:3). ${\left[\alpha \right]}_{D}^{20}$ = −11.6 (c = 1.0, CHCl_3_). ^1^H-NMR (500 MHz, 373 K, DMSO-D_6_): δ = 0.80–0.85 (m, *J* = 7.0 Hz, 4 H), 0.86 (d, *J* = 6.7 Hz, 3 H), 0.89 (d, *J* = 6.6 Hz, 3 H), 0.94 (m, 1 H), 1.23 (m, 1 H), 1.31–1-38 (m, 10 H), 1.52–1.64 (m, 4 H), 1.99 (m, 1 H), 2.35 (m, 2 H), 2.66 (s, 3 H), 2.92 (dd, *J* = 14.3, 10.3 Hz, 1 H), 3.10 (dd, *J* = 14.3, 5.3 Hz, 1 H), 3.17 (dd, *J* = 10.2, 6.6 Hz, 1 H), 3.30 (m, *J* = 10.2, 5.2 Hz, 2 H), 3.77 (s, 3 H), 4.27 (d, *J* = 11.1 Hz, 1 H), 2.42 (d, *J* = 11.1 Hz, 1 H), 4.58 (ddd, *J* = 4.9, 1.7, 1.5 Hz, 2 H), 4.67 (dd, *J* = 10.3, 5.3 Hz, 1 H), 5.02–5.13 (m, 3 H), 5.26 (ddt, *J* = 10.7, 1.5, 1.5 Hz, 1 H), 5.45 (ddt, *J* = 17.3, 1.7, 1.5 Hz, 1 H), 5.75 (ddt, *J* = 17.1, 10.2, 7.0 Hz, 1 H), 6.03 (ddt, *J* = 17.2, 10.5, 5.1 Hz, 1 H), 6.87–6.93 (m, 3 H), 7.17 (dd, *J* = 8.4, 2.0 Hz, 1 H), 7.23 (d, *J* = 8.5 Hz, 2 H), 7.62 (d, *J* = 8.5 Hz, 1 H) ppm. ^13^C-NMR (125 MHz, 373 K, DMSO-D_6_): δ = 13.9, 17.2, 20.5, 27.3, 27.5, 31.0, 32.0, 32.5, 32.6, 33.0, 38.1, 40.1, 40.7, 54.7, 59.7, 66.0, 69.1, 69.9, 71.2, 77.4, 78.8, 86.0, 112.7, 113.3, 116.6, 117.0, 128.5, 129.5, 130.6, 131.8, 132.8, 133.0, 138.7, 154.1, 155.2, 158.4, 169.3 ppm. HRMS (ESI-ToF) calcd. for C_41_H_61_INO_8_^+^ [M+H]^+^: 822.3436 found 822.3410.

#### 4.2.5. Synthesis of (2S,4S,6R,7S,9R)-9-({(R)-3-[4-(Allyloxy)-3-iodophenyl]-2-[(tert-butoxycarbonyl)(methyl)amino]propanoyl}oxy)-7-[(4-methoxybenzyl)oxy]-2,4,6-trimethyl-12-(trimethylsilyl)dodec-11-ynoic Acid (**6a**)

Alcohol **5a-1** (3.18 g, 3.57 mmol, 1.0 equiv) was dissolved in acetone (35.7 mL, 0.1 M) and cooled to 0 °C. Jones reagent (2.97 mL, 8.91 mmol, 3 M in 16% H_2_SO_4_, 2.5 equiv) were added and the mixture stirred for 45 min at this temperature. The reaction mixture was quenched with *i*-PrOH and concentrated under reduced pressure. The residue was diluted with H_2_O and EtOAc, the phases were separated and the aqueous phase extracted twice with EtOAc. The combined organic extracts were washed with brine, dried over Na_2_SO_4_. After removal of the solvent under reduced pressure, the residue chromatographically purified (silica, *n*-pentane:EtOAc 9:1–7:3) and acid **6a** (2.47 g, 2.72 mmol, 76%) obtained as colorless resin. R_f_ (**6a**) = 0.33 (silica, *n*-pentane:EtOAc 7:3). ${\left[\alpha \right]}_{D}^{20}$ = 0.6 (c = 0.5, CHCl_3_). ^1^H-NMR (500 MHz, 373 K, DMSO-D_6_): δ = 0.14 (s, 9 H), 0.85 (d, *J* = 6.7 Hz, 3 H), 0.90 (d, *J* = 6.6 Hz, 3 H), 0.95–1.05 (m, 2 H), 1.08 (d, *J* = 7.0 Hz, 3 H), 1.20 (ddd, *J* = 13.4, 8.0, 5.0 Hz, 1 H), 1.33 (s, 9 H), 1.54 (m, 1 H), 1.70 (ddd, *J* = 13.5, 9.0, 4.7 Hz, 1 H), 1.75 (m, 2 H), 1.98 (m, 1 H), 2.41 (ddq, *J* = 9.0, 7.1, 4.8 Hz, 1 H), 2.57 (m, 2 H), 2.69 (s, 3 H), 2.92 (dd, *J* = 14.4, 10.8 Hz, 1 H), 3.12 (dd, *J* = 14.4, 5.2 Hz, 1 H), 3.30 (ddd, *J* = 8.0, 4.0, 4.0 Hz, 1 H), 3.77 (s, 3 H), 4.29 (d, *J* = 11.1 Hz, 1 H), 4.44 (d, *J* = 11.1 Hz, 1 H), 4.59 (ddd, *J* = 5.0, 1.7, 1.5 Hz, 2 H), 4.71 (dd, *J* = 10.5, 5.2 Hz, 1 H), 5.08 (m, 1 H), 5.26 (ddt, *J* = 10.7, 1.5, 1.5 Hz, 1 H), 5.45 (ddt, *J* = 17.3, 1.7, 1.5 Hz, 1 H), 6.03 (ddt, *J* = 17.2, 10.5, 5.0 Hz, 1 H), 6.86–6.93 (m, 3 H), 7.16 (dd, *J* = 8.4, 2.0 Hz, 1 H), 7.23 (d, *J* = 8.5 Hz, 2 H), 7.62 (d, *J* = 2.0 Hz, 1 H). ^13^C-NMR (125 MHz, 373 K, DMSO-D6): δ = –0.46, 13.7, 17.6, 20.1, 25.0, 27.6, 28.1, 31.4, 31.4, 32.8, 33.0, 36.5, 40.2, 40.4, 54.9, 59.7, 69.3, 69.8, 70.0, 77.8, 79.0, 86.1, 86.7, 102.6, 112.9, 113.5, 116.8, 128.6, 129.7, 130.6, 131.9, 132.9, 138.8, 154.4, 155.4, 158.6, 169.5, 176.9. HRMS (ESI-ToF) calcd. for C_44_H_65_INO_9_Si^+^ [M+H]^+^: 906.3468 found 906.3487.

#### 4.2.6. Synthesis of (2S,4S,6R,7S,9R)-9-({(R)-3-[4-(Allyloxy)-3-iodophenyl]-2-[(tert-butoxycarbonyl)(methyl)amino]propanoyl}oxy)-7-[(4-methoxybenzyl)oxy]-2,4,6-trimethyldodec-11-enoic Acid (**6b**)

Alcohol **5b-1** (1.51 g, 1.82 mmol, 1.0 equiv) was dissolved in acetone (18.2 mL, 0.1 M) and cooled to 0 °C. Jones reagent (1.51 mL, 4.54 mmol, 3 M in 16% H_2_SO_4_, 2.5 equiv) were added and the mixture stirred for 45 min at this temperature. The reaction mixture was quenched with *i*-PrOH and concentrated under reduced pressure. The residue was diluted with H_2_O and EtOAc, the phases were separated, and the aqueous phase was extracted twice with EtOAc. The combined organic extracts were washed with brine, dried over Na_2_SO_4_. After removal of the solvent under reduced pressure, the residue chromatographically purified (silica, *n*-pentane:EtOAc 9:1–7:3) and acid **6b** (1.23 g, 1.46 mmol, 80%) obtained as colorless resin. R_f_ (**6b**) = 0.27 (silica, *n*-pentane:EtOAc 8:2). ${\left[\alpha \right]}_{D}^{20}$ = –4.2 (c = 1.0, CHCl_3_). ^1^H-NMR (500 MHz, 373 K, DMSO-D_6_): δ = 0.83 (d, *J* = 6.9 Hz, 3 H), 0.90 (d, *J* = 6.6 Hz, 3 H), 0.97 (ddd, *J* = 13.5, 8.4, 6.1 Hz, 1 H), 1.02 (ddd, *J* = 13.7, 8.4, 5.3 Hz, 1 H), 1.08 (d, *J* = 6.9 Hz, 3 H), 1.19 (ddd, *J* = 13.5, 7.7, 5.3 Hz, 1 H), 1.35 (s, 9 H), 1.52 (m, 1 H), 1.61 (m, 2 H), 1.68 (ddd, *J* = 13.7, 8.9, 4.8 Hz, 1 H), 1.97 (m, 1 H), 2.28–2.38 ( (m, 2 H), 2.42 (ddq, *J* = 8.9, 6.9, 5.3 Hz, 1 H), 2.66 (s, 9 H), 2.92 (dd, *J* = 14.5, 10.1 Hz, 1 H), 3.11 (dd, *J* = 14.5, 5.5 Hz, 1 H), 3.28 (m, 1 H), 3.77 (s, 3 H), 4.27 (d, *J* = 11.1 Hz, 1 H), 4.42 (d, *J* = 11.1 Hz, 1 H), 4.59 (ddd, *J* = 4.9, 1.6, 1.6 Hz, 2 H), 4.68 (dd, *J* = 10.1, 5.4 Hz, 1 H), 5.03–5.13 (m, 3 H), 5.26 (ddt, *J* = 10.6, 1.6, 1.6 Hz, 1 H), 5.45 (ddt, *J* = 17.2, 1.6, 1.6 Hz, 1 H), 5.75 (ddt, *J* = 17.1, 10.2, 7.0 Hz, 1 H), 6.03 (ddt, *J* = 17.2, 10.5 Hz, *J* = 4.9 Hz, 1 H), 6.86–6.94 (m, 3 H), 7.17 (dd, *J* = 8.4, 2.0 Hz, 1 H), 7.23 (d, *J* = 8.5 Hz, 1 H), 7.63 (d, *J* = 2.0 Hz, 1 H) ppm. ^13^C-NMR (125 MHz, 373 K, DMSO-D_6_): δ = 13.7, 17.4, 20.0, 27.5, 27.9, 31.2, 31.2, 32.6, 33.4, 36.4, 38.1, 39.9, 40.3, 54.7, 59.6, 69.1, 70.0, 71.2, 77.9, 78.9, 86.0, 112.7, 113.3, 116.6, 117.0, 128.5, 129.5, 130.6, 131.8, 132.8, 133.1, 138.7, 154.1, 155.2, 158.4, 169.3, 176.7 ppm. HRMS (ESI-ToF) calcd. for C_41_H_59_INO_9_^+^ [M+H]^+^: 836.3229 found 836.3228.

#### 4.2.7. Synthesis of (4R,6S,7R,9S,11S)-12-{[2-(tert-Butoxy)-2-oxoethyl]amino}-6-[(4-methoxybenzyl)oxy]-7,9,11-trimethyl-12-oxo-1-(trimethylsilyl)dodec-1-yn-4-yl (R)-3-[4-(allyloxy)-3-iodophenyl]-2-[(tert-butoxycarbonyl)(methyl)amino]propanoate (**7a**)

Acid **6a** (2.42 g, 2.67 mmol, 1.0 equiv) and *tert*-butyl glycinate hydrochloride (595 mg, 3.55 mmol, 1.33 equiv) were dissolved in anhydrous DMF (13.3 mL, 0.2 M). NEt_3_ (930 µL, 6.67 mmol, 0.726 gml^−1^, 2.5 equiv) and diethyl cyanophosphonate (DCNP) (1.03 mL, 6.14 mmol, 1.075 gml^−1^, 2.3 equiv) were added at 0 °C. After 1 h, brine and diethyl ether were added and the aqueous phase extracted twice with diethyl ether. The combined organic extracts were dried over Na_2_SO_4_ and the solvent removed under reduced pressure. After chromatographic purification (silica, *n*-pentane:EtOAc 8:2–7:3), amide **7a** (2.57 g, 2.52 mmol, 95%) was obtained as colorless resin. R_f_ (**7a**) = 0.33 (silica, *n*-pentane:EtOAc 7:3). ${\left[\alpha \right]}_{D}^{20}$ = −9.9 (c = 1.0, CHCl_3_). Major rotamer: ^1^H-NMR (500 MHz, CDCl_3_): δ = 0.14 (s, 9 H), 0.84 (m, 3 H), 0.90 (d, *J* = 6.3 Hz, 3 H), 0.99 (m, 2 H), 1.10 (m, 1 H), 1.38 (s, 9 H), 1.43–1.52 (m, 10 H), 1.65–1.85 (m, 3 H), 2.03 (m, 1 H), 2.38 (m, 1 H), 2.55 (m, 2 H), 2.68 (s, 3 H), 2.87 (dd, *J* = 14.2, 10.5 Hz, 1 H), 3.08–3.28 (m, 2 H), 3.79 (s, 3 H), 3.91 (m, 2 H), 4.23 (m, 1 H), 4.41–4.55 (m, 3 H), 4.73 (dd, *J* = 10.5, 5.4 Hz, 1 H), 5.19 (m, 1 H), 5.28 (m, 1 H), 5.47 (m, 1 H), 5.95–5.92 (m, 2 H), 6.78 (d, *J* = 8.5 Hz, 1 H), 6.87 (m, 2 H), 7.12 (d, *J* = 7.9 Hz, 1 H), 7.26 (m, 2 H), 7.60 (s, 1 H) ppm. ^13^C-NMR (125 MHz, CDCl_3_): δ = 0.0, 14.0, 19.0, 21.1, 25.9, 28.0, 28.3, 41.7, 32.3, 33.5, 33.5, 33.9, 39.0, 41.0, 41.0, 41.9, 55.3, 59.9, 69.9, 70.7, 71.0, 78.6, 79.9, 82.0, 86.5, 87.2, 102.4, 112.5, 113.8, 117.5, 129.5, 129.9, 131.0, 132.0, 132.7, 139.8, 155.6, 156.0, 159.2, 169.2, 170.3, 176.4 (C-1) ppm. Minor rotamer: (selected signals) ^1^H-NMR (500 MHz, CDCl_3_): δ = 1.31 (s, 9 H), 2.74 (s, 3 H), 3.79 (s, 3 H), 7.03 (d, *J* = 7.9 Hz, 1 H), 7.61 (s, 1 H) ppm. ^13^C-NMR (125 MHz, CDCl_3_): δ = 13.8, 28.5, 31.2, 60.6, 70.4, 80.2, 86.5, 87.6, 130.8, 139.8, 154.9, 156.1, 170.2, 176.3 ppm. HRMS (ESI-ToF) calcd. for C_50_H_75_IN_2_O_10_Si^+^ [M+H]^+^: 1019.4308 found 1019.4310.

#### 4.2.8. Synthesis of (4R,6S,7R,9S,11S)-12-{[2-(tert-Butoxy)-2-oxoethyl]amino}-6-[(4-methoxybenzyl)oxy]-7,9,11-trimethyl-12-oxododec-1-en-4-yl (R)-3-[4-(allyloxy)-3-iodophenyl]-2-[(tert-butoxycarbonyl)(methyl)amino]propanoate (**7b**)

Acid **6b** (1.19 g, 1.31 mmol, 1.0 equiv) and *tert*-butyl glycinate hydrochloride (293 mg, 1.75 mmol, 1.33 equiv) were dissolved in anhydrous DMF (6.56 mL, 0.2 M). NEt_3_ (457 µL, 3.28 mmol, 0.726 gml^−1^, 2.5 equiv) and diethyl cyanophosphonate (DCNP) (509 µL, 1.06 mmol, 1.075 gml^−1^, 2.3 equiv) were added at 0 °C. After 2 h, brine and diethyl ether were added and the aqueous phase extracted twice with diethyl ether. The combined organic extracts were dried over Na_2_SO_4_ and the solvent removed under reduced pressure. After chromatographic purification (silica, *n*-pentane:EtOAc 8:2), amide **7b** (1.01 g, 1.06 mmol, 81%) was obtained as colorless resin. R_f_ (**7b**) = 0.43 (silica, *n*-pentane:EtOAc 7:3). ${\left[\alpha \right]}_{D}^{20}$ = –4.2 (c = 1.0, CHCl_3_). ^1^H-NMR (500 MHz, 373 K, DMSO-D_6_): δ = 0.82 (d, *J* = 6.7 Hz, 3 H), 0.90 (d, *J* = 6.6 Hz, 3 H), 0.91–0.98 (m, 2 H), 1.04 (d, *J* = 6.9 Hz, 3 H), 1.16 (ddd, *J* = 13.4, 8.0, 5.0 Hz, 1 H), 1.35 (s, 9 H), 1.42 (s, 9 H), 1.48 (m, 1 H), 1.61 (m, 2 H), 1.71 (ddd, *J* = 13.5, 9.3, 4.3 Hz, 1 H), 1.97 (m, 1 H), 2.34 (m, 2 H), 2.43 (ddq, *J* = 9.3, 6.9, 5.0 Hz, 1 H), 2.67 (s, 3 H), 2.92 (dd, *J* = 14.3, 10.5 Hz, 1 H), 3.11 (dd, *J* = 14.3, 5.5 Hz, 1 H), 3.27 (m, 1 H), 3.67 (dd, *J* = 17.1, 6.0 Hz, 1 H), 3.72 (dd, *J* = 17.1, 6.0 Hz, 1 H), 3.77 (s, 3 H), 4.27 (d, *J* = 11.1 Hz, 1 H), 4.43 (d, = 11.1 Hz, 1 H), 4.58 (ddd, *J* = 4.9, 1.6, 1.6 Hz, 2 H), 4.69 (dd, *J* = 10.5, 5.5 Hz, 1 H), 5.02–5.13 (m, 3 H), 5.26 (ddt, *J* = 10.6, 1.6, 1.6 Hz, 1 H), 5.45 (ddt, *J* = 17.3, 1.8, 1.6 Hz, 1 H), 5.75 (ddt, *J* = 17.1, 10.2, 7.0 Hz, 1 H), 6.03 (ddt, *J* = 17.3, 10.5, 5.1 Hz, 1 H), 6.86–6.92 (m, 3 H), 7.17 (dd, *J* = 8.4, 2.1 Hz, 1 H), 7.23 (d, *J* = 8.7 Hz, 2 H), 7.63 (d, *J* = 2.0 Hz, 1 H), 7.71 (dd, *J* = 6.0, 6.0 Hz, 1 H) ppm. ^13^C-NMR (125 MHz, 373 K, DMSO-D_6_): δ = 13.6, 18.1, 20.2, 27.3, 27.5, 27.6, 31.2, 31.2, 32.6, 33.4, 37.0, 38.1, 40.2, 40.4, 41.0, 54.7, 59.6, 69.1, 70.0, 71.2, 78.1, 78.7, 79.8, 85.9, 112.7, 113.3, 116.5, 117.0, 128.5, 129.5, 130.6, 131.8, 132.7, 133.1, 138.7, 154.1, 155.2, 158.4, 168.4, 169.3, 175.4 ppm. HRMS (ESI-ToF) calcd. for C_47_H_70_IN_2_O_10_^+^ [M+H]^+^: 949.4070 found 949.4059.

#### 4.2.9. Synthesis of (3R,9S,11S,13R,14S,16R)-3-[4-(Allyloxy)-3-iodobenzyl]-14-hydroxy-4,9,11,13-tetramethyl-16-[3-(trimethylsilyl)prop-2-yn-1-yl]-1-oxa-4,7-diazacyclohexadecane-2,5,8-trione (**7a-1**)

A solution of linear precursor **7a** (874 mg, 858 µmol, 1.0 equiv) in anhydrous DCM (3.86 mL, 4.5 mL/mmol, 0.22 M) was treated with TFA (2.57 mL, 3.0 mL/mmol, 0.33 M). It was warmed to rt and stirred for 90 min. The solvent was removed in N_2_ stream and azeotropically distilled with benzene and dried in vacuo. Triethylamine (1.20 mL, 8.58 mmol, 10.0 equiv) and BOP-Cl (1.09 g, 4.29 mmol, 5.0 equiv) were dissolved in DCM (1.72 l, 0.5 mM) and cooled to 0 °C. The previously obtained residue was dissolved in DCM (172 mL, 200 mL/mmol) and added dropwise (overnight) to the solution at 0 °C. After complete addition, the mixture was warmed to rt and further stirred for 24 h. The reaction mixture was worked up with 0.1 M HCl and the organic phase washed with saturated NaHCO_3_, saturated NH_4_Cl and brine. The organic phase was dried over Na_2_SO_4_ and the solvent removed under reduced pressure. The residue was dissolved in MeOH (51.5 mL, 60 mL/mmol, 0.017 M) and ammonia (4.29 mL, 5 mL/mmol, 35% in H_2_O) was added dropwise and stirred for 1 h. The solvent was removed under reduced pressure, the residue taken up on Isolute, reversed-phase chromatographed (Telos C18, H_2_O:MeCN 80:20–MeCN) and **7a-1** (480 mg, 662 µmol, 77%) obtained as colorless powder after lyophilization. A sample was further purified by preparative HPLC (Luna C18, H_2_O:MeCN 60:40–MeCN) for analytical purposes. R_f_ (**7a-1**) = 0.33 (silica, *n*-pentane:EtOAc 1:1). ${\left[\alpha \right]}_{D}^{20}$ = −19.0 (c = 1.0, CHCl_3_). ^1^H-NMR (400 MHz, CDCl_3_): δ = 0.18 (s, 9 H), 0.83 (d, *J* = 6.7 Hz, 3 H), 0.95 (d, *J* = 6.1 Hz, 3 H), 0.98–1.09 (m, 3 H), 1.11 (d, *J* = 6.6 Hz, 3 H), 1.19 (m, 1 H), 1.37 (ddd, *J* = 14.3, 11.2, 1.7 Hz, 1 H), 1.45–1.58 (m, 2 H), 1.98 (m, 1 H), 2.24 (bs, 1 H), 2.38 (ddq, *J* = 12.0, 6.6, 3.3 Hz, 1 H), 2.54 (d, = 6.1 H, 2 H), 2.89 (dd, *J* = 15.5, 12.0 Hz, 1 H), 2.93 (s, 3 H), 3.27 (dd, *J* = 17.0, 1.0 Hz, 1 H), 3.42 (dd, *J* = 15.4, 4.5 Hz, 1 H), 3.61 (ddd, *J* = 11.2, 3.8, 1.7 Hz, 1 H), 4.55 (ddd, *J* = 4.8, 1.6, 1.6 Hz, 2 H), 4.77 (dd, *J* = 17.0, 8.6 Hz, 1 H), 5.26 (ddt, *J* = 11.4, 6.1, 1.8 Hz, 1 H), 5.30 (ddt, *J* = 10.6, 1.6, 1.3 Hz, 1 H), 5.47 (dd, *J* = 12.0, 4.5 Hz, 1 H), 5.50 (ddt, *J* = 17.1, 1.6, 1.3 Hz, 1 H), 6.03 (ddt, *J* = 17.2, 10.4, 5.0 Hz, 1 H), 6.21 (d, *J* = 8.1 Hz, 1 H), 6.71 (d, *J* = 8.4 Hz, 1 H), 7.09 (dd, *J* = 8.4, 2.0 Hz, 1 H), 7.59 (d, *J* = 2.0 Hz, 1 H) ppm. ^13^C-NMR (100 MHz, CDCl_3_): δ = 0.0, 14.3, 17.6, 18.3, 26.4, 27.0, 30.5, 32.6, 33.1, 34.5, 39.0, 39.8, 42.7, 45.0, 57.8, 65.7, 69.7, 70.6, 86.8, 87.2, 102.0, 112.4, 117.7, 129.0, 130.7, 132.4, 139.1, 156.1, 171.0, 171.7, 177.4 ppm. HRMS (ESI-ToF) calcd. for C_33_H_50_IN_2_O_6_Si^+^ [M+H]^+^: 725.2477 found 725.2482.

#### 4.2.10. Synthesis of (3R,9S,11S,13R,14S,16R)-16-allyl-3-[4-(allyloxy)-3-iodobenzyl]-14-hydroxy-4,9,11,13-tetramethyl-1-oxa-4,7-diazacyclohexadecane-2,5,8-trione (**7b-1**)

A solution of linear precursor **7b** (914 mg, 963 µmol, 1.0 equiv) in anhydrous DCM (4.33 mL, 4.5 mL/mmol, 0.22 M) was treated with TFA (2.89 mL, 3.0 mL/mmol, 0.33 M). It was warmed to rt and stirred for 90 min. The solvent was removed in N_2_ stream and azeotropically distilled with benzene and dried in vacuo. Triethylamine (1.34 mL, 9.63 mmol, 10.0 equiv) and BOP-Cl (1.23 g, 4.82 mmol, 5.0 equiv) were dissolved in DCM (1.93 l, 0.5 mM) and cooled to 0 °C. The previously obtained residue was dissolved in DCM (193 mL, 200 mL/mmol) and added dropwise (overnight) to the solution at 0 °C. After complete addition, the mixture was warmed to rt and further stirred for 24 h. The reaction mixture was worked up with 0.1 M HCl and the organic phase washed with saturated NaHCO_3_, saturated NH_4_Cl and brine. The organic phase was dried over Na_2_SO_4_ and the solvent removed under reduced pressure. The residue was dissolved in MeOH (57.8 mL, 60 mL/mmol, 0.017 M) and ammonia (4.82 mL, 5 mL/mmol, 35% in H_2_O) was added dropwise and stirred for 1 h. The solvent was removed under reduced pressure, the residue taken up on Isolute, reversed-phase chromatographed (FlashPure Select C18, H_2_O:MeCN 90:10–MeCN), and **7b-1** (511 mg, 781 µmol, 81%) obtained as colorless powder after lyophilization. A sample was further purified by preparative HPLC (Luna C18, H_2_O:MeCN 90:10–MeCN) for analytical purposes. ${\left[\alpha \right]}_{D}^{20}$ = −33.7 (c = 1.0, CHCl_3_). ^1^H-NMR (400 MHz, CDCl_3_): δ = 0.82 (d, *J* = 6.7 Hz, 3 H), 0.95 (d, *J* = 6.1 Hz, 3 H), 0.98–1.08 (m, 3 H), 1.18 (m, 1 H), 1.31 (ddd, *J* = 13.9, 11.4, 2.0 Hz, 1 H), 1.42–1.55 (m, 2 H), 1.98 (m, 1 H), 2.07 (bs, 1 H), 2.35–2.43 (m, *J* = 6.7, 6.7 Hz, 3 H), 2.83 (dd, *J* = 15.3, 12.1 Hz, 1 H), 2.87 (s, 3 H), 3.24 (dd, *J* = 16.7, 1.0 Hz, 1 H), 3.38 (dd, *J* = 15.4, 4.5 Hz, 1 H), 3.60 (ddd, *J* = 11.4, 3.9, 1.8 Hz, 1 H), 4.54 (ddd, *J* = 4.9, 1.6, 1.6 Hz, 2 H), 4.78 (dd, *J* = 16.9, 8.7 Hz, 1 H), 5.04–5.14 (m, 2 H), 5.22–5.33 (m, *J* = 10.6, 1.5, 1.5 Hz, 2 H), 5.44 (dd, *J* = 12.2, 4.6 Hz, 1 H), 5.49 (dd, *J* = 17.3, 1.5, 1.5 Hz, 1 H), 5.74 (ddt, *J* = 17.0, 10.2, 6.9 Hz, 1 H), 6.03 (ddt, *J* = 17.2, 10.4, 5.0 Hz, 1 H), 6.24 (d, *J* = 8.3 Hz, 1 H), 6.70 (d, *J* = 8.4 Hz, 1 H), 7.08 (dd, *J* = 8.4, 2.0 Hz, 1 H), 7.58 (d, *J* = 2.0 Hz, 1 H) ppm. ^13^C-NMR (100 MHz, CDCl_3_): δ = 14.3, 17.6, 18.3, 27.0, 30.6, 32.6, 33.2, 34.4, 39.0, 39.7, 39.8, 42.7, 44.9, 57.7, 65.6, 69.7, 71.7, 86.7, 112.4, 117.7, 118.0, 128.9, 130.7, 132.4, 133.8, 139.4, 156.1, 171.3, 171.6, 177.5 ppm. HRMS (ESI-ToF) calcd. for C_30_H_44_IN_2_O_6_^+^ [M+H]^+^: 655.2239 found 655.2247.

#### 4.2.11. Synthesis of (3R,9S,11S,13R,14S,16R)-14-Hydroxy-3-(4-hydroxy-3-iodobenzyl)-4,9,11,13-tetramethyl-16-[3-(trimethylsilyl)prop-2-yn-1-yl]-1-oxa-4,7-diazacyclohexadecane-2,5,8-trione (**8a**)

0.01 M catalyst solution: CpRu(II)(MeCN)_3_PF_6_ (16.4 mg, 37.8 µmol) was dissolved in anhydrous MeOH (3.42 mL) and stirred for 5 min (yellow solution). Quinoline-2-carboxylic acid (380 µL, 38 µmol, 0.1 M in anhydrous MeOH) was added and the mixture stirred for 30 min (dark red solution). Deprotection: Allyl ether **7a-1** (413 mg, 569 µmol, 1.0 equiv) was dissolved in anhydrous degassed MeOH (11.4 mL, 0.05 M) under an N_2_-atmosphere. The previously prepared catalyst solution (2.85 mL, 28.5 µmol, 0.01 M) was added and the mixture stirred for 6 h at rt. The solvent was removed under reduced pressure, the residue taken up on Isolute and chromatographed (SiO_2_, *n*-pentane:EtOAc 1:1–4:6) and alcohol **8a** (340 mg, 496 µmol, 87%) obtained as colorless powder after lyophilization. R_f_ (**8a**) = 0.13 (silica, *n*-pentane: EtOAc 1:1). ${\left[\alpha \right]}_{D}^{20}$ = −20.1 (c = 1.0, CHCl_3_). ^1^H-NMR (400 MHz, CDCl_3_): δ = 0.18 (s, 9 H), 0.84 (d, *J* = 6.7 Hz, 3 H), 0.94 (d, *J* = 6.0 Hz, 3 H), 1.01–1.10 (m, 3 H), 1.12 (d, *J* = 6.6 Hz, 3 H), 1.15–1.23 (m, 1 H), 1.42 (m, 1 H), 1.48–1.60 (m, 2 H), 1.98 (m, 1 H), 2.31 (bs, 1 H), 2.43 (m, 1 H), 2.55 (m, 2 H), 2.87 (dd, *J* = 15.2, 12.0 Hz, 1 H), 2.97 (s, 3 H), 3.21 (d, *J* = 16.6 Hz, 1 H), 3.42 (dd, *J* = 15.2, 4.3 Hz, 1 H), 3.63 (m, 1 H), 4.78 (dd, *J* = 16.7, 8.7 Hz, 1 H), 5.27 (dddd, *J* = 10.2, 6.6, 6.6, 1.6 Hz, 1 H), 5.52 (dd, *J* = 11.9, 4.4 Hz, 1 H), 6.36 (d, *J* = 6.8 Hz, 1 H), 6.83 (d, *J* = 8.3 Hz, 1 H), 6.95 (bs, 1 H), 7.05 (dd, *J* = 8.3, 1.1 Hz, 1 H), 7.47 (d, *J* = 1.1 Hz, 1 H) ppm. ^13^C-NMR (100 MHz, CDCl_3_): δ = 0.0, 14.3, 17.6, 18.4, 26.4, 27.1, 30.8, 32.6, 33.0, 34.6, 39.1, 39.6, 42.8, 44.9, 57.8, 65.9, 70.7, 85.2, 87.3, 102.0, 115.2, 129.5, 130.2, 138.2, 154.3, 170.6, 172.1, 177.6 ppm. HRMS (ESI-ToF) calcd. for C_30_H_46_IN_2_O_6_Si^+^ [M+H]^+^: 685.2164 found 685.2162.

#### 4.2.12. Synthesis of (3R,9S,11S,13R,14S,16R)-16-Allyl-14-hydroxy-3-(4-hydroxy-3-iodobenzyl)-4,9,11,13-tetramethyl-1-oxa-4,7-diazacyclohexadecane-2,5,8-trione (**8b**)

0.01 M catalyst solution: CpRu(II)(MeCN)_3_PF_6_ (20.2 mg, 46.5 µmol) was dissolved in anhydrous MeOH (4.23 mL) and stirred for 5 min (yellow solution). Quinoline-2-carboxylic acid (470 µL, 470 µmol, 0.1 M in anhydrous MeOH) was added and the mixture stirred for 30 min (dark red solution). Deprotection: Allyl ether **7b-1** (463 mg, 707 µmol, 1.0 equiv) was dissolved in anhydrous degassed MeOH (14.1 mL, 0.05 M) under an N_2_-atmosphere. The previously prepared catalyst solution (3.53 mL, 35.3 µmol, 0.01 M) was added and the mixture stirred for 90 min at rt. The solvent was removed under reduced pressure, the residue taken up on Isolute and chromatographed (SiO_2_, *n*-pentane:EtOAc 1:1–2:8). The obtained residue was further purified by preparative HPLC (Luna C18, H_2_O:MeCN 50:50–MeCN) and compound **8b** (354 mg, 576 µmol, 81%) obtained as colorless powder after lyophilization. R_f_ (**8b**) = 0.19 (silica, *n*-pentane:EtOAc 4:6). ${\left[\alpha \right]}_{D}^{20}$ = −40.3 (c = 1.0, CHCl_3_). ^1^H-NMR (400 MHz, CDCl_3_): δ = 0.83 (d, *J* = 6.9 Hz, 3 H), 0.96 (d, *J* = 6.1 Hz, 3 H), 1.00–1.10 (m, 3 H), 1.13 (d, *J* = 6.6 Hz, 3 H), 1.18 (m, 1 H), 1.25 (bs, 1 H), 1.34 (ddd, *J* = 14.0 Hz, *J* = 11.7, 2.0 Hz, 1 H), 1.48 (ddd, *J*= 14.0, 11.8, 2.0 Hz, 1 H), 1.52 (ddd, *J* = 12.1, 12.1, 1.5 Hz, 1 H), 1.99 (m, 1 H), 2.38 (dd, *J* = 7.0, 6.2 Hz, 2 H), 2.43 (ddd, *J* = 12.1, 6.6 Hz, 3.5 Hz, 1 H), 2.83 (dd, *J* = 15.4 Hz, *J* = 12.2 Hz, 1 H), 2.89 (s, 3 H), 3.23 (dd, *J* = 16.7, 1.6 Hz, 1 H), 3.40 (dd, *J* = 15.5, 4.5 Hz, 1 H), 3.62 (ddd, *J* = 11.6, 4.2, 2.1 Hz, 1 Hz), 4.80 (dd, *J* = 16.8, 8.7 Hz, 1 H), 5.06–5.15 (m, 2 H), 5.28 (ddt, *J* = 11.8, 6.2, 2.0 Hz, 1 H), 5.49 (dd, *J* = 12.1, 4.6 Hz, 1 H), 5.76 (ddt, *J* = 17.1, 10.2, 7.0 Hz, 1 H), 6.05 (bs, 1 H), 6.27 (d, *J* = 8.1 Hz, 1 H), 6.86 (d, *J* = 8.4 Hz, 1 H), 7.05 (dd, *J* = 8.3, 2.1 Hz, 1 H), 7.47 (d, *J* = 2.0 Hz, 1 H) ppm. ^13^C-NMR (125 MHz, CDCl_3_): δ = 14.3, 17.6, 18.4, 27.0, 30.7, 32.7, 33.2, 34.5, 39.1, 39.6, 39.8, 42.9, 44.9, 57.8, 65.8, 71.8, 85.5, 115.2, 118.0, 129.7, 130.4, 133.8, 138.0, 154.0, 171.1, 171.8, 177.6 ppm. HRMS (ESI-ToF) calcd. for C_27_H_40_IN_2_O_6_^+^ [M+H]^+^: 615.1926 found 615.1944.

#### 4.2.13. Synthesis of (3R,9S,11S,13R,14S,16R)-14-Hydroxy-3-(4-hydroxy-3-iodobenzyl)-4,9,11,13-tetramethyl-16-(prop-2-yn-1-yl)-1-oxa-4,7-diazacyclohexadecane-2,5,8-trione (**8c**)

Compound **8a** (314 mg, 458 µmol, 1.0 equiv) was dissolved in anhydrous THF (9.17 mL, 0.05 M). TBAF (504 mL, 504 µmol, 1.1 equiv, 1 M solution in THF) was added at 0 °C and stirred for 15 min. The reaction was worked up with saturated NH_4_Cl and the aqueous phase extracted twice with EtOAc. The combined organic phases were dried over Na_2_SO_4,_ and the solvent removed under reduced pressure. The residue was chromatographed (FlashPure Select C18, H_2_O:MeCN 90:10–MeCN) and alkyne **8c** (281 mg, 458 µmol, quant.) obtained as colorless powder after lyophilization. R_f_ (**8c**) = 0.22 (silica, *n*-pentane: EtOAc 1:1). ${\left[\alpha \right]}_{D}^{20}$ = −35.1 (c = 0.5, CHCl_3_). ^1^H-NMR (500 MHz, CDCl_3_): δ = 0.84 (d, *J* = 6.8 Hz, 3 H), 0.96 (d, *J* = 6.3 Hz, 3 H), 1.02–1.10 (m, 3 H), 1.12 (d, *J* = 6.7 Hz, 3 H), 1.20 (m, 1 H), 1.45–1.57 (m, 3 H), 1.98 (m, 1 H), 2.15 (t, *J* = 2.6 Hz, 1 H), 2.39–2.48 (m, 2 H), 2.61 (ddd, *J* = 17.1, 6.7, 2.6 Hz, 1 H), 2.88 (dd, *J* = 15.5, 11.9 Hz, 1 H), 2.94 (s, 3 H), 3.26 (d, *J* = 16.8 Hz, 1 H), 3.41 (dd, *J* = 15.5, 4.7 Hz, 1 H) 3.67 (m, 1 H), 4.81 (dd, *J* = 16.8, 8.7, 1 H), 5.26 (m, 1 H), 5.55 (dd, *J* = 11.9, 4.7 Hz, 1 H), 6.05 (bs, 1 H), 6.29 (m, 1 H), 6.86 (d, *J* = 8.2 Hz, 1 H), 7.06 (dd, *J* = 8.2, 2.0 Hz, 1 H), 7.49 (d, *J* = 2.0 Hz, 1 H) ppm. ^13^C-NMR (125 MHz, CDCl_3_): δ = 14.3, 17.6, 18.3, 24.9, 27.0, 30.5, 32.4, 32.4, 34.7, 39.1, 39.8, 42.8, 45.0, 57.5, 65.9, 70.1, 71.4, 79.4, 85.5, 115.2, 129.8, 130.4, 138.1, 154.1, 170.6, 171.9, 177.5 ppm. HRMS (ESI-ToF) calcd. for C_27_H_38_IN_2_O_6_^+^ [M+H]^+^: 613.1769 found 613.1767.

### 4.3. Modification of the Doliculide Core Structure

#### 4.3.1. Synthesis of (3R,9S,11S,13R,14S,16R)-16-[(1-Benzyl-1H-1,2,3-triazol-4-yl)methyl]-14-hydroxy-3-(4-hydroxy-3-iodobenzyl)-4,9,11,13-tetramethyl-1-oxa-4,7-diazacyclohexadecane-2,5,8-trione (**9a**)

According to the general procedure for CuAAC, alkyne **8c** (15.3 mg, 25.0 µmol, 1.0 equiv) was dissolved in a 1:1 mixture of *t*-BuOH:H_2_O (500 µL, 0.05 M) and reacted with benzyl azide (4.0 mg, 30.0 µmol, 1.2 equiv), sodium ascorbate (15.0 µL, 15.0 µmol, 1 M in H_2_O, 0.6 equiv), and copper(II) sulfate (12.5 µL, 12.5 µmol, 1 M in H_2_O, 0.5 equiv). After stirring for 16 h, the mixture was concentrated and the obtained crude product was subjected to reversed-phase flash chromatography (FlashPure Select C18, H_2_O:MeCN 90:10–MeCN). Triazole **9a** (17.5 mg, 23.5 µmol, 86%) was obtained as colorless powder after lyophilization. ${\left[\alpha \right]}_{D}^{20}$ = –12.0 (c = 1.0, CHCl_3_). ^1^H-NMR (500 MHz, CDCl_3_): δ = 0.81 (d, *J* = 6.9 Hz, 3 H), 0.94 (d, *J* = 6.1 Hz, 3 H), 1.01–1.09 (m, 3 H), 1.13 (d, *J* = 6.7 Hz, 3 H), 1.17 (m, 1 H), 1.43–1.55 (m, 3 H), 1.97 (m, 1 H), 2.44 (m, 1 H), 2.61 (dd, *J* = 15.3, 12.1 Hz, 1 H), 2.78 (s, 3 H), 2.98–3.08 (m, 2 H), 3.09–3.17 (m, 2 H), 3.63 (ddd, *J* = 9.0, 4.1, 4.1 Hz, 1 H), 4.71 (dd, *J* = 16.7, 8.9 Hz), 5.37 (dd, *J* = 12.1, 4.6 Hz, 1 H), 5.44 (m, 1 H), 5.55 (d, *J* = 14.8 Hz, 1 H), 5.59 (d, *J* = 14.8 Hz, 1 H), 6.34 (dd, *J* = 8.9, 2.4 Hz, 1 H), 6.80 (d, *J* = 8.3 Hz, 1 H), 6.95 (dd, *J* = 8.2, 1.4 Hz, 1 H), 7.28–7.37 (m, 5 H), 7.39 (d, *J* = 1.7 Hz, 1 H), 7.43 (s, 1 H) ppm. ^13^C-NMR (125 MHz, CDCl_3_): δ = 14.3, 17.6, 18.5, 27.0, 30.8, 31.5, 32.3, 33.1, 34.4, 39.1, 39.5, 42.9, 44.8, 54.0, 57.8, 65.8, 71.7, 85.1, 115.1, 121.9, 128.1, 128.7, 129.0, 129.4, 130.2, 135.0, 138.3, 143.9, 154.3, 171.1, 172.0, 177.7 ppm. HRMS (ESI-ToF) calcd. for C_34_H_45_IN_5_O_6_^+^ [M]^+^: 746.2409 found 746.2408.

#### 4.3.2. Synthesis of (3R,9S,11S,13R,14S,16R)-14-hydroxy-3-(4-hydroxy-3-iodobenzyl)-4,9,11,13-tetramethyl-16-((1-pentyl-1H-1,2,3-triazol-4-yl)methyl)-1-oxa-4,7-diazacyclohexadecane-2,5,8-trione (**9b**)

According to the general procedure for CuAAC, alkyne **8c** (12.8 mg, 20.9 µmol, 1.0 equiv) was dissolved in a 1:1 mixture of *t*-BuOH:H_2_O (418 µL, 0.05 M) and reacted with 1-azidopentane (2.8 mg, 25.1 µmol, 1.2 equiv), sodium ascorbate (12.5 µL, 12.5 µmol, 1 M in H_2_O, 0.6 equiv) and copper(II) sulfate (10.5 µL, 10.5 µmol, 1 M in H_2_O, 0.5 equiv). After stirring for 16 h, the mixture was concentrated and the obtained crude product was subjected to reversed-phase flash chromatography (FlashPure Select C18, H_2_O:MeCN 90:10–MeCN). Triazole **9b** (14.4 mg, 19.8 µmol, 95%) was obtained as colorless powder after lyophilization. ${\left[\alpha \right]}_{D}^{20}$ = −9.6 (c = 1.0, CHCl_3_). ^1^H-NMR (500 MHz, CDCl_3_): δ = 0.81 (d, *J* = 6.6 Hz, 3 H), 0.88 (t, *J* = 7.1 Hz, 3 H), 0.94 (d, *J* = 6.0 Hz, 3 H), 0.99–1.08 (m, 3 H), 1.11 (d, *J* = 6.6 Hz, 3 H), 1.19 (m, 1 H), 1.24–1.39 (m, 4 H), 1.42–1.56 (m, 3 H), 1.89 (tt, *J* = 7.3, 7.3 Hz, 2 H), 1.97 (m, 1 H), 2.43 (m, 1 H), 2.70 (dd, *J* = 14.3, 11.2 Hz, 1 H), 2.94 (s, 3 H), 2.98 (dd, *J* = 15.3, 3.8 Hz, 1 H), 3.06 (dd, *J* = 15.3, 8.2 Hz, 1 H), 3.11–3.21 (m, 2 H), 3.53–3.74 (m, 2 H), 4.36 (t, *J* = 7.3 Hz, 2 H), 4.76 (dd, *J* = 16.6, 8.7 Hz, 1 H), 5.33 (dd, *J* = 11.1, 3.9 Hz, 1 H), 5.44 (m, 1 H), 6.44 (bs, 1 H), 6.77 (d, *J* = 7.8 Hz, 1 H), 6.92 (d, *J* = 7.8 Hz, 1 H), 7.40 (s, 1 H), 7.43 (s, 1 H) ppm. ^13^C-NMR (125 MHz, CDCl_3_): δ = 13.9, 14.4, 17.7, 18.4, 22.1, 27.0, 28.6, 30.1, 31.0, 31.6, 32.4, 33.2, 34.5, 39.0, 39.6, 42.8, 44.9, 50.3, 58.0, 65.8, 71.9, 86.0, 115.5, 121.7, 129.5, 129.5, 138.2, 143.5, 155.5, 171.3, 171.9, 177.6 ppm. HRMS (ESI-ToF) calcd. for C_32_H_49_IN_5_O_6_^+^ [M+H]^+^: 726.2722 found 726.2722.

#### 4.3.3. Synthesis of Benzyl 2-(4-{[(3R,9S,11S,13R,14S,16R)-14-hydroxy-3-(4-hydroxy-3-iodobenzyl)-4,9,11,13-tetramethyl-2,5,8-trioxo-1-oxa-4,7-diazacyclohexadecan-16-yl]methyl}-1H-1,2,3-triazol-1-yl)acetate (**9c**)

According to the general procedure for CuAAC, alkyne **8c** (11.5 mg, 18.8 µmol, 1.0 equiv) was dissolved in a 1:1 mixture of *t*-BuOH:H_2_O (374 µL, 0.05 M) and reacted with benzyl 2-azidoacetate (4.3 mg, 22.5 µmol, 1.2 equiv), sodium ascorbate (11.3 µL, 11.3 µmol, 1 M in H_2_O, 0.6 equiv) and copper(II) sulfate (9.4 µL, 9.4 µmol, 1 M in H_2_O, 0.5 equiv). After stirring for 16 h, the mixture was concentrated and the obtained crude product was subjected to reversed-phase flash chromatography (FlashPure Select C18, H_2_O:MeCN 90:10–MeCN). Triazole **9c** (14.2 mg, 17.7 µmol, 94%) was obtained as colorless powder after lyophilization. ${\left[\alpha \right]}_{D}^{20}$ = –13.0 (c = 1.0, CHCl_3_). ^1^H-NMR (500 MHz, CDCl_3_): δ = 0.82 (d, *J* = 6.7 Hz, 3 H), 0.94 (d, *J* = 6.0 Hz, 3 H), 0.99–1.07 (m, 3 H), 1.10 (d, *J* = 6.6 Hz, 3 H), 1.16 (m, 1 H), 1.44–1.51 (m, 2 H), 1.58 (m, 1 H), 2.0 (m, 1 H), 2.42 (ddq, *J* = 12.1, 6.6 Hz, *J* = 3.4 Hz, 1 H), 2.78 (dd, *J* = 15.4, 12.4 Hz, 1 H), 2.80 (s, 3 H), 3.05 (dd, *J* = 16.6, 2.0 Hz, 1 H), 3.09 (dd, *J* = 16.3, 6.4 Hz, 1 H), 3.23 (dd, *J* = 16.3, 3.8 Hz, 1 H), 3.26 (dd, *J* = 15.4, 4.4 Hz, 1 H), 3.68 (m, 1 H), 4.70 (dd, *J* = 16.6, 9.2 Hz, 1 H), 5.19 (d, *J* = 12.2 Hz, 1 H), 5.25 (d, *J* = 12.2 Hz, 1 H), 5.33 (d, *J* = 17.5 Hz, 1 H), 5.37 (d, *J* = 17.5 Hz, 1 H), 5.47 (dd, *J* = 12.2, 4.4 Hz, 1 H), 5.58 (m, 1 H), 6.13 (dd, *J* = 9.2, 2.0 Hz, 1 H), 6.81 (d, *J* = 8.2 Hz, 1 H), 7.01 (dd, *J* = 8.2, 1.4 Hz, 1 H), 7.29–7.39 (m, 5 H), 7.46 (d, *J* = 1.7 Hz, 1 H), 7.68 (s, 1 H) ppm. ^13^C-NMR (125 MHz, CDCl_3_): δ = 14.2, 17.5, 18.5, 26.9, 30.7, 31.3, 32.2, 32.5, 34.2, 39.0 39.3, 43.1, 44.8, 50.8, 57.6, 65.8, 67.8, 71.1, 85.2, 115.1, 123.4, 128.4, 128.7, 128.7, 129.5, 130.4, 134.7, 138.3, 143.4, 154.2, 167.0, 171.2, 172.0, 177.9 ppm. HRMS (ESI-ToF) calcd. for C_29_H_41_IN_5_O_8_^+^ [M–Bn+2H]^+^: 714.1994 found 714.1991.

#### 4.3.4. Synthesis of 2-{2-[2-(4-{[(3R,9S,11S,13R,14S,16R)-14-Hydroxy-3-(4-hydroxy-3-iodobenzyl)-4,9,11,13-tetramethyl-2,5,8-trioxo-1-oxa-4,7-diazacyclohexadecan-16-yl]methyl}-1H-1,2,3-triazol-1-yl)ethoxy]ethoxy}acetic Acid (**9d**)

According to the general procedure for CuAAC, alkyne **8c** (9.9 mg, 16.2 µmol, 1.0 equiv) was dissolved in a 1:1 mixture of *t*-BuOH:H_2_O (324 µL, 0.05 M) and reacted with potassium 2-[2-(2-azidoethoxy)ethoxy)acetate (4.4 mg, 19.4 µmol, 1.2 equiv), sodium ascorbate (9.7 µL, 9.7 µmol, 1 M in H_2_O, 0.6 equiv) and copper(II) sulfate (8.1 µL, 8.1 µmol, 1 M in H_2_O, 0.5 equiv). After stirring for 18 h. DCM and brine were added to the reaction mixture, the phases separated and the aqueous phase extracted twice with DCM and once with CHCl_3_/*i*-PrOH (3:1). The combined organic extracts were concentrated and the obtained crude product was subjected to reversed-phase flash chromatography (FlashPure Select C18, H_2_O + 0.1% HCOOH:MeCN 90:10–MeCN). Triazole **9d** (8.5 mg, 10.6 µmol, 66%) was obtained as colorless powder after lyophilization. ${\left[\alpha \right]}_{D}^{20}$ = −4.1 (c = 1.0, CHCl_3_). ^1^H-NMR (500 MHz, DMSO-D_6_): δ = 0.68 (d, *J* = 6.9 Hz, 3 H), 0.84 (d, *J* = 6.3 Hz, 3 H), 0.87–0.92 (m, 3 H), 0.94 (d, *J* = 6.7 Hz, 3 H), 1.15 (m, 1 H), 1.22–1.38 (m, 3 H), 1.57 (bs, 1 H), 1.77 (m, 1 H), 2.47 (m, 1 H), 2.79 (dd, *J* = 15.0, 11.4 Hz, 1 H), 2.82 (s, 3 H), 2.92 (dd, *J* = 15.0, 4.6 Hz, 1 H), 2.98 (dd, *J* = 15.2, 6.0 Hz, 1 H), 3.01 (dd, *J* = 15.7, 3.1 Hz, 1 H), 3.09 (dd, *J* = 15.0, 4.6 Hz, 1 H), 3.34 (bs, 1 H), 3.51–3.58 (m, 5 H), 3.83 (t, *J* = 5.4 Hz, 2 H), 3.87 (s, 2 H), 4.49–4.60 (m, 3 H), 5.21 (dddd, *J* = 9.2, 6.0, 5.6, 4.6 Hz, 1 H), 5.38 (dd, *J* = 11.4, 4.6 Hz, 1 H), 6.78 (d, *J* = 8.2 Hz, 1 H), 6.97 (dd, *J* = 8.4, 2.0 Hz, 1 H), 7.47 (d, *J* = 2.0 Hz, 1 H), 7.93 (s, 1 H), 8.17 (dd, *J* = 8.6, 3.1 Hz, 1 H), 10.5 (bs, 1 H) ppm. ^13^C-NMR (125 MHz, DMSO-D_6_): δ = 14.4, 17.5, 18.6, 26.2, 30.5, 30.8, 31.0, 31.4, 34.5, 37.1, 38.6, 43.0, 44.7, 49.1, 56.6, 63.9, 68.3, 68.9, 69.5, 69.5, 70.8, 84.4, 114.7, 123.2, 129.6, 130.0, 138.4, 142.0, 155.2, 169.9, 170.7, 171.8, 176.2 ppm. HRMS (ESI-ToF) calcd. for C_33_H_49_IN_5_O_10_^+^ [M+H]^+^: 802.2519 found 802.2518.

#### 4.3.5. Synthesis of (3R,9S,11S,13R,14S,16R)-14-Hydroxy-3-(4-hydroxy-3-iodobenzyl)-16-((1-(2-(2-(2-hydroxyethoxy)ethoxy)ethyl)-1H-1,2,3-triazol-4-yl)methyl)-4,9,11,13-tetramethyl-1-oxa-4,7-diazacyclohexadecane-2,5,8-trione (**9e**)

According to the general procedure for CuAAC, alkyne **8c** (14.8 mg, 24.2 µmol, 1.0 equiv) was dissolved in a 1:1 mixture of *t*-BuOH:H_2_O (484 µL, 0.05 M) and reacted with 2-[2-(2-azidoethoxy]ethoxy)ethan-1-ol (5.1 mg, 29.1 µmol, 1.2 equiv), sodium ascorbate (14.5 µL, 14.5 µmol, 1 M in H_2_O, 0.6 equiv) and copper(II) sulfate (12.1 µL, 12.1 µmol, 1 M in H_2_O, 0.5 equiv). After stirring for 16 h, the mixture was concentrated and the obtained crude product was subjected to reversed-phase flash chromatography (FlashPure Select C18, H_2_O + 0.1% HCOOH:MeCN 90:10–MeCN). Triazole **9e** (15.2 mg, 19.3 µmol, 80%) was obtained as colorless powder after lyophilization. ${\left[\alpha \right]}_{D}^{20}$ = –9.6 (c = 1.0, CHCl_3_). ^1^H-NMR (500 MHz, CDCl_3_): δ = 0.82 (d, *J* = 6.0 Hz, 3 H), 0.93 (d, *J* = 6.1 Hz, 3 H), 0.99–1.08 (m, 3 H), 1.12 (d, *J* = 6.6 Hz, 3 H), 1.17 (m, 1 H), 1.42–1.58 (m, 3 H), 1.88–2.07 (m, 3 H), 2.43 (ddq, *J* = 12.1, 6.6, 3.4 Hz, 1 H), 2.71 (dd, *J* = 15.3, 11.8 Hz, 1 H), 2.93 (s, 3 H), 2.99 (dd, *J* = 15.4, 4.1 Hz, 1 H), 3.07 (dd, *J* = 15.0, 7.9 Hz, 1 H), 3.13 (dd, *J* = 16.6, 1.5 Hz, 1 H), 3.17 (dd, *J* = 15.4, 4.6 Hz, 1 H), 3.58 (t, *J* = 4.4 Hz, 2 H), 3.60–3.65 (m, 5 H), 3.75 (dd, *J* = 4.1, 4.1 Hz, 2 H), 3.83–3.92 (m, 2 H), 4.57 (m, 2 H), 4.75 (dd, *J* = 16.6, 8.9 Hz, 1 H), 5.34 (dd, *J* = 11.8, 4.7 Hz, 1 H), 5.43 (m, 1 H), 6.41 (dd, *J* = 8.9, 1.5 Hz, 1 H), 6.82 (d, *J* = 8.1 Hz, 1 H), 6.97 (dd, *J* = 8.1, 1.7 Hz, 1 H), 7.44 (d, *J* = 1.7 Hz, 1 H), 7.73 (s, 1 H) ppm. ^13^C-NMR (125 MHz, CDCl_3_): δ = 14.3, 17.6, 18.5, 27.0, 31.0, 31.5, 32.4, 33.3, 34.4, 39.1, 39.6, 42.9, 44.9, 50.1, 58.0, 61.6, 65.9, 69.5, 70.2, 70.4, 72.2, 72.5, 85.0, 115.2, 123.4, 129.6, 130.2, 138.4, 143.6, 154.4, 171.2, 171.9, 177.8 ppm. HRMS (ESI-ToF) calcd. for C_33_H_51_IN_5_O_9_^+^ [M+H]^+^: 788.2726 found 788.2727.

#### 4.3.6. Synthesis of tert-Butyl (2-{2-[2-(4-{[(3R,9S,11S,13R,14S,16R)-14-hydroxy-3-(4-hydroxy-3-iodobenzyl)-4,9,11,13-tetramethyl-2,5,8-trioxo-1-oxa-4,7-diazacyclohexadecan-16-yl]methyl}-1H-1,2,3-triazol-1-yl)ethoxy]ethoxy}ethyl)carbamate (**9f**)

According to the general procedure for CuAAC, alkyne **8c** (10.2 mg, 16.7 µmol, 1.0 equiv) was dissolved in a 1:1 mixture of *t*-BuOH:H_2_O (334 µL, 0.05 M) and reacted with *tert*-butyl {2-[2-(2-aminoethoxy)ethoxy]ethyl}carbamate (5.5 mg, 20.0 µmol, 1.2 equiv), sodium ascorbate (10.0 µL, 10.0 µmol, 1 M in H_2_O, 0.6 equiv) and copper(II) sulfate (8.3 µL, 8.3 µmol, 1 M in H_2_O, 0.5 equiv). After stirring for 16 h, the mixture was concentrated and the obtained crude product was subjected to reversed-phase flash chromatography (FlashPure Select C18, H_2_O + 0.1% HCOOH:MeCN 90:10–MeCN). Triazole **9f** (13.6 mg, 15.3 µmol, 92%) was obtained as colorless powder after lyophilization. ${\left[\alpha \right]}_{D}^{20}$ = –4.3 (c = 1.0, CHCl_3_). ^1^H-NMR (500 MHz, DMSO-D_6_): δ = 0.68 (d, *J* = 6.7 Hz, 3 H), 0.84 (d, *J* = 6.1 Hz, 3 H), 0.86–0.93 (m, 3 H), 0.94 (d, *J* = 6.7 Hz, 3 H), 1.15 (m, 1 H), 1.22–1.35 (m, 3 H), 1.36 (s, 9 H), 1.77 (m, 1 H), 2.48 (m, 1 H), 2.80 (dd, *J* = 15.0, 11.7 Hz, 1 H), 2.81 (s, 3 H), 2.92 (dd, *J* = 15.1, 5.0 Hz, 1 H), 2.95–3.06 (m, 4 H), 3.09 (dd, *J* = 15.0, 4.3 Hz, 1 H), 3.34 (t, *J* = 6.0 Hz, 2 H), 3.45 (m, 2 H), 3.51 (m, 2 H), 3.57 (m, 1 H), 3.82 (ddd, *J* = 16.2, 10.8, 5.3 Hz, 1 H), 3.83 (ddd, *J* = 16.2, 11.1, 5.2 Hz, 1 H), 4.21 (d, *J* = 4.7 Hz, 1 H), 4.51–4.60 (m, 3 H), 5.21 (m, 1 H), 5.40 (dd, *J* = 11.5, 4.5 Hz, 1 H), 6.68–6.80 (m, 2 H), 6.98 (dd, *J* = 8.2, 1.8 Hz, 1 H), 7.47 (d, *J* = 1.8 Hz, 1 H), 7.91 (s, 1 H), 8.16 (dd, *J* = 8.6, 3.1 Hz, 1 H), 10.1 (bs, 1 H) ppm. ^13^C-NMR (125 MHz, DMSO-D_6_): δ = 14.4, 17.5, 18.6, 26.2, 28.2, 30.4, 30.8, 30.9, 31.3, 34.4, 37.1, 38.5, 39.7, 43.0, 44.7, 49.1, 56.5, 63.8, 68.0, 69.2, 69.4, 69.5, 70.7, 77.6, 84.3, 114.7, 123.2, 129.6, 130.1, 138.5, 141.9, 155.0, 155.6, 169.9, 170.7, 176.1 ppm. HRMS (ESI-ToF) calcd. for C_38_H_60_IN_6_O_10_^+^ [M+H]^+^: 887.3410 found 887.3397.

#### 4.3.7. Synthesis of tert-Butyl [2-(2-{2-[2-(4-{[(3R,9S,11S,13R,14S,16R)-14-hydroxy-3-(4-hydroxy-3-iodobenzyl)-4,9,11,13-tetramethyl-2,5,8-trioxo-1-oxa-4,7-diazacyclohexadecan-16-yl]methyl}-1H-1,2,3-triazol-1-yl)ethoxy]ethoxy}ethoxy)ethyl]carbamate (**9g**)

According to the general procedure for CuAAC, alkyne **8c** (10.2 mg, 16.7 µmol, 1.0 equiv) was dissolved in a 1:1 mixture of *t*-BuOH:H_2_O (334 µL, 0.05 M) and reacted with *tert*-butyl (2-{2-[2-(2-aminoethoxy)ethoxy]ethoxy}ethyl)carbamate (6.4 mg, 20.1 µmol, 1.2 equiv), sodium ascorbate (10.0 µL, 10.0 µmol, 1 M in H_2_O, 0.6 equiv) and copper(II) sulfate (8.3 µL, 8.3 µmol, 1 M in H_2_O, 0.5 equiv). After stirring for 16 h, the mixture was concentrated and the obtained crude product was subjected to reversed-phase flash chromatography (FlashPure Select C18, H_2_O + 0.1% HCOOH:MeCN 90:10–MeCN) and further purified by preparative HPLC (Luna C18, H_2_O + 0.1% HCOOH:MeCN 90:10–MeCN). Triazole **9g** (12.4 mg, 13.3 µmol, 80%) was obtained as colorless powder after lyophilization. ${\left[\alpha \right]}_{D}^{20}$ = –5.3 (c = 1.0, CHCl_3_). ^1^H-NMR (500 MHz, DMSO-D_6_): δ = 0.68 (d, *J* = 6.7 Hz, 3 H), 0.84 (d, *J* = 6.3 Hz, 3 H), 0.86–0.92 (m, 3 H), 0.94 (d, *J* = 6.7 Hz, 3 H), 1.16 (m, 1 H), 1.22–1.34 (m, 3 H), 1.36 (s, 9 H), 1.77 (m, 1 H), 2.48 (m, 1 H), 2.75–2.82 (m, *J* = 15.3, 11.7 Hz, 4 H), 2.92 (dd, *J* = 15.1, 4.9 Hz, 1 H), 2.95–3.03 (m, 2 H), 3.04 (dt, *J* = 6.1, 6.1 Hz, 2 H), 3.09 (dd, *J* = 15.1, 4.6 Hz, 1 H), 3.36 (t, *J* = 6.1 Hz, 2 H), 3.45–3.47 (m, 4 H), 3.48 (m, 2 H), 3.51 (m, 2 H), 3.57 (m, 1 H), 3.83 (ddd, *J* = 16.3, 10.8, 5.2 Hz, 1 H), 3.83 (ddd, *J* = 16.3, 11.1, 5.2 Hz, 1 H), 4.21 (d, *J* = 4.0 Hz, 1 H), 4.49–4.61 (m, 3 H), 5.22 (m, 1 H), 5.40 (dd, *J* = 11.7, 4.5 Hz, 1 H), 6.68–6.77 (m, 2 H), 6.98 (dd, *J* = 8.3, 1.9 Hz, 1 H), 7.47 (d, *J* = 1.8 Hz, 1 H), 7.91 (s, 1 H), 8.16 (dd, *J* = 8.7, 3.2 Hz, 1 H), 10.2 (bs, 1 H) ppm. ^13^C-NMR (125 MHz, DMSO-D_6_): δ = 14.4, 17.5, 18.6, 26.2, 28.2, 30.4, 30.8, 30.9, 31.3, 34.4, 37.1, 38.5, 39.7, 43.0, 44.7, 49.1, 56.5, 63.8, 69.0, 69.2, 69.5, 69.5, 69.6, 69.7, 70.7, 77.6, 84.3, 114.7, 123.2, 129.6, 130.0, 138.5, 141.9, 155.1, 155.6, 169.9, 170.7, 176.1 ppm. HRMS (ESI-ToF) calcd. for C_40_H_64_IN_6_O_11_^+^ [M+H]^+^: 931.3672 found 931.3672.

#### 4.3.8. Synthesis of (3R,9S,11S,13R,14S,16R)-14-Hydroxy-3-(4-hydroxy-3-iodobenzyl)-4,9,11,13-tetramethyl-16-(3-phenylprop-2-yn-1-yl)-1-oxa-4,7-diazacyclohexadecane-2,5,8-trione (**10a**)

According to the general procedure for Sonogashira couplings, alkyne **8c** (10.5 mg, 17.1 µmol) was reacted with (PPh_3_)_2_PdCl_2_ (1.2 mg, 1.71 µmol, 0.1 equiv), iodobenzene (17.5 mg, 85.7 µmol, 5.0 equiv) and copper(I) iodide (0.7 mg, 3.4 µmol, 0.2 equiv) in NEt_3_ (114 µL, 0.15 M) and stirred for 16 h. The obtained crude product was subjected to flash chromatography (RediSep Rf Silica, DCM–DCM:MeOH 90:10) and further purified by preparative HPLC (Luna C18, H_2_O:MeCN 90:10–MeCN). Alkyne **10a** (6.8 mg, 9.9 µmol, 58%) was obtained as colorless powder after lyophilization. ${\left[\alpha \right]}_{D}^{20}$ = –0.3 (c = 1.0, CHCl_3_). ^1^H-NMR (500 MHz, CDCl_3_): δ = 0.86 (d, *J* = 6.9 Hz, 3 H), 0.97 (d, *J* = 6.3 Hz, 3 H), 1.02–1.11 (m, 3 H), 1.13 (d, *J* = 6.7 Hz, 3 H), 1.20 (m, 1 H), 1.44 (ddd, *J* = 13.7, 11.4, 1.8 Hz, 1 H), 1.54 (m, 1 H), 1.60 (ddd, *J* = 13.7, 11.4 Hz, 1.8 Hz, 1 H), 2.02 (m, 1 H), 2.35 (bs, 1 H), 2.41 (ddq, *J* = 12.0, 6.6, 3.4 Hz, 1 H), 2.73 (d, *J* = 6.3 Hz, 1 H), 2.76 (dd, *J* = 15.6, 12.2 Hz, 1 H), 2.90 (s, 3 H), 3.25 (dd, *J* = 16.9, 1.7 Hz, 1 H), 3.35 (dd, *J* = 15.6, 4.6 Hz, 1 H), 3.66 (m, 1 H), 4.80 (dd, *J* = 16.8, 8.7 Hz, 1 H), 5.38 (ddt, *J* = 11.4, 6.4, 1.8 Hz, 1 H), 5.46 (dd, *J* = 12.1, 4.7 Hz, 1 H), 5.58 (bs, 1 H), 6.23 (dd, *J* = 8.7, 1.7 Hz, 1 H), 6.84 (d, *J* = 8.2 Hz, 1 H), 6.92 (dd, *J* = 8.3, 1.8 Hz, 1 H), 7.28 (d, *J* = 1.8 Hz, 1 H), 7.32–7.37 (m, 3 H), 7.43 (m, 2 H) ppm. ^13^C-NMR (125 MHz, CDCl_3_): δ = 14.3, 17.6, 18.4, 26.1, 27.1, 30.5, 32.6, 33.3, 34.5, 39.1, 39.7, 42.8, 45.0, 57.7, 65.9, 70.9, 82.8, 85.3, 85.7, 115.1, 123.1, 128.2, 128.5, 129.6, 130.4, 131.6, 137.8, 153.9, 171.2, 171.9, 177.5 ppm. HRMS (ESI-ToF) calcd. for C_33_H_42_IN_2_O_6_^+^ [M+H]^+^: 689.2062 found 689.2061.

#### 4.3.9. Synthesis of (3R,9S,11S,13R,14S,16R)-14-Hydroxy-3-(4-hydroxy-3-iodobenzyl)-16-[3-(4-methoxyphenyl)prop-2-yn-1-yl]-4,9,11,13-tetramethyl-1-oxa-4,7-diazacyclohexadecane-2,5,8-trione (**10b**)

According to the general procedure for Sonogashira couplings, alkyne **8c** (11.3 mg, 18.4 µmol) was reacted with (PPh_3_)_2_PdCl_2_ (1.3 mg, 1.85 µmol, 0.1 equiv), 1-iodo-4-methoxybenzene (21.6 mg, 92.3 µmol, 5.0 equiv) and copper(I) iodide (0.7 mg, 3.7 µmol, 0.2 equiv) in NEt_3_ (123 µL, 0.15 M) and stirred for 16 h. The obtained crude product was subjected to flash chromatography (RediSep Rf Silica, DCM–DCM:MeOH 9:1). Alkyne **10b** (10.0 mg, 13.9 µmol, 75%) was obtained as colorless powder after lyophilization. ${\left[\alpha \right]}_{D}^{20}$ = +8.8 (c = 1.0, CHCl_3_). ^1^H-NMR (500 MHz, CDCl_3_): δ = 0.85 (d, *J* = 6.9 Hz, 1 Hz, 3 H), 0.96 (d, *J* = 6.1 Hz, 3 H), 1.00–1.11 (m, 3 H), 1.13 (d, *J* = 6.6 Hz, 3 H), 1.19 (m, 1 H), 1.44 (m, 1 H), 1.54 (m, 1 H), 1.59 (m, 1 H), 2.01 (m, 1 H), 2.35–2.46 (m, *J* = 12.2, 6.6 Hz, 3.4 Hz, 1 H), 2.71 (d, *J* = 6.3 Hz, 2 H), 2.77 (dd, *J* = 15.6, 12.2 Hz, 1 H), 2.91 (s, 3 H), 3.23 (dd, *J* = 16.8, 1.8 Hz, 1 H), 3.35 (dd, *J* = 15.6, 4.6 Hz, 1 H), 3.66 (m, 1 H), 3.82 (s, 3 H), 4.80 (dd, *J* = 16.8, 8.7 Hz, 1 H), 5.36 (ddt, *J* = 11.4, 6.3, 2.0 Hz, 1 H), 5.47 (dd, *J* = 12.2, 4.6 Hz, 1 H), 6.12 (bs, 1 H), 6.29 (dd, *J* = 8.7, 1.8 Hz, 1 H), 6.83 (d, *J* = 8.4 Hz, 1 H), 6.87 (m, 2 H), 6.93 (dd, *J* = 8.4, 2.0 Hz, 1 H), 7.29 (d, *J* = 2.0 Hz, 1 H), 7.36 (m, 2 H) ppm. ^13^C-NMR (125 MHz, CDCl_3_): δ = 14.3, 17.6, 18.4, 26.1, 27.1, 30.5, 32.6, 33.2, 34.5, 39.1, 39.7, 42.8, 44.9, 55.3, 57.7, 65.9, 71.0, 82.6, 83.7, 85.4, 114.1, 115.1, 115.2, 129.5, 130.3, 133.0, 138.0, 154.1, 159.5, 171.1, 172.0, 177.5 ppm. HRMS (ESI-ToF) calcd. for C_34_H_44_IN_2_O_7_^+^ [M+H]^+^: 719.2188 found 719.2192.

#### 4.3.10. Synthesis of (3R,9S,11S,13R,14S,16R)-14-Hydroxy-3-(4-hydroxy-3-iodobenzyl)-4,9,11,13-tetramethyl-16-[3-(4-nitrophenyl)prop-2-yn-1-yl]-1-oxa-4,7-diazacyclohexadecane-2,5,8-trione (**10c**)

According to the general procedure for Sonogashira couplings, alkyne **8c** (14.4 mg, 23.5 µmol) was reacted with (PPh_3_)_2_PdCl_2_ (1.7 mg, 2.35 µmol, 0.1 equiv), 1-iodo-4-nitrobenzene (29.3 mg, 118 µmol, 5.0 equiv) and copper(I) iodide (0.9 mg, 4.7 µmol, 0.2 equiv) in NEt_3_ (157 µL, 0.15 M) and stirred for 16 h. The obtained crude product was subjected to flash chromatography (RediSep Rf Silica, DCM–DCM:MeOH 9:1). Alkyne **10c** (13.1 mg, 17.9 µmol, 76%) was obtained as colorless powder after lyophilization. ${\left[\alpha \right]}_{D}^{20}$ = +11.7 (c = 1.0, CHCl_3_). ^1^H-NMR (500 MHz, CDCl_3_): δ = 0.86 (d, *J* = 6.7 Hz, 3 H), 0.96 (d, *J* = 6.3 Hz, 3 H), 1.04–1.13 (m, 3 H), 1.14 (d, *J* = 6.6 Hz, 3 H), 1.21 (m, 1 H), 1.51–1.63 (m, 3 H), 1.99 (m, 1 H), 2.12 (bs, 1 H), 2.44 (ddq, *J* = 12.0, 6.6, 3.4 Hz, 1 H), 2.74–2.81 (m, 2 H), 2.87 (dd, *J* = 17.4, 5.3 Hz, 1 H), 2.92 (s, 3 H), 3.17 (dd, *J* = 16.6, 2.1 Hz, 1 H), 3.39 (dd, *J* = 15.3, 4.6 Hz, 1 H), 3.72 (m, 1 H), 4.78 (dd, *J* = 16.6, 8.8 Hz, 1 H), 5.38 (m, 1 H), 5.56 (dd, *J* = 11.9, 4.7 Hz, 1 H), 6.36 (dd, *J* = 8.8, 2.1 Hz, 1 H), 6.70–6.91 (m, *J* = 8.2 Hz, 2 H), 6.99 (dd, *J* = 8.3, 2.0 Hz, 1 H), 7.37 (d, *J* = 2.0 Hz, 1 H), 7.62 (m, 2 H), 8.20 (m, 2 H) ppm. ^13^C-NMR (125 MHz, CDCl_3_): δ = 14.3, 17.6, 18.5, 26.0, 27.1, 30.7, 32.5, 32.7, 34.8, 39.1, 39.5, 42.9, 44.9, 57.6, 66.0, 70.3, 81.6, 85.2, 90.9, 115.1, 123.7, 129.5, 130.0, 130.0, 132.5, 138.2, 147.0, 154.3, 170.5, 172.0, 177.6 ppm. HRMS (ESI-ToF) calcd. for C_33_H_41_IN_3_O_8_^+^ [M+H]^+^: 734.1933 found 734.1936.

#### 4.3.11. Synthesis of (3R,9S,11S,13R,14S,16R)-14-Hydroxy-3-(4-hydroxy-3-iodobenzyl)-16-{3-[(2-hydroxyethyl)thio]propyl}-4,9,11,13-tetramethyl-1-oxa-4,7-diazacyclohexadecane-2,5,8-trione (**11a**)

According to the general procedure for thiol-ene click reactions, alkene **8b** (17.6 mg, 28.6 µmol) was dissolved in anhydrous THF (286 µL, 0.1 M) and reacted with 2-mercaptoethanol (5.00 µL, 57.3 µmol, 1.12 gml^−1^, 2.0 equiv), triethylborane (34.4 µL, 8.59 µmol, 0.3 equiv, 0.25 M in hexane) and air (0.4 mL) and stirred for 20 h. The obtained crude product was subjected to reversed-phase chromatography (FlashPure Select C18, H_2_O:MeCN 90:10–MeCN). Thioether **11a** (14.2 mg, 20.5 µmol, 72%) was obtained as colorless powder after lyophilization. ${\left[\alpha \right]}_{D}^{20}$ = –14.9 (c = 1.0, CHCl_3_). ^1^H-NMR (500 MHz, CDCl_3_): δ = 0.83 (d, *J* = 6.7 Hz, 3 H), 0.94 (d, *J* = 6.0 Hz, 3 H), 1.01–1.10 (m, 3 H), 1.12 (d, *J* = 6.6 Hz, 3 H), 1.17 (m, 1 H), 1.35 (ddd, *J* = 13.9, 11.5, 2.0 Hz, 1 H), 1.45 (ddd, *J* = 13.8, 11.6, 2.0 Hz, 1 H), 1.51 (m, 1 H), 1.62 (m, 2 H), 1.72 (m, 2 H), 1.97 (m, 1 H), 2.14–2.35 (m, 2 H), 2.43 (ddq, *J* = 11.7, 6.6, 3.4 Hz, 1 H), 2.55 (m, 2 H), 2.73 (m, 2 H), 2.86 (dd, *J* = 15.3, 12.1 Hz, 1 H), 2.93 (s, 3 H), 3.15 (dd, *J* = 16.7, 2.0 Hz, 1 H), 3.40 (dd, *J* = 15.3, 4.4 Hz, 1 H), 3.63 (ddd, *J* = 11.6, *J* = 4.0, 2.0 Hz, 1 H), 3.74 (t, *J* = 6.0 Hz, 2 H), 4.76 (dd, *J* = 16.7, 8.8 Hz, 1 H), 5.22 (m, 1 H), 5.49 (dd, *J* = 12.1, 4.4 Hz, 1 H), 6.40 (dd, *J* = 8.8, 2.0 Hz, 1 H), 6.83 (d, *J* = 8.2 Hz, 1 H), 7.04 (dd, *J* = 8.2, 1.8 Hz, 1 H), 7.48 (d, *J* = 1.8 Hz, 1 H) ppm. ^13^C-NMR (125 MHz, CDCl_3_): δ = 14.4, 17.6, 18.4, 25.6, 27.0, 30.9, 31.3, 32.6, 33.1, 34.0, 34.4 35.1, 39.1, 39.5, 43.0, 44.8, 58.0, 60.7, 66.0, 72.4 85.2, 115.2, 129.6, 130.2, 138.3, 154.3, 171.0, 171.9, 177.9 ppm. HRMS (ESI-ToF) calcd. for C_29_H_46_IN_2_O_7_^+^ [M+H]^+^: 693.2065 found 693.2076.

#### 4.3.12. Synthesis of (3R,9S,11S,13R,14S,16R)-16-[3-(Butylthio)propyl]-14-hydroxy-3-(4-hydroxy-3-iodobenzyl)-4,9,11,13-tetramethyl-1-oxa-4,7-diazacyclohexadecane-2,5,8-trione (**11b**)

According to the general procedure for thiol-ene click reactions, alkene **8b** (17.9 mg, 29.1 µmol) was dissolved in anhydrous THF (291 µL, 0.1 M) and reacted with 1-butanethiol (6.25 µL, 58.2 µmol, 0.84 gml^−1^, 2.0 equiv), triethylborane (35.0 µL, 8.74 µmol, 0.3 equiv, 0.25 M in hexane) and air (0.4 mL) and stirred for 20 h. The obtained crude product was subjected to reversed-phase chromatography (FlashPure Select C18, H_2_O:MeCN 90:10–MeCN). Thioether **11b** (17.0 mg, 24.1 µmol, 83%) was obtained as colorless powder after lyophilization. ${\left[\alpha \right]}_{D}^{20}$ = −13.2 (c = 1.0, CHCl_3_). ^1^H-NMR (500 MHz, CDCl_3_): δ = 0.83 (d, *J* = 6.9 Hz, 3 H), 0.90–0.97 (m, *J* = 6.7 Hz, 6 H), 1.01–1.10 (m, 3 H), 1.12 (d, *J* = 6.7 Hz, 3 H), 1.19 (m, 1 H), 1.32 (m, 1 H), 1.41 (m, 2 H), 1.45–1.53 (m, 2 H), 1.54–1.63 (m), 1.67 (m, 1 H), 1.76 (m, 1 H), 1.98 (m, 1 H), 2.44 (ddq, *J* = 11.6, 6.7, 3.2 Hz, 1 H), 2.46–2.58 (m, 4 H), 2.86 (dd, *J* = 15.3, 12.3 Hz, 1 H), 2.92 (s, 3 H), 3.22 (d, *J* = 16.7 Hz, 1 H), 3.41 (dd, *J* = 15.4, 4.4 Hz, 1 H), 3.62 (ddd, *J* = 11.4, 4.0, 2.0 Hz, 1 H), 4.77 (dd, *J* = 16.7, 8.6 Hz, 1 H), 5.20 (m, 1 H), 5.49 (dd, *J* = 12.3, 4.4 Hz, 1 H), 6.34 (m, 1 H), 6.83 (m, 1 H), 7.05 (dd, *J* = 8.3, 2.0 Hz, 1 H), 7.48 (d, *J* = 2.0 Hz, 1 H) ppm. ^13^C-NMR (125 MHz, CDCl_3_): δ = 13.7, 14.4, 17.6, 18.4, 22.0, 25.8, 27.0, 30.9, 31.7, 31.8, 32.0, 32.7, 33.5, 34.4, 34.4, 39.1, 39.6, 43.0, 44.9, 58.0, 65.9, 72.6, 85.3, 115.2, 129.5, 130.2, 138.2, 154.3, 171.1, 172.0, 177.7 ppm. HRMS (ESI-ToF) calcd. for C_31_H_50_IN_2_O_6_S^+^ [M+H]^+^: 705.2429 found 705.2426.

#### 4.3.13. Synthesis of (3R,9S,11S,13R,14S,16R)-16-[3-(Benzylthio)propyl]-14-hydroxy-3-(4-hydroxy-3-iodobenzyl)-4,9,11,13-tetramethyl-1-oxa-4,7-diazacyclohexadecane-2,5,8-trione (**11c**)

According to the general procedure for thiol-ene click reactions, alkene **8b** (18.8 mg, 30.6 µmol) was dissolved in anhydrous THF (306 µL, 0.1 M) and reacted with benzyl mercaptan (7.17 µL, 61.2 µmol, 1.06 gml^−1^, 2.0 equiv), triethylborane (36.7 µL, 9.18 µmol, 0.3 equiv, 0.25 M in hexane) and air (0.4 mL) and stirred for 20 h. The obtained crude product was subjected to reversed-phase chromatography (FlashPure Select C18, H_2_O:MeCN 90:10–MeCN). Thioether **11c** (12.5 mg, 16.9 µmol, 55%) was obtained as colorless powder after lyophilization. ${\left[\alpha \right]}_{D}^{20}$ = –6.7 (c = 1.0, CHCl_3_). ^1^H-NMR (500 MHz, CDCl_3_): δ = 0.83 (d, *J* = 6.7 Hz, 3 H), 0.95 (d, *J* = 6.1 Hz, 3 H), 1.02–1.09 (m, 3 H), 1.13 (d, *J* = 6.7 Hz, 3 H), 1.17 (m, 1 H), 1.29 (m, 1 H), 1.43 (m, 1 H), 1.48–1.59 (m, 3 H), 1.63 (m, 1 H), 1.71 (m, 1 H), 1.98 (m, 1 H), 2.39–2.47 (m, 3 H), 2.84 (dd, *J* = 15.4, 12.2 Hz, 1 H), 2.88 (s, 3 H), 3.22 (d, *J* = 16.6 Hz, 1 H), 3.38 (dd, *J* = 15.4, 4.4 Hz, 1 H), 3.60 (ddd, *J* = 11.4, 4.2, 2.0 Hz, 1 H), 3.71 (s, 2 H), 4.76 (dd, *J* = 16.6, 8.7 Hz, 1 H), 5.16 (m, 1 H), 5.46 (dd, *J* = 12.2, 4.4 Hz, 1 H), 6.29 (d, *J* = 8.7 Hz, 1 H), 6.85 (d, *J* = 8.2 Hz, 1 H), 7.04 (dd, *J* = 8.2, 2.0 Hz, 1 H), 7.24 (m, 1 H), 7.29–7.34 (m, 4 H), 7.47 (d, *J* = 2.0 Hz, 1 H) ppm. ^13^C-NMR (125 MHz, CDCl_3_): δ = 14.4, 17.6, 18.4, 25.4, 27.0, 30.8, 31.0, 32.6, 33.5, 34.4, 34.4, 36.5, 39.1, 39.6, 43.0, 44.9, 58.0, 65.8, 72.6, 85.4, 115.2, 127.0, 128.5, 128.8, 129.6, 130.3, 138.1, 138.4, 154.2, 171.2, 171.9, 177.7 ppm. HRMS (ESI-ToF) calcd. for C_34_H_48_IN_2_O_6_S^+^ [M+H]^+^: 739.2272 found 739.2298.

#### 4.3.14. Synthesis of Ethyl 3-({3-[(3R,9S,11S,13R,14S,16R)-14-hydroxy-3-(4-hydroxy-3-iodobenzyl)-4,9,11,13-tetramethyl-2,5,8-trioxo-1-oxa-4,7-diazacyclohexadecan-16-yl]propyl}-thio)propanoate (**11d**)

According to the general procedure for thiol-ene click reactions, alkene **8b** (18.8 mg, 30.6 µmol) was dissolved in anhydrous THF (306 µL, 0.1 M) and reacted with ethyl 3-mercaptopropanoate (7.75 µL, 61.2 µmol, 1.06 gml^−1^, 2.0 equiv), triethylborane (36.7 µL, 9.18 µmol, 0.3 equiv, 0.25 M in hexane) and air (0.4 mL). After stirring for 2 h, triethylborane (36.7 µL, 9.18 µmol, 0.3 equiv, 0.25 M in hexane) was added and stirred for another 24 h. The obtained crude product was subjected to reversed-phase chromatography (FlashPure Select C18, H_2_O:MeCN 90:10–MeCN) and further purified by preparative HPLC (Luna C18, H_2_O:MeCN 25:75–MeCN). Thioether **11d** (19.2 mg, 25.6 µmol, 79%) was obtained as colorless powder after lyophilization. ${\left[\alpha \right]}_{D}^{20}$ = –8.5 (c = 1.0, CHCl_3_). ^1^H-NMR (500 MHz, CDCl_3_): δ = 0.83 (d, *J* = 6.7 Hz, 3 H), 0.95 (d, *J* = 6.0 Hz, 3 H), 1.00–1.09 (m, 3 H), 1.12 (d, *J* = 6.6 Hz, 3 H), 1.19 (m, 1 H), 1.27 (t, *J* = 7.2 Hz, 3 H), 1.33 (m, 1 H), 1.43–1.55 (m, 2 H), 1.56–1.71 (m, 3 H), 1.76 (m, 1 H), 1.85 (bs, 1 H), 1.98 (m, 1 H), 2.43 (m, 1 H), 2.55 (m, 2 H), 2.60 (t, *J* = 7.4 Hz, 2 H), 2.78 (t, *J* = 7.4 Hz, 2 H), 2.88 (dd, *J* = 15.3, 11.9 Hz, 1 H), 2.93 (s, 3 H), 3.22 (d, *J* = 16.5 Hz, 1 H), 3.41 (dd, *J* = 15.3, 4.0 Hz, 1 H), 3.62 (m, 1 H), 4.16 (q, *J* = 7.2 Hz, 2 H), 4.77 (dd, *J* = 16.5, 7.7 Hz, 1 H), 5.21 (m, 1 H), 5.48 (dd, *J* = 12.0, 4.2 Hz, 1 H), 6.33 (bs, 1 H), 6.84 (d, *J* = 8.2 Hz, 1 H), 7.06 (d, *J* = 7.9 Hz, 1 H), 7.50 (s, 1 H) ppm. ^13^C-NMR (125 MHz, CDCl_3_): δ = 14.2, 14.4, 17.6, 18.4, 25.6, 27.0, 27.0, 30.9, 31.7, 32.6, 33.5, 34.3, 34.4, 34.9, 39.1, 39.6, 43.0, 44.9, 58.1, 60.7, 65.9, 72.5, 85.2, 115.2, 129.6, 130.3, 138.2, 154.3, 171.2, 171.9, 172.0, 177.7 ppm. HRMS (ESI-ToF) calcd. for C_32_H_50_IN_2_O_8_S^+^ [M+H]^+^: 749.2327 found 749.2360.

#### 4.3.15. Synthesis of 2-({3-[(3R,9S,11S,13R,14S,16R)-14-Hydroxy-3-(4-hydroxy-3-iodobenzyl)-4,9,11,13-tetramethyl-2,5,8-trioxo-1-oxa-4,7-diazacyclohexadecan-16-yl]propyl}-thio)acetic Acid (**11e**)

According to the general procedure for thiol-ene click reactions, alkene **8b** (19.1 mg, 31.1 µmol) was dissolved in anhydrous THF (296 µL, 0.1 M) and reacted with 2-mercaptoacetic acid (4.31 µL, 62.2 µmol, 1.33 gml^−1^, 2.0 equiv), triethylborane (37.3 µL, 9.32 µmol, 0.3 equiv, 0.25 M in hexane) and air (0.4 mL). After stirring for 2 h, triethylborane (37.3 µL, 9.32 µmol, 0.3 equiv, 0.25 M in hexane) was added and stirred for another 18 h. The obtained crude product was subjected to reversed-phase chromatography (FlashPure Select C18, H_2_O:MeCN 90:10–MeCN) and further purified by preparative HPLC (Luna C18, H_2_O:MeCN 25:75–MeCN). Thioether **11e** (14.4 mg, 20.4 µmol, 66%) was obtained as colorless powder after lyophilization. ${\left[\alpha \right]}_{D}^{20}$ = −11.5 (c = 1.0, CHCl_3_). ^1^H-NMR (500 MHz, CDCl_3_): δ = 0.84 (d, *J* = 6.7 Hz, 3 H), 0.94 (d, *J* = 6.0 Hz, 3 H), 1.03–1.11 (m, 3 H), 1.11–1.18 (m, *J* = 6.6 Hz, 4 H), 1.36 (m, 1 H), 1.44 (m, 1 H), 1.56 (m, 1 H), 1.60–1.77 (m, 3 H), 1.82 (m, 1 H), 1.99 (m, 1 H), 2.46 (ddq, *J* = 12.1, 6.6, 3.4 Hz, 1 H), 2.62 (m, 2 H), 2.86 (dd, *J* = 15.5, 12.1 Hz, 1 H), 2.98 (s, 3 H), 3.16 (d, *J* = 16.8 Hz, 1 H), 3.23 (s, 2 H), 3.40 (dd, *J* = 15.4, 4.4 Hz, 1 H), 3.65 (ddd, *J* = 11.3, 4.1, 2.1 Hz, 1 H), 4.96 (dd, *J* = 16.8, 9.0 Hz, 1 H), 5.27 (m, 1 H), 5.55 (dd, *J* = 12.1, 4.4 Hz, 1 H), 6.72 (bs, 1 H), 6.84 (d, *J* = 8.2 Hz, 1 H), 7.04 (d, *J* = 8.4, 1.8 Hz, 1 H), 7.48 (d, *J* = 2.0 Hz, 1 H) ppm. ^13^C-NMR (125 MHz, CDCl_3_): δ = 14.2, 17.5, 18.3, 25.0, 26.9, 30.9, 31.9, 32.4, 33.4, 33.5, 34.3, 34.6, 39.2, 39.7, 42.9, 44.6, 57.7, 66.0, 72.1, 85.3, 115.2, 129.6, 130.3, 138.2, 154.3, 170.8, 171.8, 173.4, 178.5 ppm. HRMS (ESI-ToF) calcd. for C_29_H_44_IN_2_O_6_S^+^ [M+H]^+^: 707.1858 found 707.1884.

#### 4.3.16. Synthesis of S-{3-[(3R,9S,11S,13R,14S,16R)-14-Hydroxy-3-(4-hydroxy-3-iodobenzyl)-4,9,11,13-tetramethyl-2,5,8-trioxo-1-oxa-4,7-diazacyclohexadecan-16-yl]propyl} ethanethioate (**11f**)

According to the general procedure for thiol-ene click reactions, alkene **8b** (18.0 mg, 29.3 µmol) was dissolved in anhydrous THF (293 µL, 0.1 M) and reacted with thioacetic acid (4.17 µL, 58.6 µmol, 1.07 gml^−1^, 2.0 equiv), triethylborane (35.1 µL, 8.79 µmol, 0.3 equiv, 0.25 M in hexane) and air (0.4 mL) and stirred for 20 h. The obtained crude product was subjected to reversed-phase chromatography (FlashPure Select C18, H_2_O:MeCN 90:10–MeCN). Thioether **11f** (19.0 mg, 27.5 µmol, 94%) was obtained as colorless powder after lyophilization. ${\left[\alpha \right]}_{D}^{20}$ = −11.6 (c = 1.0, CHCl_3_). ^1^H-NMR (500 MHz, CDCl_3_): δ = 0.83 (d, *J* = 6.7 Hz, 3 H), 0.95 (d, *J* = 6.1 Hz, 3 H), 1.00–1.09 (m, 3 H), 1.12 (d, *J* = 6.6 Hz, 3 H), 1.18 (m, 1 H), 1.32 (m, 1 H), 1.45 (m, 1 H), 1.52 (m, 1 H), 1.56–1.66 (m, 3 H), 1.72 (m, 1 H), 1.99 (m, 1 H), 2.34 (s, 3 H), 2.44 (m, 1 H), 2.85 (t, *J* = 6.8 Hz, 2 H), 2.89 (m, 1 H), 2.93 (s, 3 H), 3.22 (d, *J* = 16.6 Hz, 1 H), 3.40 (dd, *J* = 15.4, 4.4 Hz, 1 H), 3.61 (ddd, *J* = 11.3, 4.0, 1.9 Hz, 1 H), 4.77 (dd, *J* = 16.8, 8.7 Hz, 1 H), 5.20 (m, 1 H), 5.48 (dd, *J* = 12.1, 4.3 Hz, 1 H), 6.33 (bs, 1 H), 6.74 (bs, 1 H), 6.83 (d, *J* = 8.4 Hz, 1 H), 7.06 (dd, *J* = 8.4, 1.8 Hz, 1 H), 7.49 (d, *J* = 1.8 Hz, 1 H) ppm. ^13^C-NMR (125 MHz, CDCl_3_): δ = 14.4, 17.6, 18.4, 25.9, 27.0, 28.7, 30.7, 30.9, 32.6, 33.6, 34.3, 34.4, 39.1, 39.6, 43.0, 44.9, 58.1, 65.8, 72.4, 85.3, 115.2, 129.6, 130.3, 138.2, 154.2, 171.2, 171.9, 177.7, 195.9 ppm. HRMS (ESI-ToF) calcd. for C_29_H_44_IN_2_O_7_S^+^ [M+H]^+^: 691.1908 found 691.1909.

### 4.4. Cytotoxicity Evaluation

The cell cultures were cultivated at 37 °C under an atmosphere containing 5% CO_2_. Before usage, Dulbecco’s Modified Eagle Medium (DMEM) from Gibco (Thermo Fisher Scientific) was supplemented with 10% fetal bovine serum (FBS) from Gibco. Cells were used between passage 5 and 30 and were split separately for at least two passages to obtain biological repeats. Cells were washed with PBS and 0.5 mL trypsin was added before treatment. Cells were then incubated for 5 min before adding 10 mL medium containing 10% FBS. Per well, 120 µL cell suspension 5 × 10^4^ cells/mL (CHO-K1, HCT-116. U-2 OS, KB3.1) or 1 × 10^5^ cells/mL (HepG2) cells were seeded in transparent 96-well cell bind plate and incubated for 2 h at 37 °C and an atmosphere of 5% CO_2_. Each compound was tested in a serial dilution so that the starting concentration is 111 µg/mL, which is diluted by 1:3 as well as the internal solvent control prepared in DMEM with 10% FBS. After 5 d incubation at 37 °C and an atmosphere of 5% CO_2_, 20 μL of 5 mg/mL MTT (thiazolyl blue tetrazolium bromide) in PBS was added per well and cells were further incubated until coloration of the cells. The medium was then discarded and 100 μL 2-propanol/10 N HCl (250:1) added to the cells. The plates were analyzed by measuring the absorbance at 570 nm and 630 nm as reference using a microplate reader Infinite^®^ 200 Pro from Tecan (Männedorf, Switzerland). Absorption is then converted to cell viability expressed as percentage relative to the respective solvent control. The calculated percentage of growth inhibition were determined by sigmoidal curve fitting using GraphPad (Boston, MA, USA) Prism software (version 10.0.2). Two independent measurements were generated for mean and standard deviation.

## 5. Conclusions

We have successfully synthesized a variety of doliculide derivates with focus on late-stage modification at the terminal position of the polyketide fragment. The incorporation of an alkene as well as an alkyne moiety during the last step of the Matteson homologation allowed us to accomplish modifications via cycloadditions, Sonogashira couplings, and thiol-ene click reactions. The more polar derivatives generally led to a decreased activity. Apart from one synthesized derivative, all modifications at the *i*-Pr moiety presented herein led to inactivity against HepG2.

## Figures and Tables

**Table 1:** IC_50_ values of doliculide **A** and derivatives **8**–**11** towards different cancer cell lines ^[a]^.

Entry	Compound	IC_50_				
		HepG2	CHO-K1	HCT-116	U-2 OS	KB3.1
1	**A**	16.4 ± 4.2 nM	110.8 ± 4.5 nM	8.8 ± 0.5 nM	25.2 ± 1.8 nM	38.0 ± 9.9 nM
2	**8a**	20.0 ± 4.5 µM	380 ± 44 nM	8.0 ± 7.3 nM	336 ± 146 nM	≤115 nM ^[b]^
3	**8b**	>60.2 µM	264 ± 13 nM	16 ± 8 nM	521 ± 488 nM	103 ± 65 nM
4	**8c**	147 ± 65 nM	474 ± 147 nM	65 ± 16 nM	49 ± 33 nM	114 ± 49 nM
5	**9a**	>49.6 µM	24 ± 9 nM	309 ± 81 nM	≤ 80 nM ^[b]^	47 ± 13 nM
6	**9b**	>50.1 µM	69 ± 12 nM	≤ 110 nM ^[b]^	≤ 110 nM ^[b]^	41 ± 14 nM
7	**9c**	>46.0 µM	23.8 ± 0.5 µM	3.81 ± 1.00 µM	14.4 ± 1.3 µM	21.9 ± 12.6 µM
8	**9d**	>46.2 µM	>46.2 µM	>46.2 µM	>46.2 µM	>46.2 µM
9	**9e**	>47.0 µM	>47.0 µM	8.55 ± 1.14 µM	23.0 ± 4.4 µM	10.5 ± 0.3 µM
10	**9f**	>41.7 µM	6.38 ± 2.81 µM	86 ± 79 nM	1.56 ± 0.67 µM	722 ± 180 nM
11	**9g**	>39.7 µM	12.9 ± 3.0 µM	432 ± 140 nM	2.94 ± 1.75 µM	3.29 ± 1.39 µM
12	**10a**	15.5 ± 0.3 µM	247 ± 145 nM	14.5 ± 0.6 nM	683 ± 87 nM	49 ± 29 nM
13	**10b**	>51.5 µM	1.46 ± 0.28 µM	153 ± 56 nM	1.88 ± 0.11 µM	459 ± 111 µM
14	**10c**	>50.4 µM	586 ± 205 nM	10 ± 3 nM	491 ± 95 nM	98 ± 41 nM
15	**11a**	>53.4 µM	765 ± 13 nM	22 ± 9 nM	129 ± 14 nM	196 ± 72 nM
16	**11b**	>52.5 µM	142 ± 85 nM	>52.5 µM	238 ± 10 nM	28 ± 3 nM
17	**11c**	>50.0 µM	391 ± 122 nM	10.8 ± 1.2 nM	582 ± 68 nM	37 ± 1 nM
18	**11d**	>49.4 µM	9.84 ± 3.87 µM	60 ± 13 nM	4.90 ± 3.87 µM	3.93 ± 1.14 µM
19	**11e**	>52.3 µM	39.8 ± 18.0 µM	6.21 ± 3.00 µM	>52.3 µM	>52.3 µM
20	**11f**	>53.6 µM	6.08 ± 2.32 µM	41 ± 23 nM	6.21 ± 1.74 µM	>53.6 µM
Color code		<10.0 nM	10.0–100.0 nM	100.0 nM–1.0 µM	1.0–10.0 µM	>10.0 µM

^[a]^ HepG2: human hepatocellular carcinoma; CHO-K1: mutagenized Chinese hamster ovary; HCT-116: human colon carcinoma; U-2 OS: human bone osteosarcoma; KB3.1: human epidermoid carcinoma cell line. ^[b]^ The individual results varied widely and were therefore given as a range.

## Data Availability

The authors confirm that the data supporting the findings of this study are available within the article or the Supplementary Materials.

## Supplementary Materials

The following supporting information can be downloaded at: https://www.mdpi.com/article/10.3390/md22040165/s1, ^1^H and ^13^C NMR spectra of compounds **2**–**11**.
