# Supplementary material for: Synthesis and Late-Stage Modification of (−)-Doliculide Derivatives Using Matteson’s Homologation Approach
Source: Mar Drugs. 2024 Apr 8;22(4):165. doi: 10.3390/md22040165 (PMC11051198; doi:10.3390/md22040165)

Supporting Materials

Synthesis and Late-Stage Modification of (–)-Doliculide Derivatives Using Matteson's Homologation approach

Markus Tost [1] and Uli Kazmaier [1,*]

Organic Chemistry, Saarland University, Campus Building C4.2, D-66123 Saarbruecken, Germany;
* Correspondence: u.kazmaier@mx.uni-saarland.de; Tel.: +49-681-302-3409

Copies of the NMR spectra

$^{1}H/^{13}C$-NMR Compound **2a** S2
$^{1}H/^{13}C$-NMR Compound **2b** S3
$^{1}H/^{13}C$-NMR Compound **3a** S4
$^{1}H/^{13}C$-NMR Compound **3b** S5
$^{1}H/^{13}C$-NMR Compound **5a** S6
$^{1}H/^{13}C$-NMR Compound **5b** S8
$^{1}H/^{13}C$-NMR Compound **5a-1** S10
$^{1}H/^{13}C$-NMR Compound **5b-1** S11
$^{1}H/^{13}C$-NMR Compound **6a** S12
$^{1}H/^{13}C$-NMR Compound **6b** S13
$^{1}H/^{13}C$-NMR Compound **7a** S14
$^{1}H/^{13}C$-NMR Compound **7b** S15
$^{1}H/^{13}C$-NMR Compound **7a-1** S16
$^{1}H/^{13}C$-NMR Compound **7b-1** S17
$^{1}H/^{13}C$-NMR Compound **8a** S18
$^{1}H/^{13}C$-NMR Compound **8b** S19
$^{1}H/^{13}C$-NMR Compound **8c** S20
$^{1}H/^{13}C$-NMR Compound **9a** S21
$^{1}H/^{13}C$-NMR Compound **9b** S22
$^{1}H/^{13}C$-NMR Compound **9c** S23
$^{1}H/^{13}C$-NMR Compound **9d** S24
$^{1}H/^{13}C$-NMR Compound **9e** S25
$^{1}H/^{13}C$-NMR Compound **9f** S26
$^{1}H/^{13}C$-NMR Compound **9g** S27
$^{1}H/^{13}C$-NMR Compound **10a** S28
$^{1}H/^{13}C$-NMR Compound **10b** S29
$^{1}H/^{13}C$-NMR Compound **10c** S30
$^{1}H/^{13}C$-NMR Compound **11a** S31
$^{1}H/^{13}C$-NMR Compound **11b** S32
$^{1}H/^{13}C$-NMR Compound **11c** S33
$^{1}H/^{13}C$-NMR Compound **11d** S34
$^{1}H/^{13}C$-NMR Compound **11e** S35
$^{1}H/^{13}C$-NMR Compound **11f** S36

Copies of NMR spectra

{(4*R*,6*S*,7*R*,9*R*,11*S*)-4-[(4*S*,5*S*)-4,5-Dicyclohexyl-1,3,2-dioxaborolan-2-yl]-6-[(4-methoxybenzyl)oxy]-7,9,11-trimethyl-12-(trityloxy)dodec-1-yn-1-yl}trimethylsilane (2a)

^{1}H-NMR (400 MHz, $CDCl_3$)

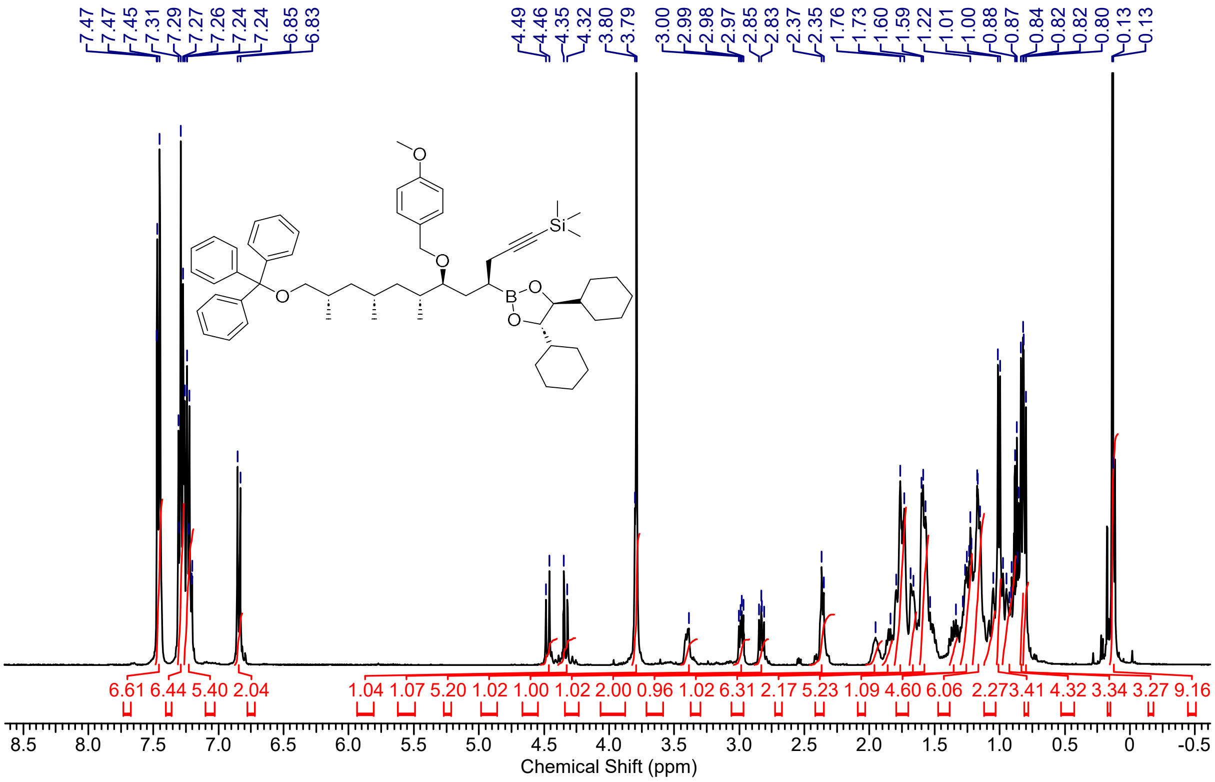

^{13}C-NMR (100 MHz, $CDCl_3$)

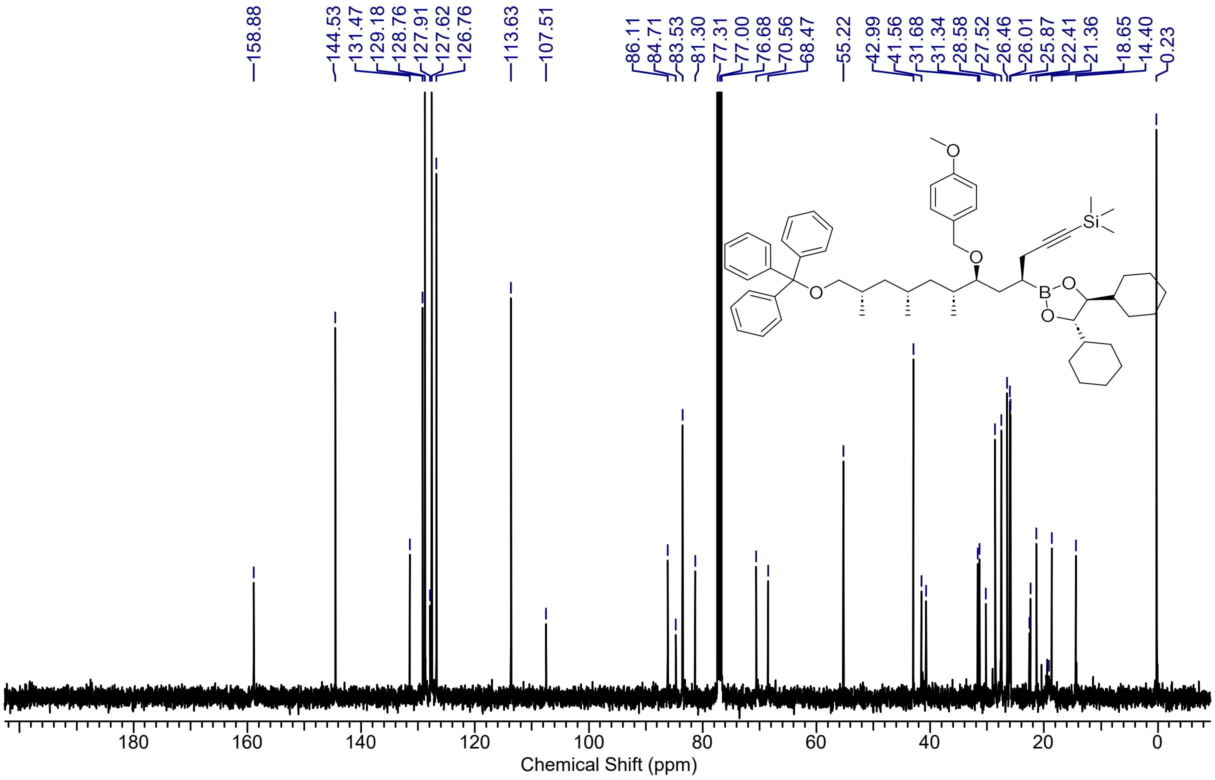

(4*S*,5*S*)-4,5-Dicyclohexyl-2-{(4*R*,6*S*,7*R*,9*R*,11*S*)-6-[(4-methoxybenzyl)oxy]-7,9,11-trimethyl-12-(trityloxy)dodec-1-en-4-yl}-1,3,2-dioxaborolane (2b)

^{1}H-NMR (400 MHz, $CDCl_3$)

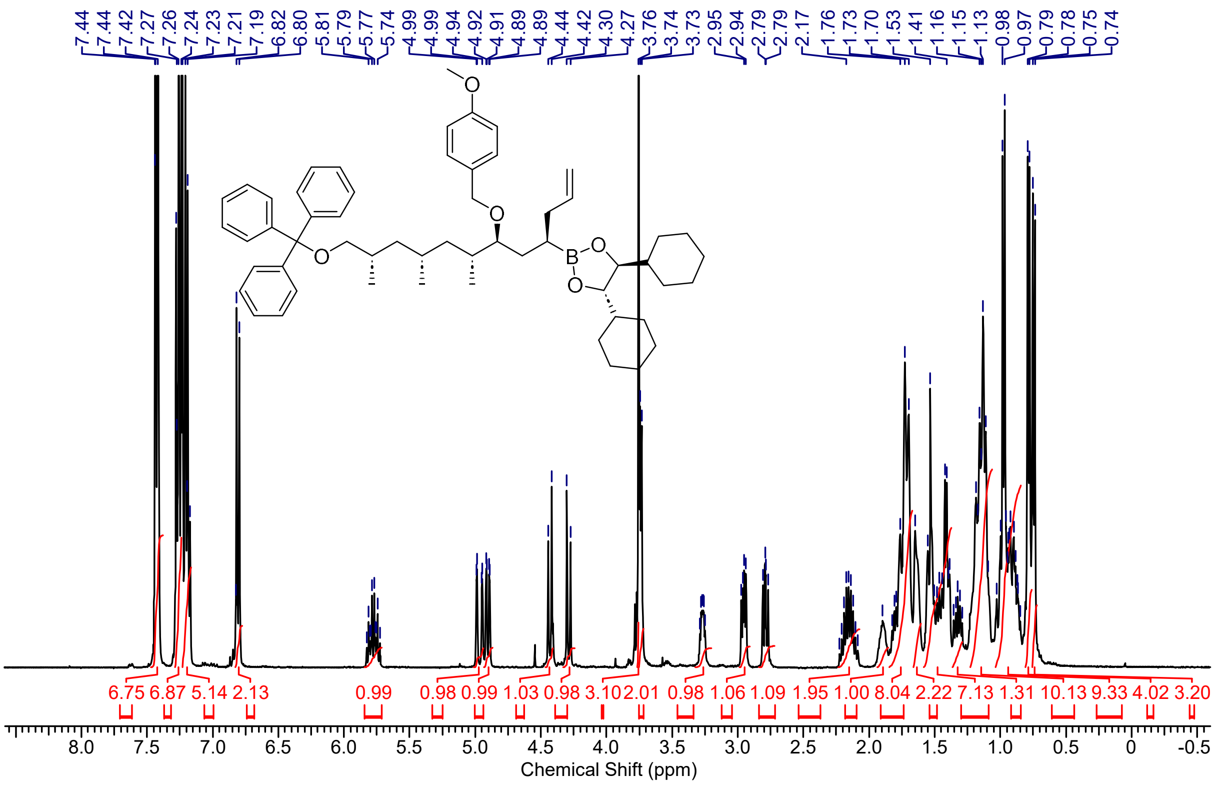

^{13}C-NMR (100 MHz, $CDCl_3$)

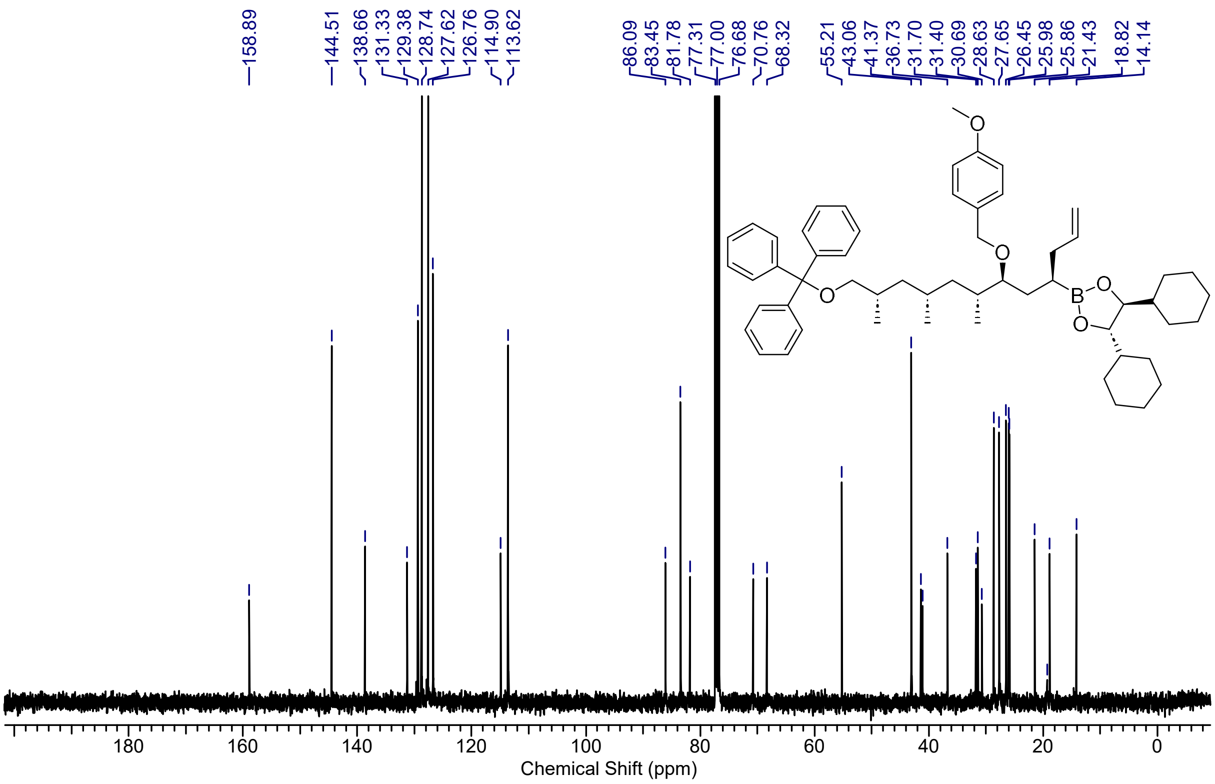

(4*R*,6*S*,7*R*,9*R*,11*S*)-6-[(4-Methoxybenzyl)oxy]-7,9,11-trimethyl-1-(trimethylsilyl)-12-(trityloxy)dodec-1-yn-4-ol (3a)

^{1}H-NMR (400 MHz, $CDCl_3$)

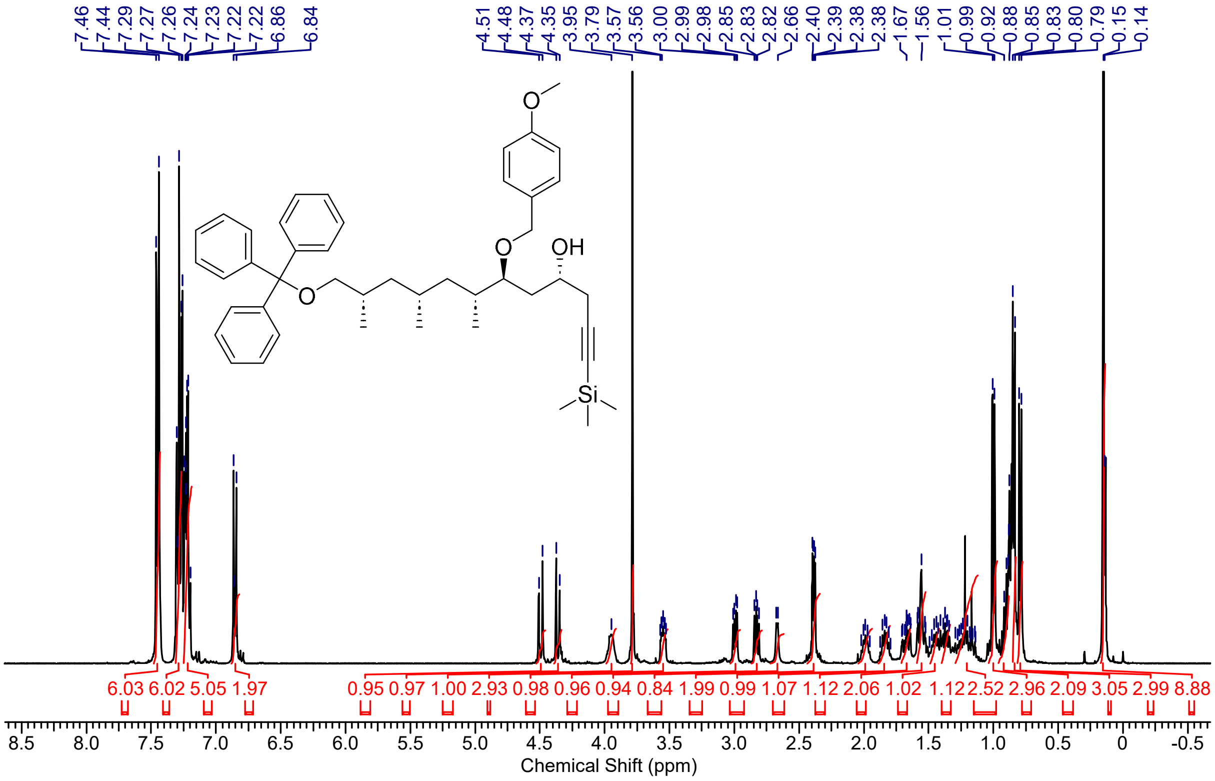

^{13}C-NMR (100 MHz, $CDCl_3$)

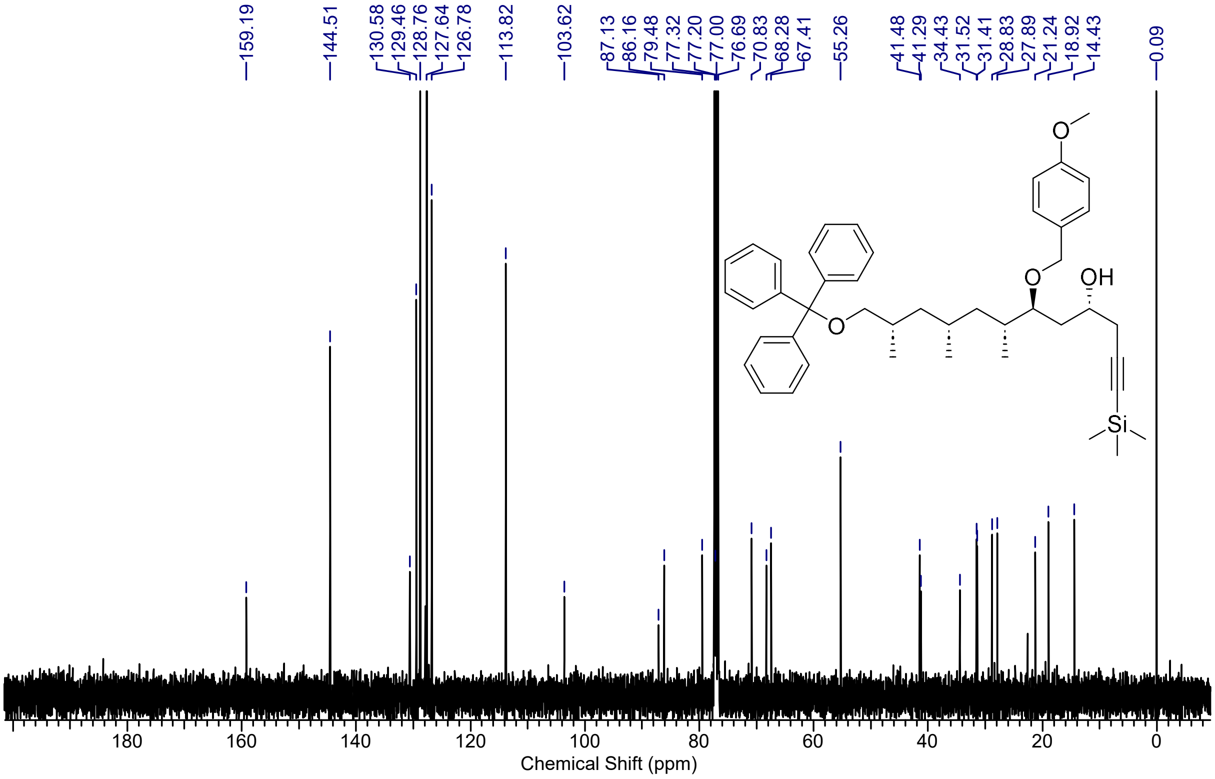

(4*R*,6*S*,7*R*,9*R*,11*S*)-6-[(4-Methoxybenzyl)oxy]-7,9,11-trimethyl-12-(trityloxy)dodec-1-en-4-ol (3b)

^{1}H-NMR (400 MHz, $CDCl_3$)

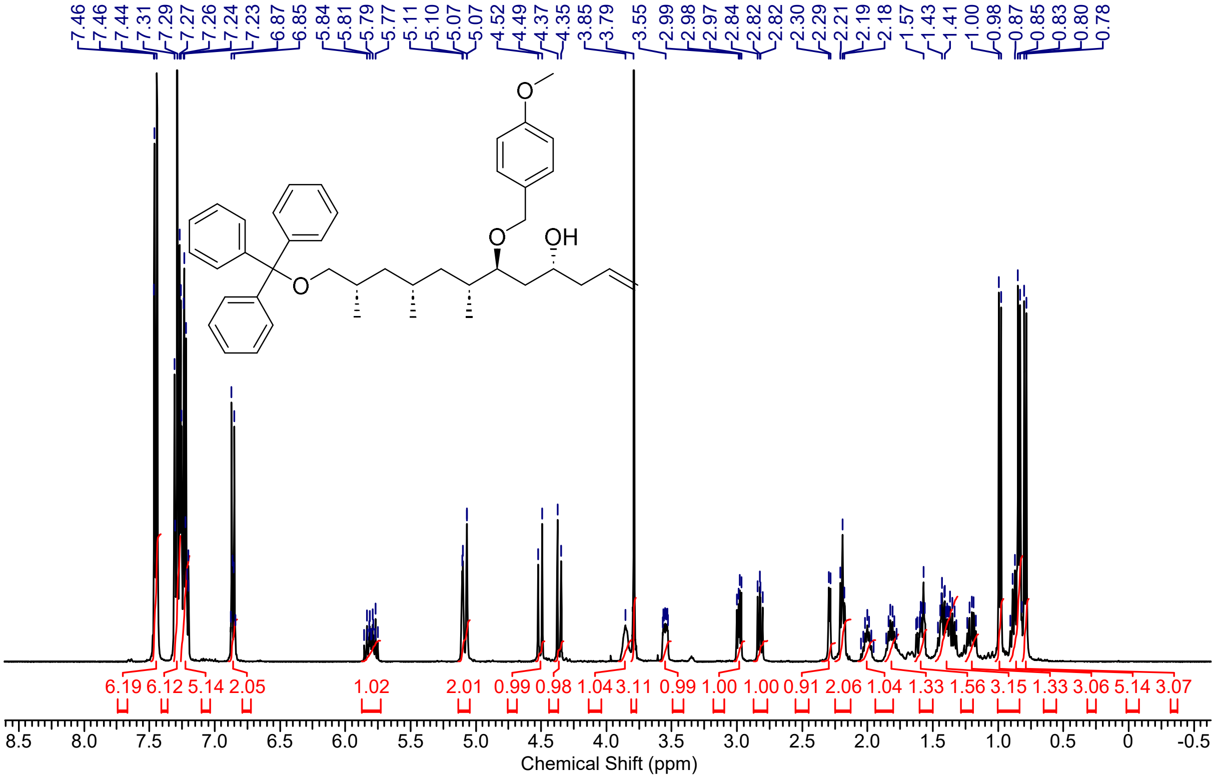

^{13}C-NMR (100 MHz, $CDCl_3$)

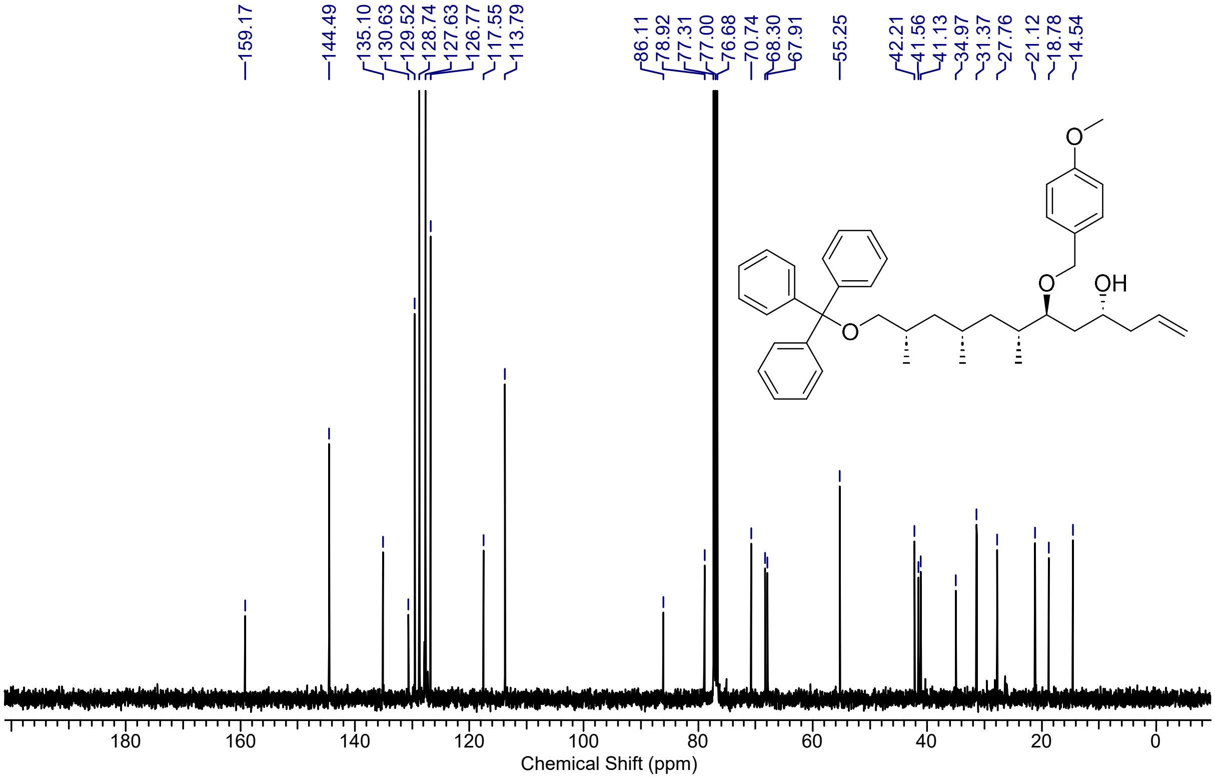

(4*R*,6*S*,7*R*,9*R*,11*S*)-6-[(4-Methoxybenzyl)oxy]-7,9,11-trimethyl-1-(trimethylsilyl)-12-(trityloxy)dodec-1-yn-4-yl (*R*)-3-[4-(allyloxy)-3-iodophenyl]-2-[(*tert*-butoxycarbonyl)(methyl)amino]propanoate (5a)

^{1}H-NMR (500 MHz, 373 K, DMSO-D$_6$)

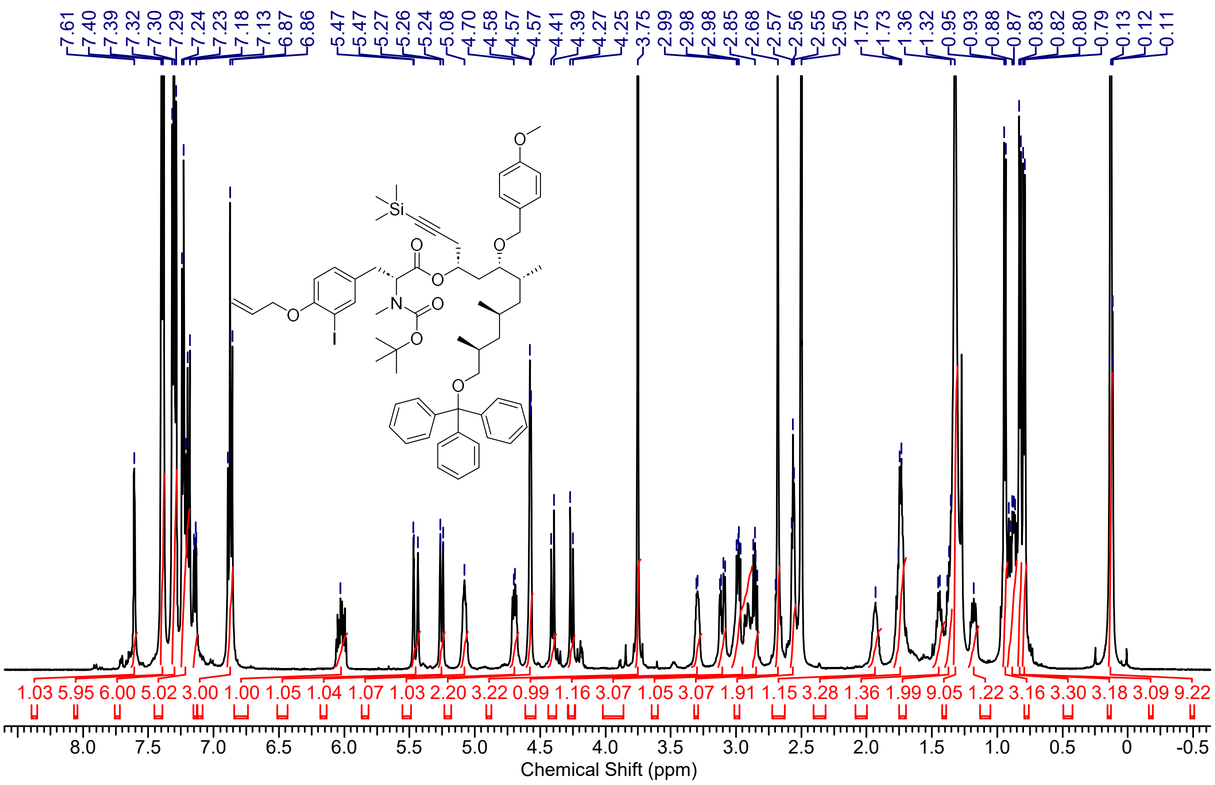

^{13}C-NMR (125 MHz, 373 K, DMSO-D$_6$)

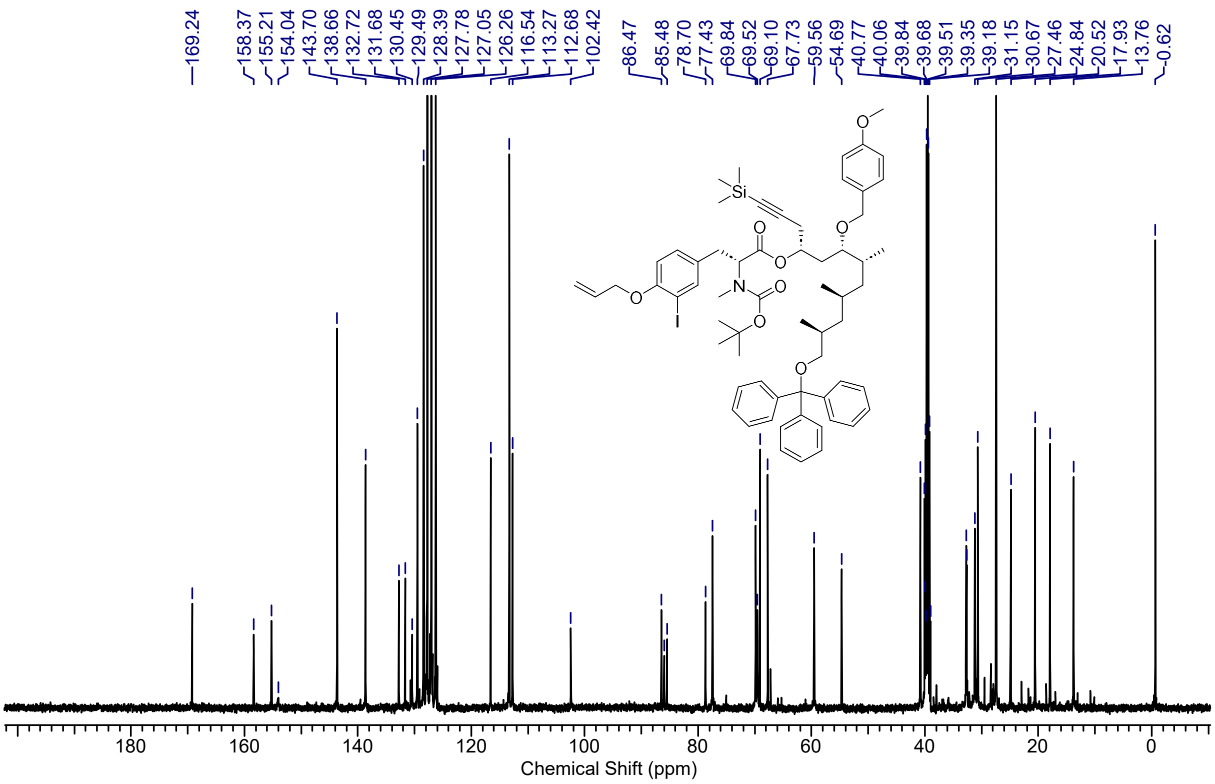

^{1}H-NMR (500 MHz, $CDCl_3$)

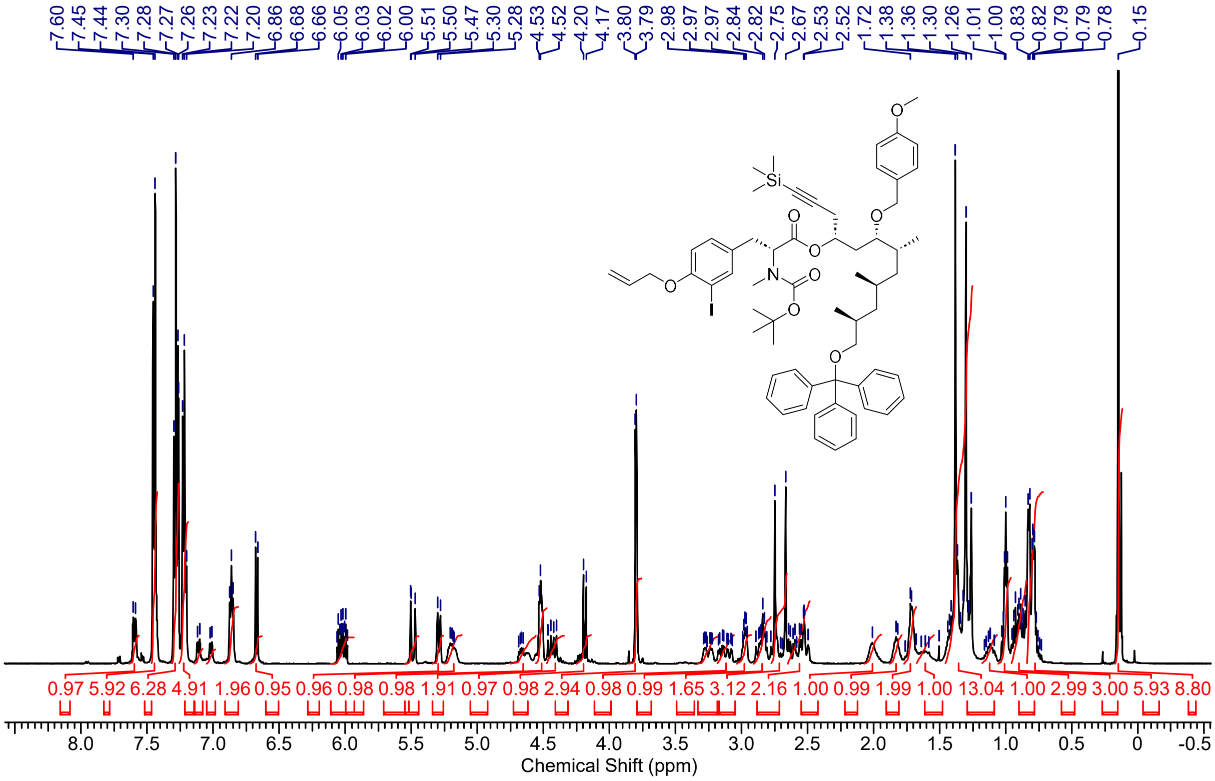

^{13}C-NMR (125 MHz, $CDCl_3$)

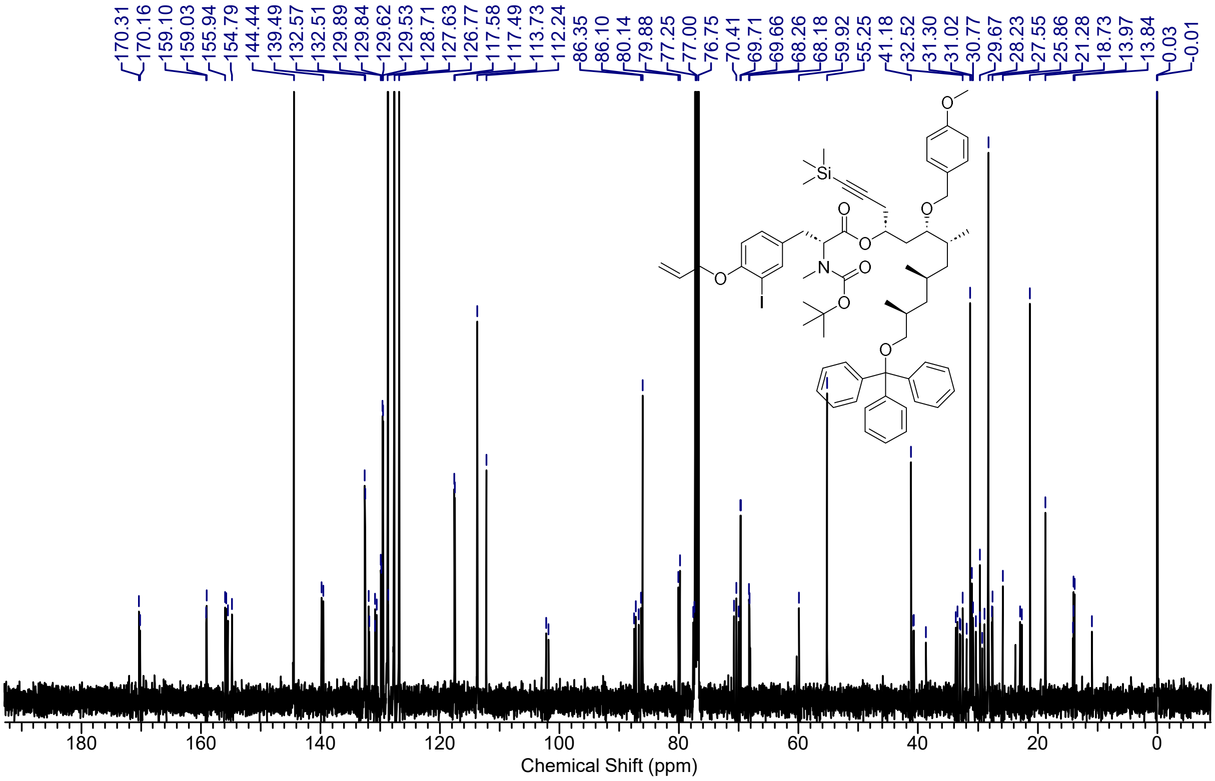

(4*R*,6*S*,7*R*,9*R*,11*S*)-6-[(4-Methoxybenzyl)oxy]-7,9,11-trimethyl-12-(trityloxy)dodec-1-en-4-yl (*R*)-3-[4-(allyloxy)-3-iodophenyl]-2-[(*tert*-butoxycarbonyl)(methyl)amino]propanoate (5b)

^{1}H-NMR (500 MHz, 373 K, DMSO-D$_6$)

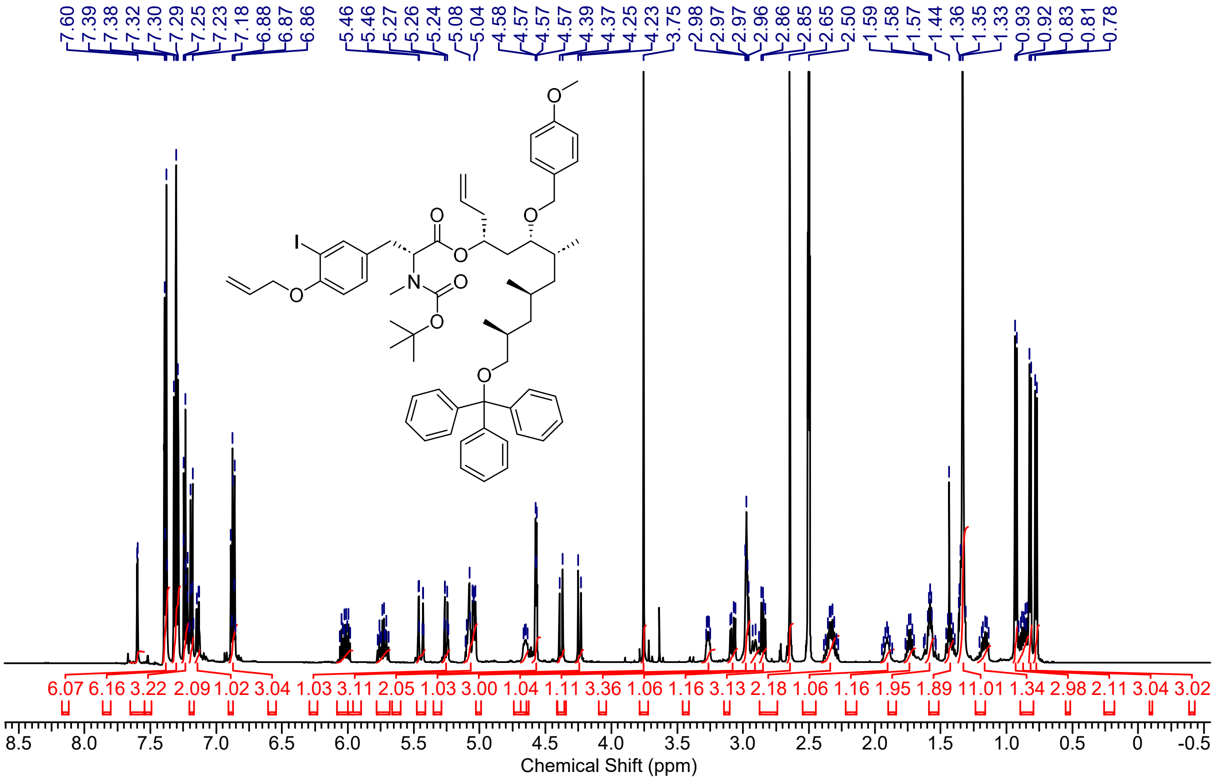

^{13}C-NMR (125 MHz, 373 K, DMSO-D$_6$)

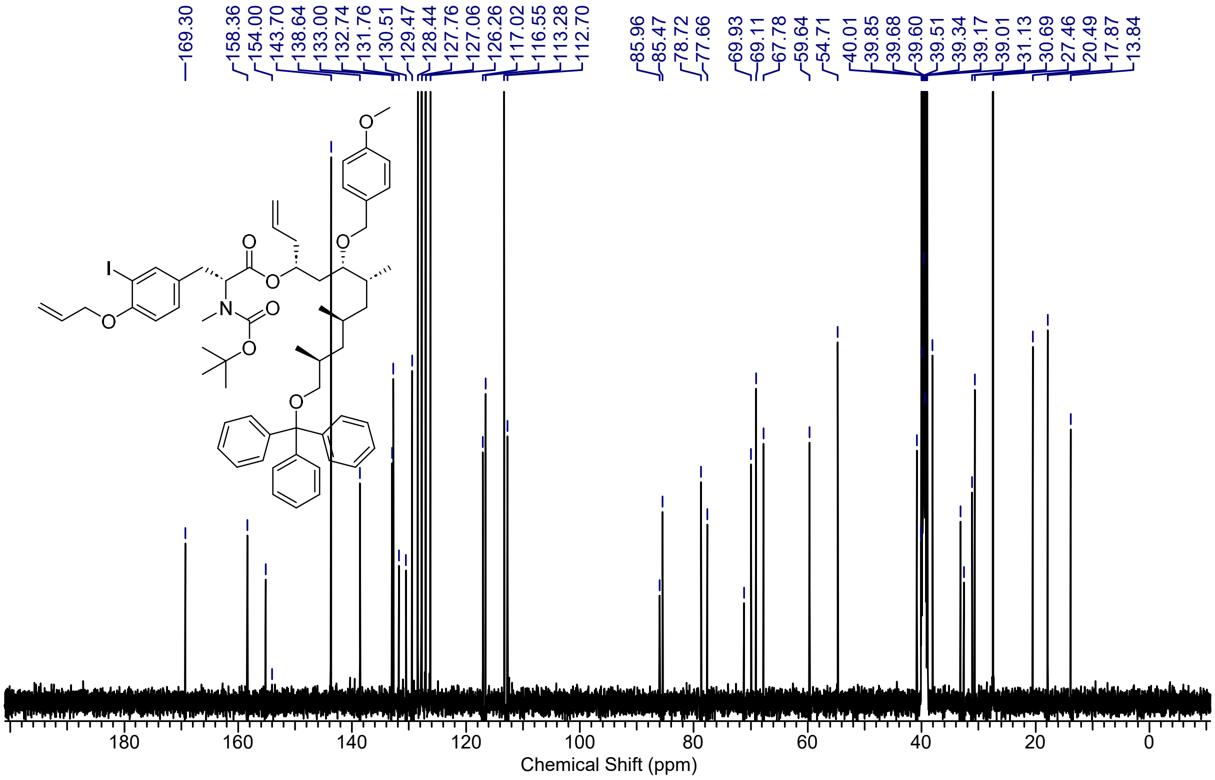

^{1}H-NMR (400 MHz, $CDCl_3$)

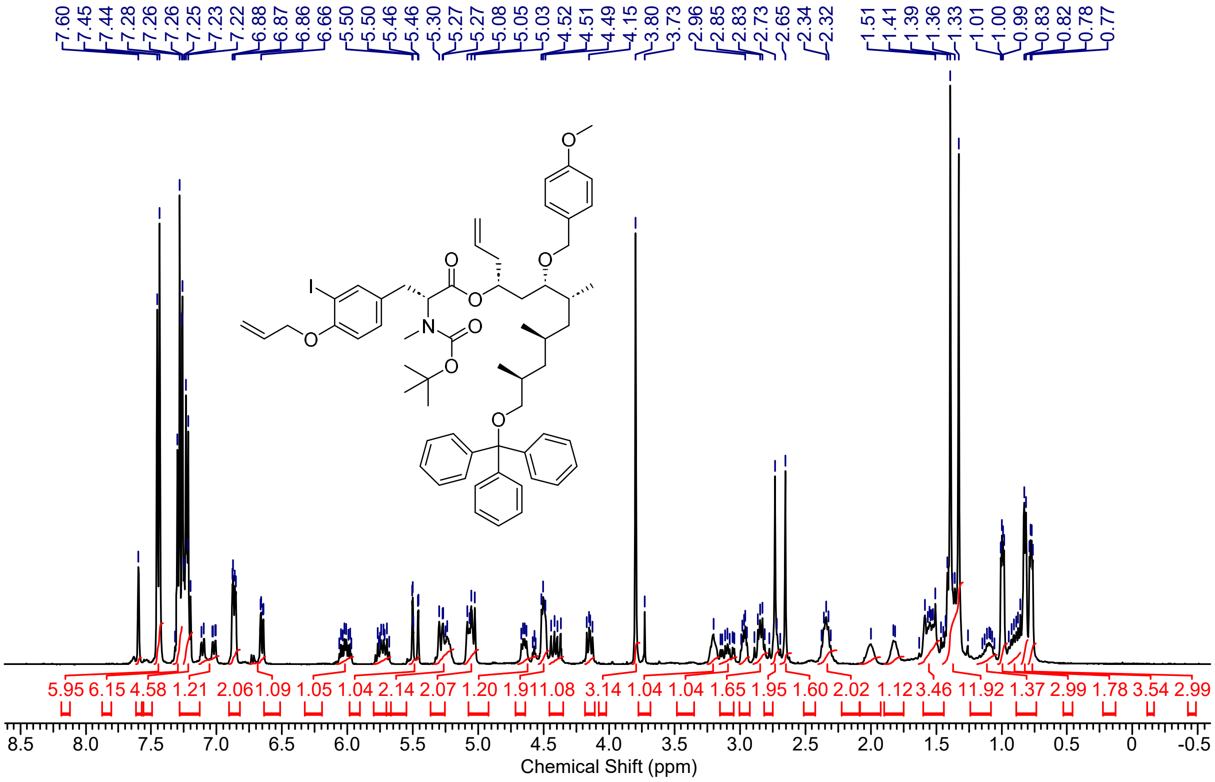

^{13}C-NMR (100 MHz, $CDCl_3$)

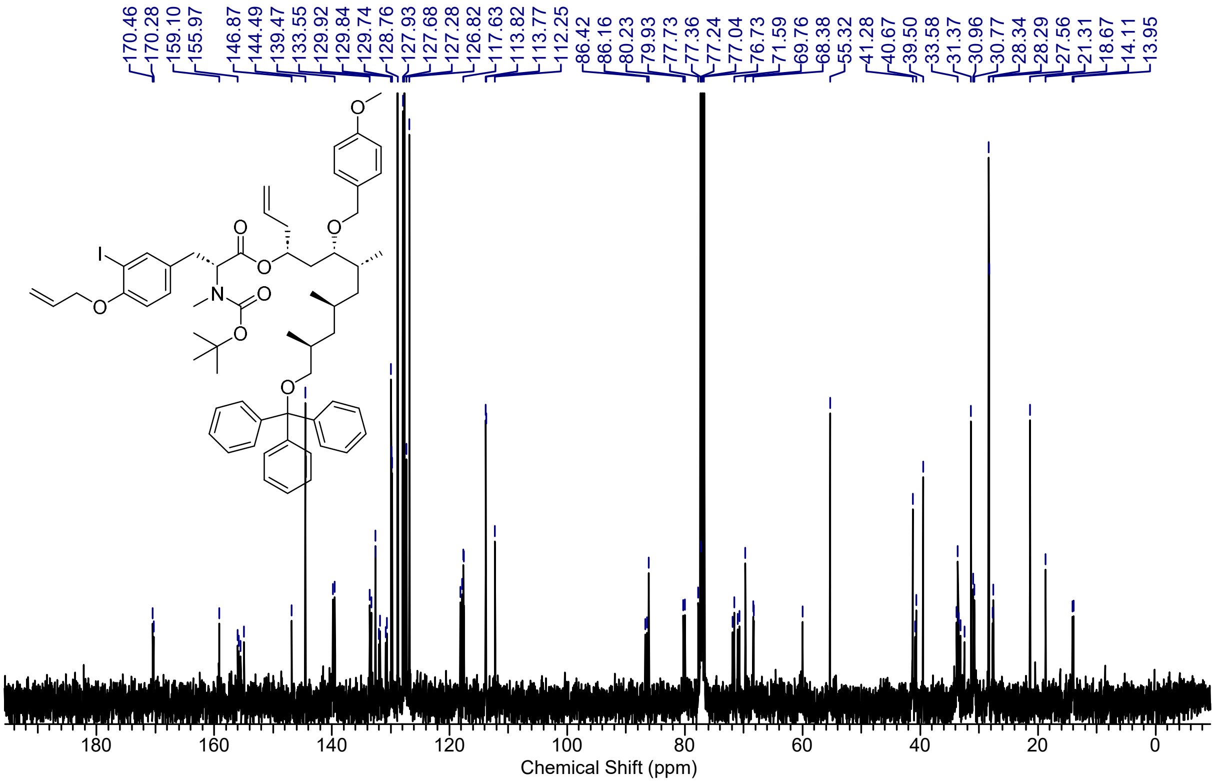

(4*R*,6*S*,7*R*,9*R*,11*S*)-12-Hydroxy-6-[(4-methoxybenzyl)oxy]-7,9,11-trimethyl-1-(trimethylsilyl)dodec-1-yn-4-yl (*R*)-3-[4-(allyloxy)-3-iodophenyl]-2-[(*tert*-butoxycarbonyl)(methyl)amino]propanoate (5a-1)

^{1}H-NMR (500 MHz, 373 K, DMSO-D$_6$)

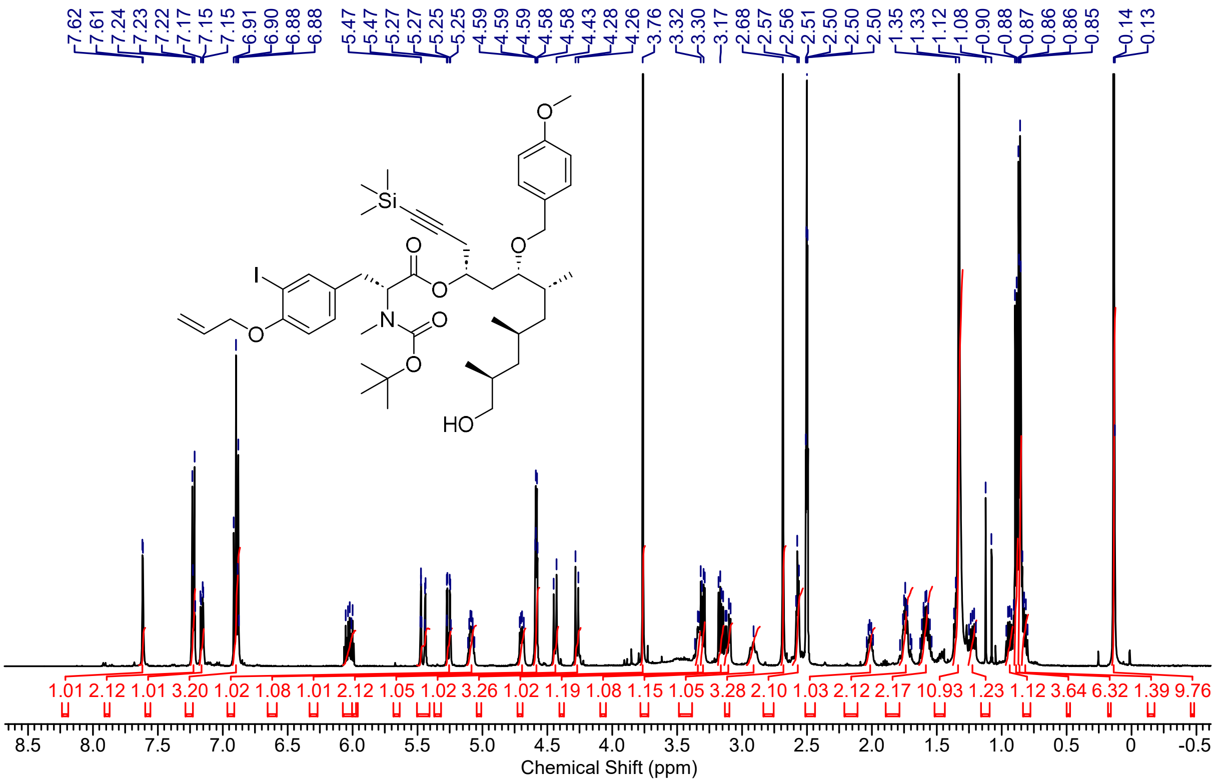

^{13}C-NMR (125 MHz, 373 K, DMSO-D$_6$)

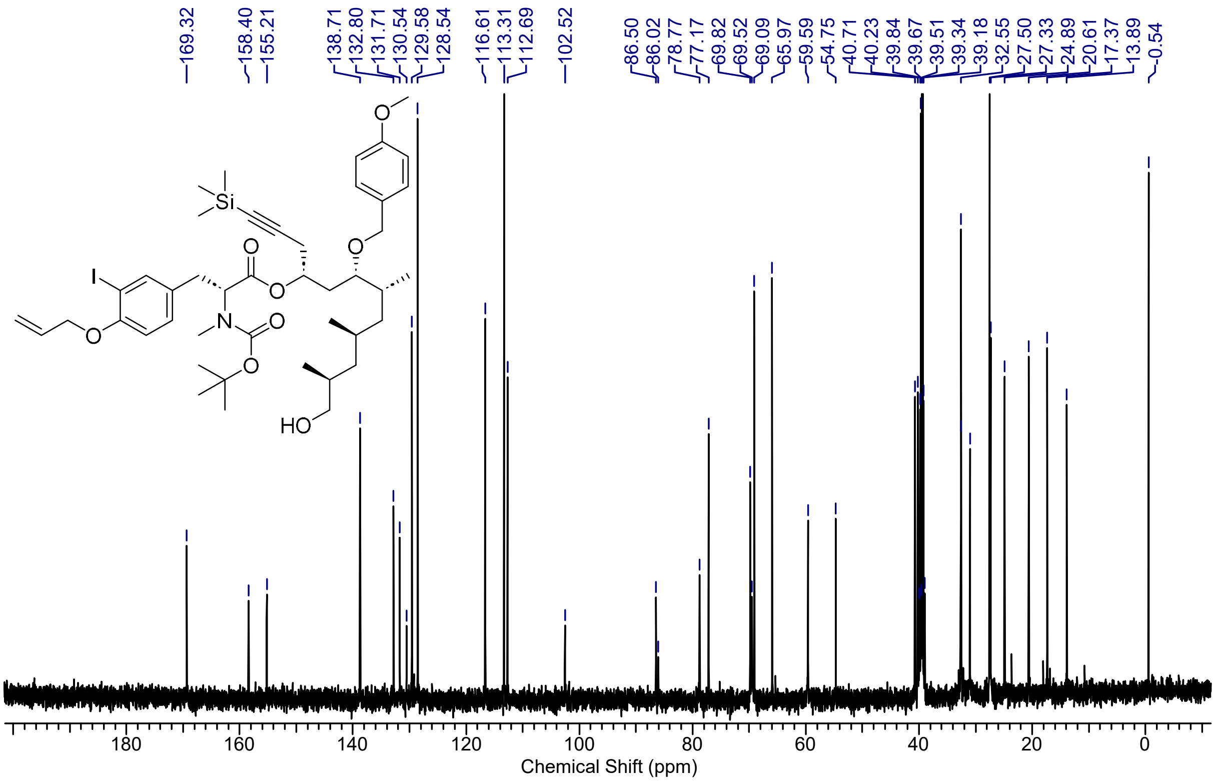

(4*R*,6*S*,7*R*,9*R*,11*S*)-12-Hydroxy-6-[(4-methoxybenzyl)oxy]-7,9,11-trimethyldodec-1-en-4-yl (*R*)-3-[4-(allyloxy)-3-iodophenyl]-2-[(*tert*-butoxycarbonyl)(methyl)amino]propanoate (5b-1)

^{1}H-NMR (500 MHz, 373 K, DMSO-D$_6$)

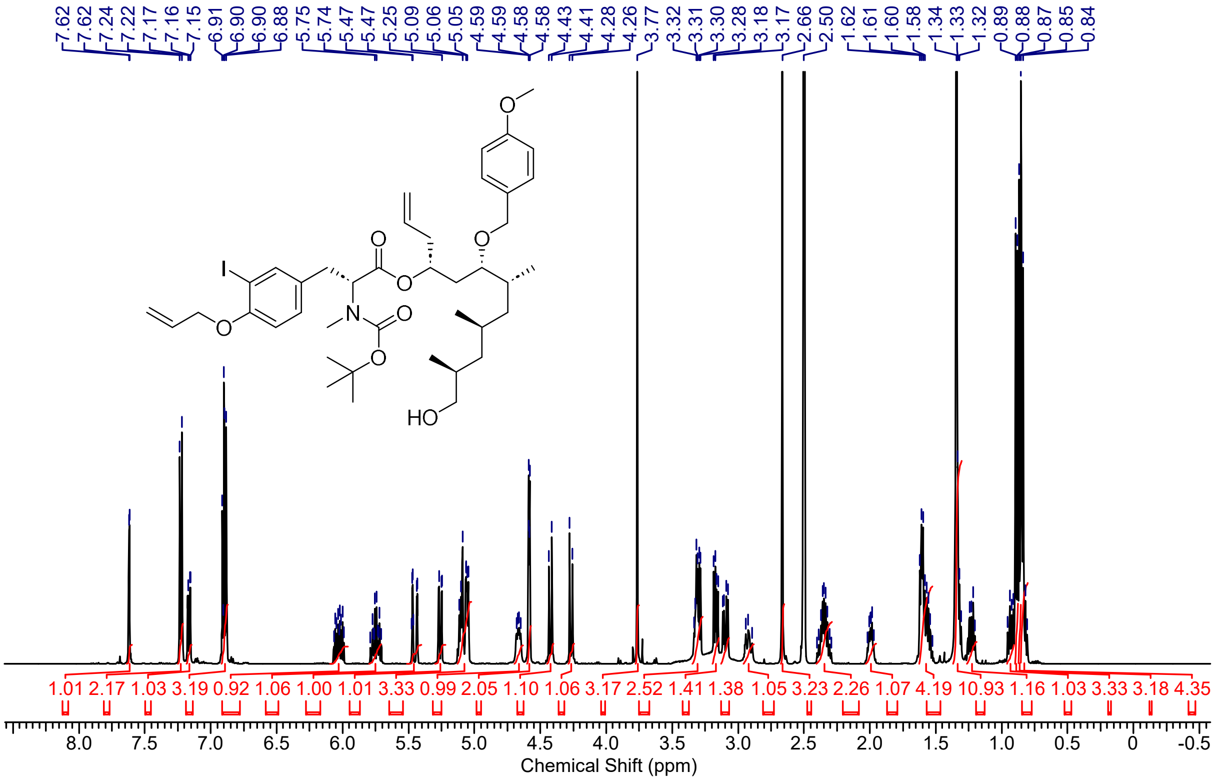

^{13}C-NMR (125 MHz, 373 K, DMSO-D$_6$)

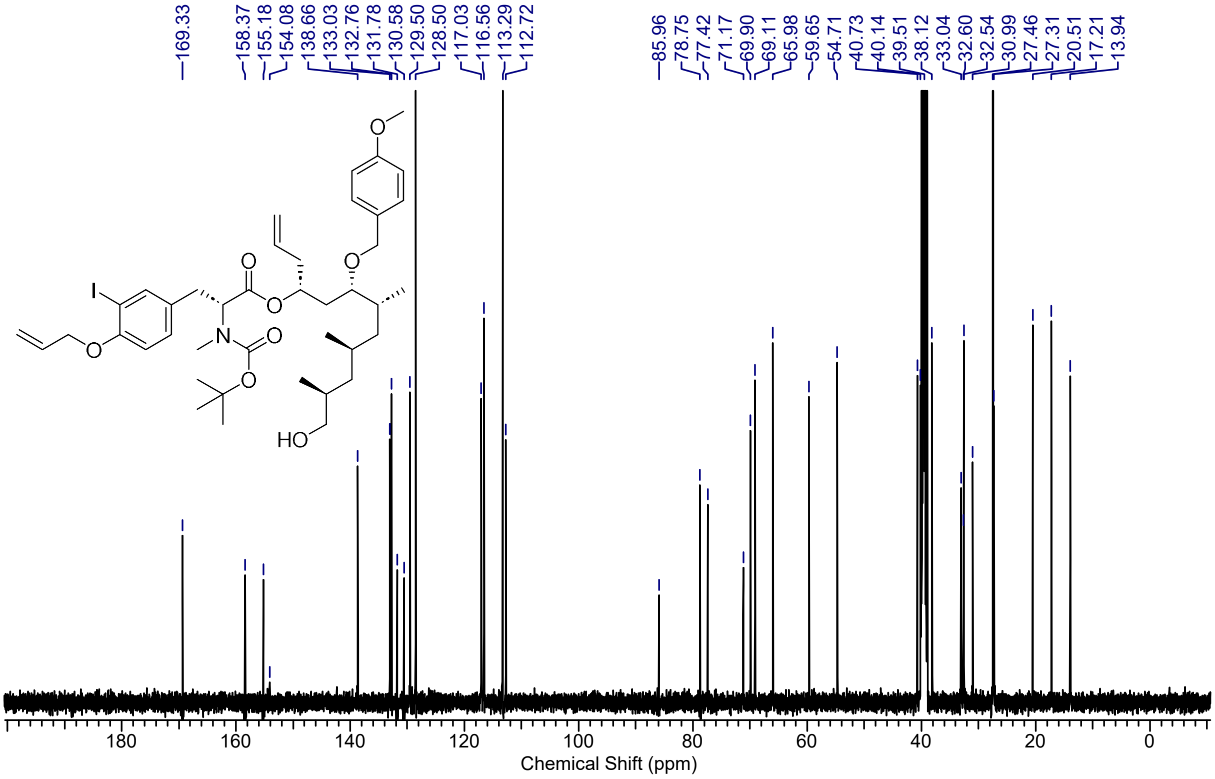

(2*S*,4*S*,6*R*,7*S*,9*R*)-9-({(*R*)-3-[4-(Allyloxy)-3-iodophenyl]-2-[(*tert*-butoxycarbonyl)(methyl)amino]-propanoyl}oxy)-7-[(4-methoxybenzyl)oxy]-2,4,6-trimethyl-12-(trimethylsilyl)dodec-11-ynoic acid (6a)

^{1}H-NMR (500 MHz, 373 K, DMSO-D$_6$)

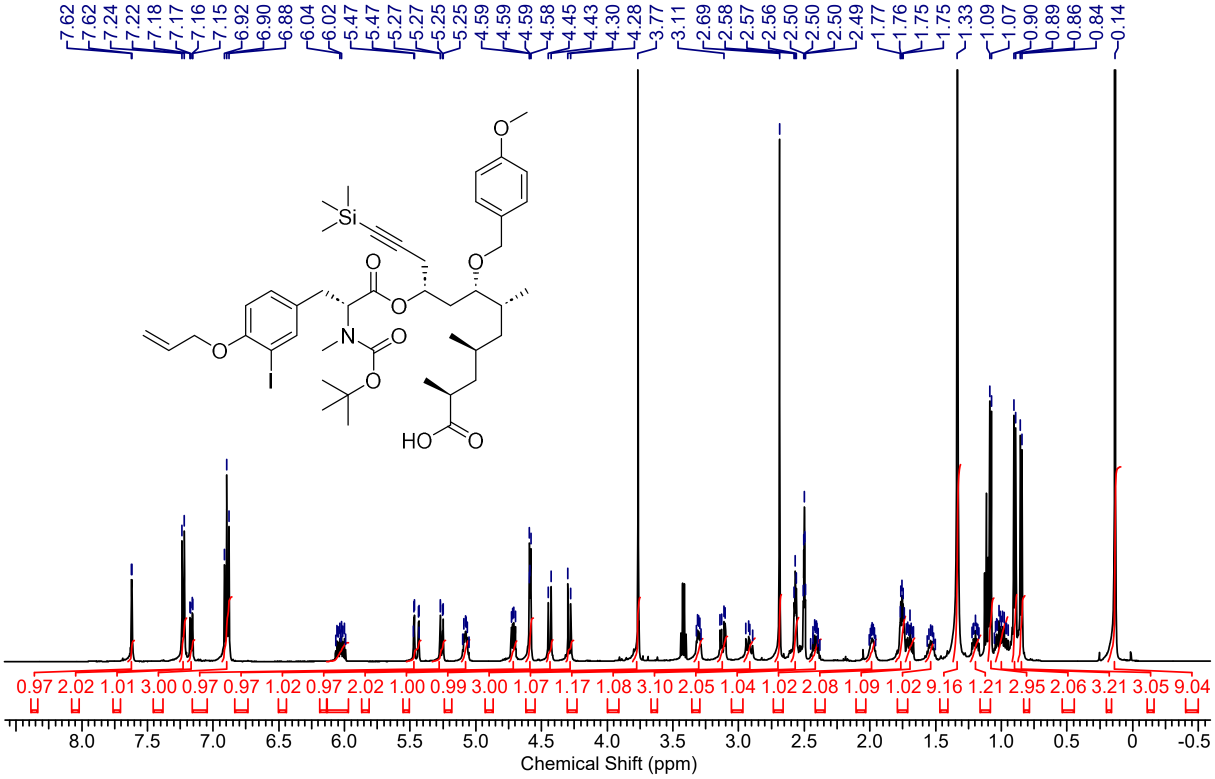

^{13}C-NMR (125 MHz, 373 K, DMSO-D$_6$)

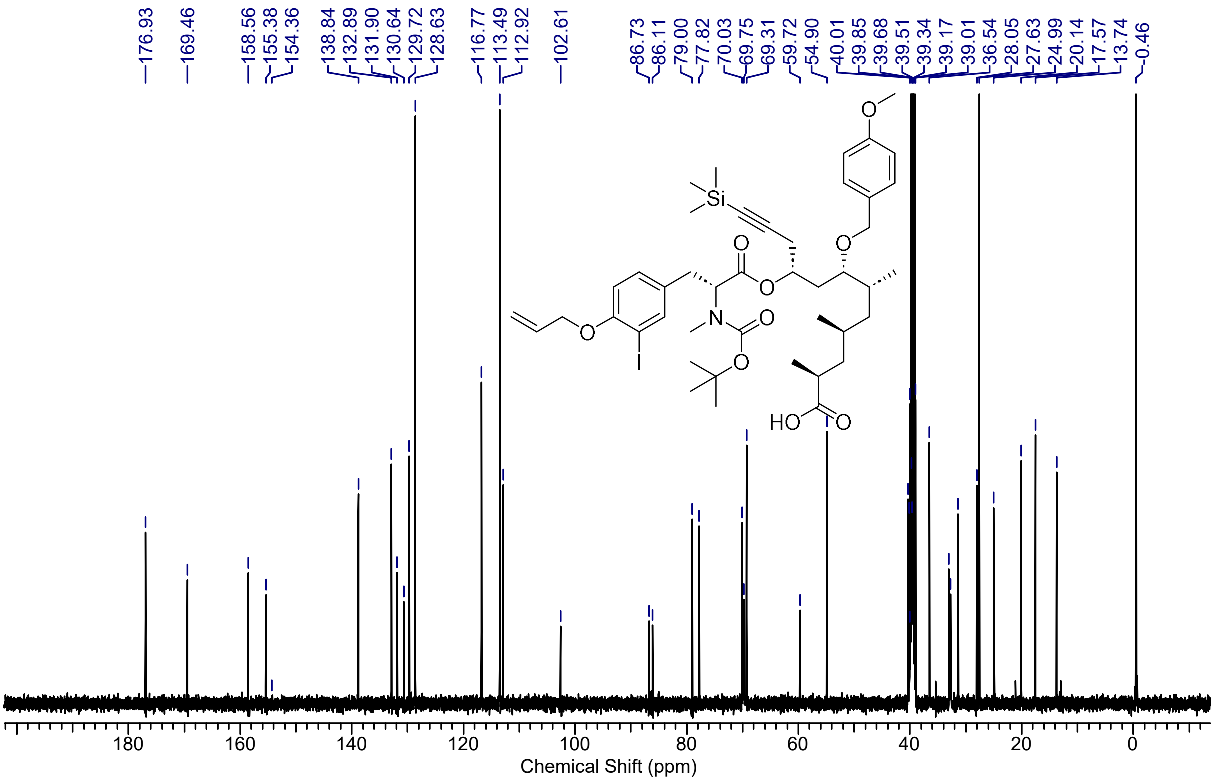

(2*S*,4*S*,6*R*,7*S*,9*R*)-9-({(*R*)-3-[4-(Allyloxy)-3-iodophenyl]-2-[(*tert*-butoxycarbonyl)(methyl)amino]-propanoyl}oxy)-7-[(4-methoxybenzyl)oxy]-2,4,6-trimethyldodec-11-enoic acid (6b)

^{1}H-NMR (500 MHz, 373 K, DMSO-D$_6$)

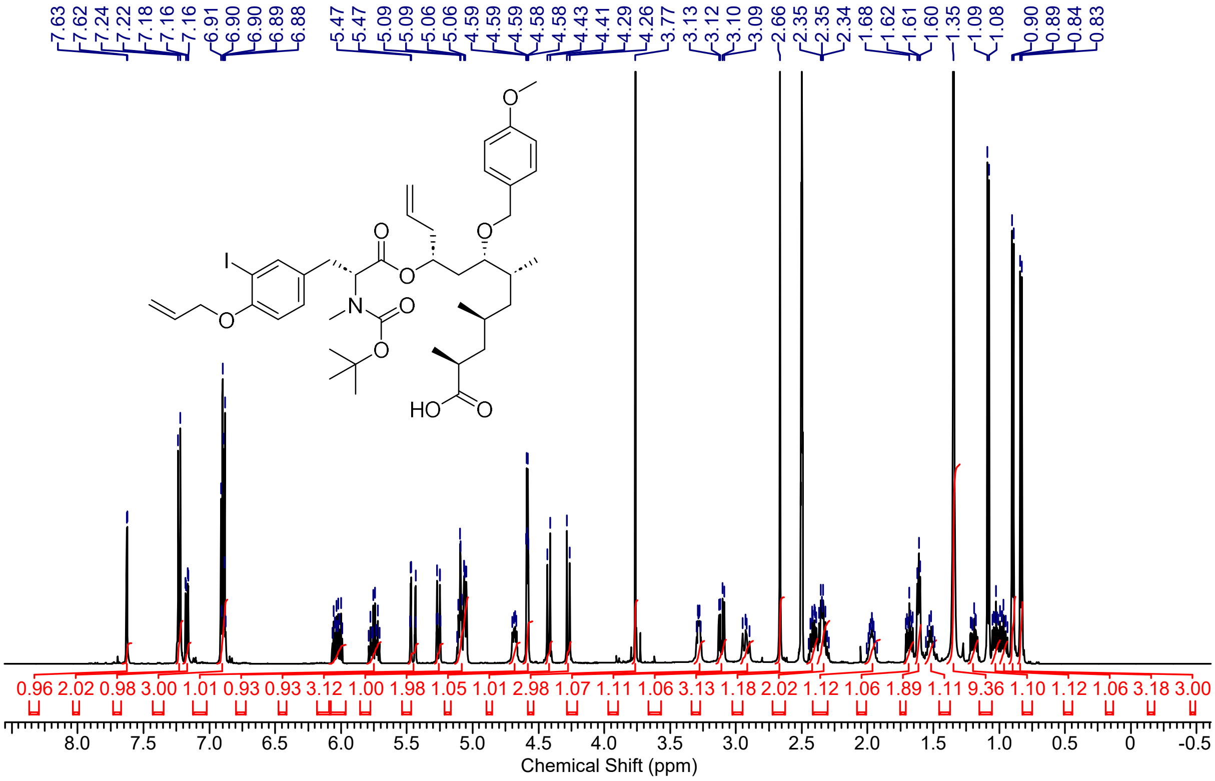

^{13}C-NMR (125 MHz, 373 K, DMSO-D$_6$)

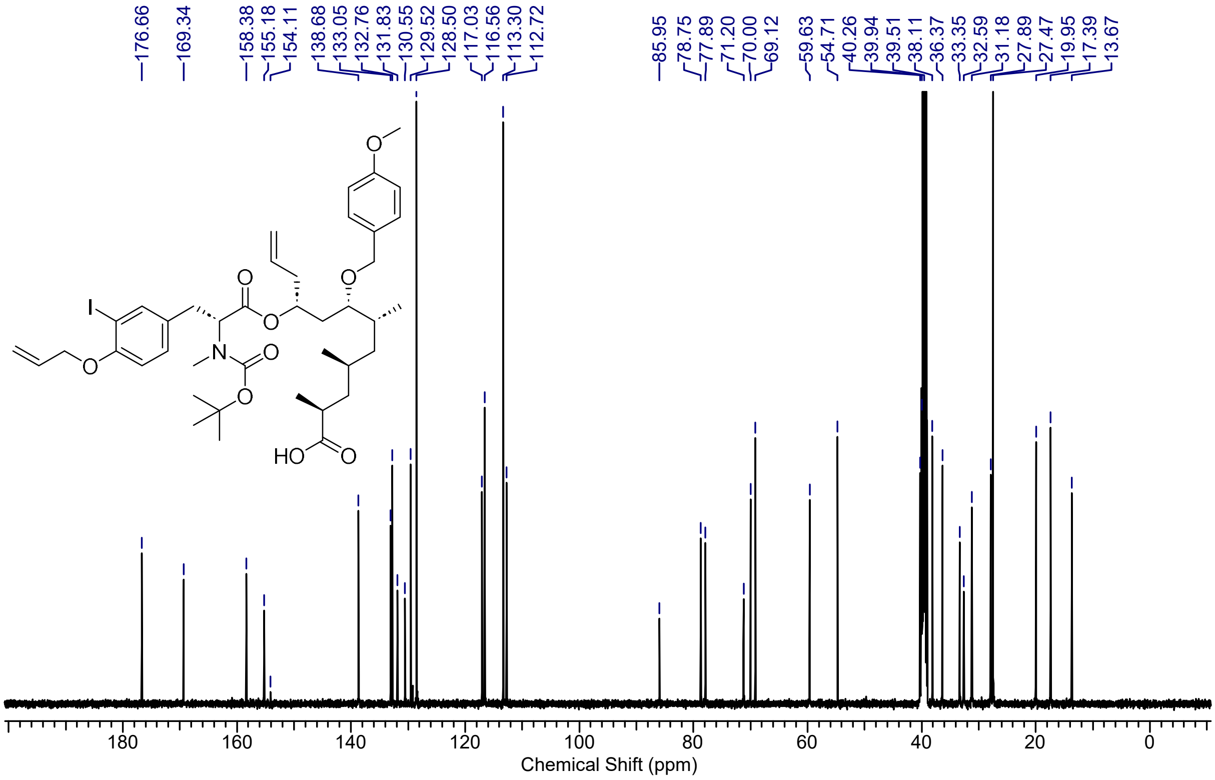

(4*R*,6*S*,7*R*,9*S*,11*S*)-12-{[2-(*tert*-Butoxy)-2-oxoethyl]amino}-6-[(4-methoxybenzyl)oxy]-7,9,11-trimethyl-12-oxo-1-(trimethylsilyl)dodec-1-yn-4-yl (*R*)-3-[4-(allyloxy)-3-iodophenyl]-2-[(*tert*-butoxy-carbonyl)(methyl)amino]propanoate (7a)

^{1}H-NMR (500 MHz, $CDCl_3$)

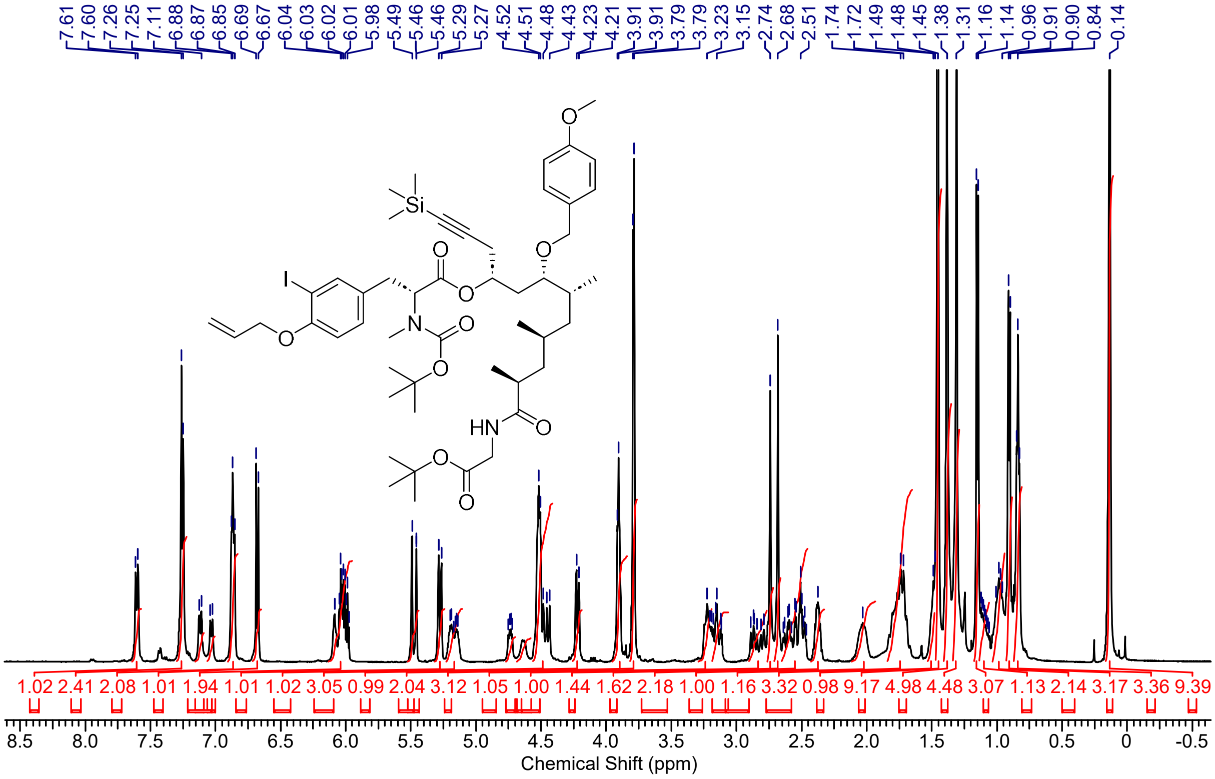

^{13}C-NMR (125 MHz, $CDCl_3$)

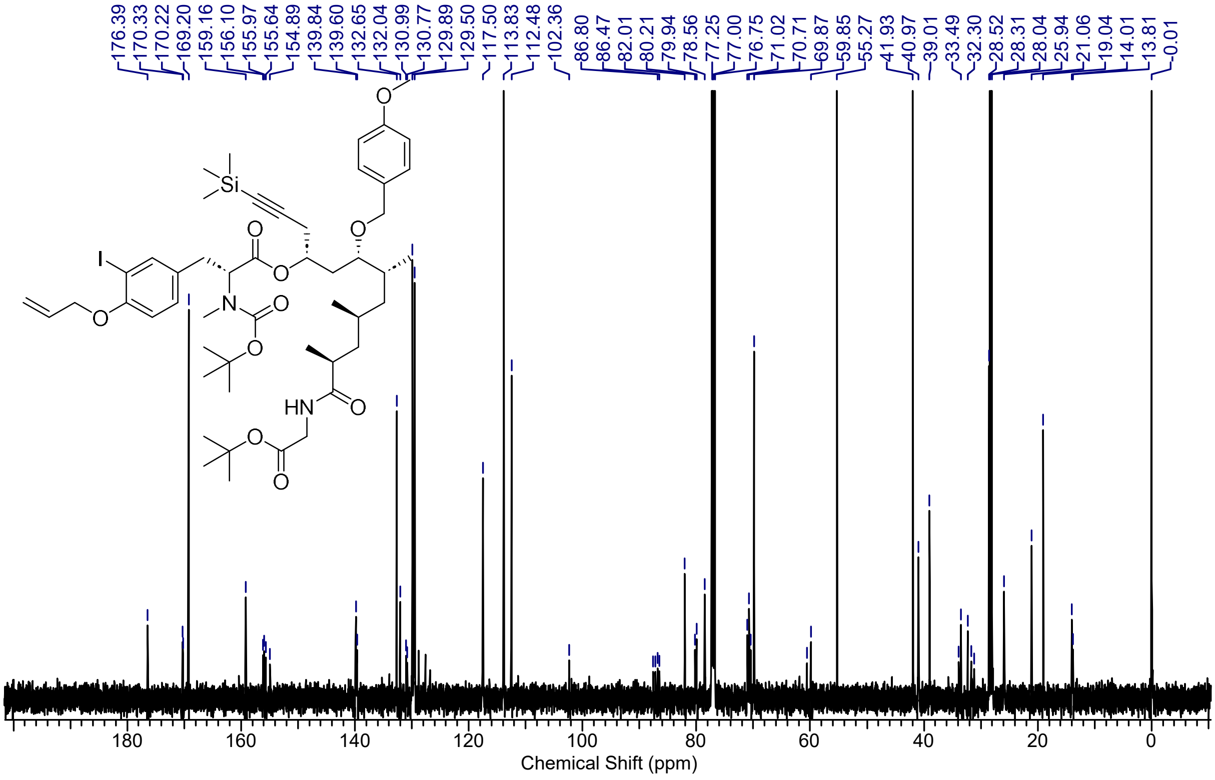

(4*R*,6*S*,7*R*,9*S*,11*S*)-12-{[2-(*tert*-Butoxy)-2-oxoethyl]amino}-6-[(4-methoxybenzyl)oxy]-7,9,11-trimethyl-12-oxododec-1-en-4-yl (*R*)-3-[4-(allyloxy)-3-iodophenyl]-2-[(*tert*-butoxycarbonyl)(methyl)-amino]propanoate (7b)

^{1}H-NMR (500 MHz, 373 K, DMSO-D$_6$)

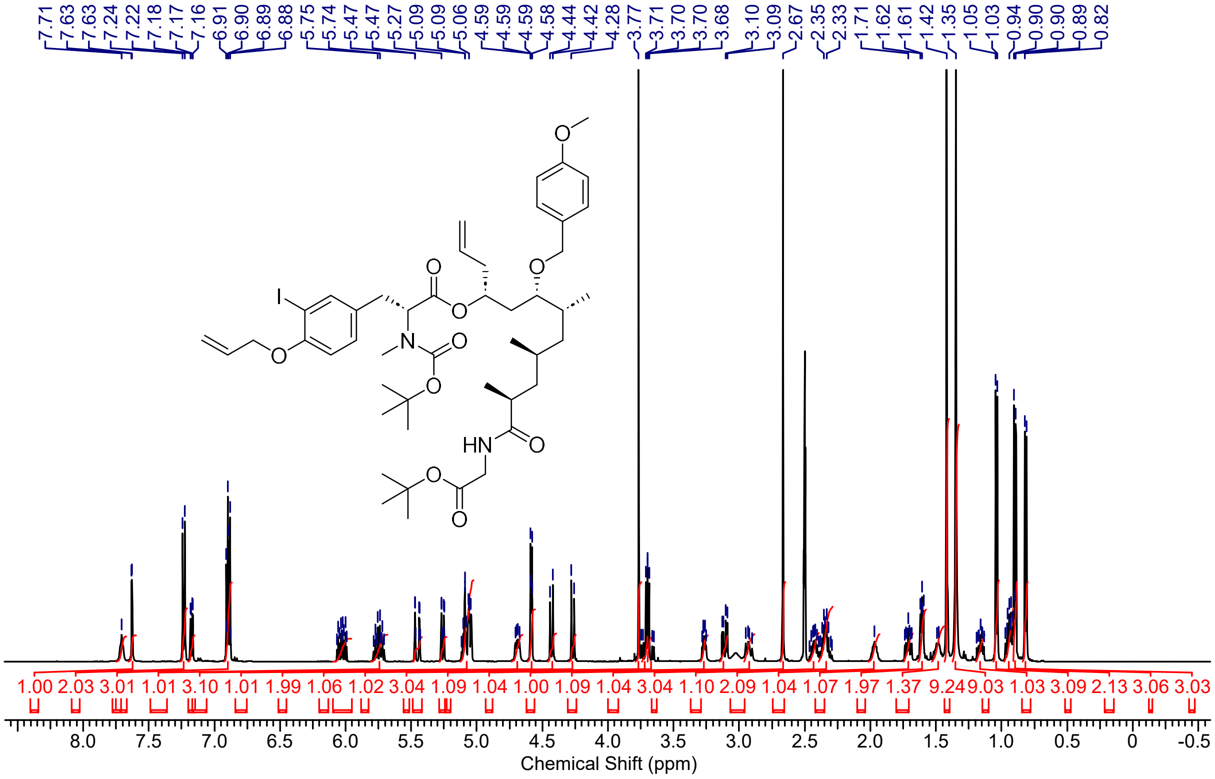

^{13}C-NMR (125 MHz, 373 K, DMSO-D$_6$)

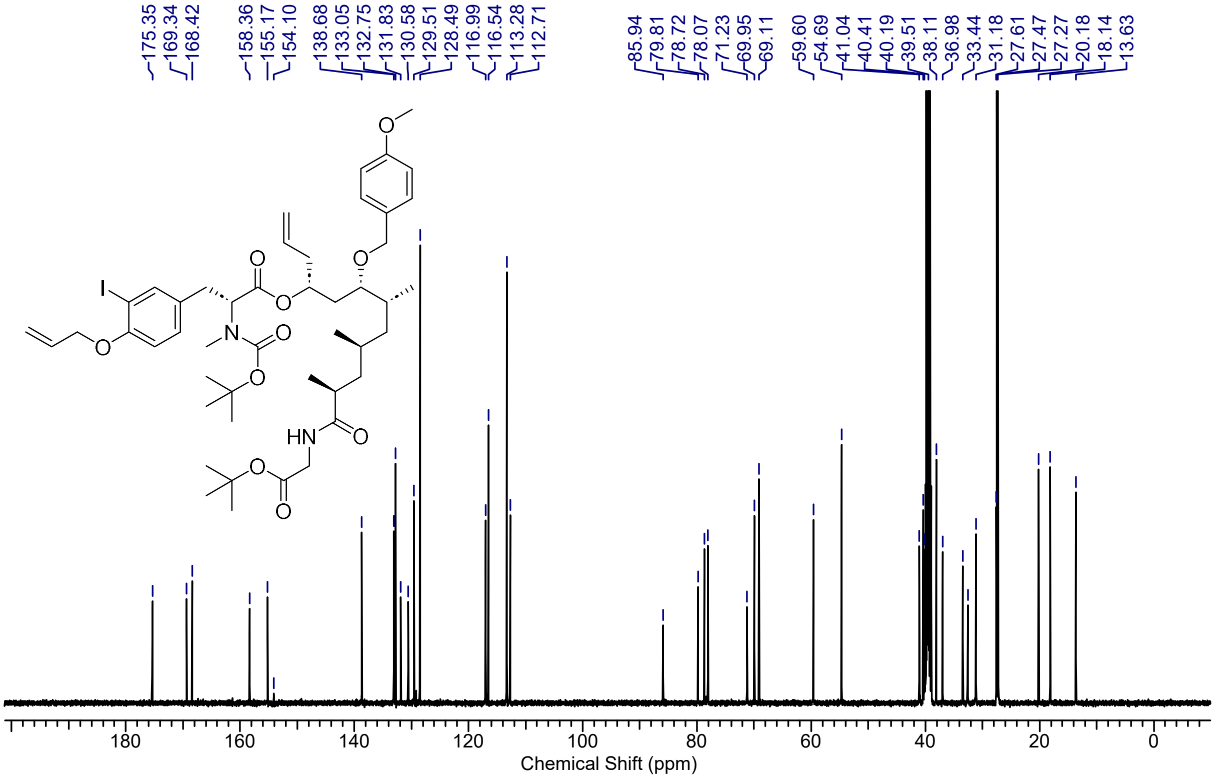

(3*R*,9*S*,11*S*,13*R*,14*S*,16*R*)-3-[4-(Allyloxy)-3-iodobenzyl]-14-hydroxy-4,9,11,13-tetramethyl-16-[3-(trimethylsilyl)prop-2-yn-1-yl]-1-oxa-4,7-diazacyclohexadecane-2,5,8-trione (7a-1)

^{1}H-NMR (400 MHz, $CDCl_3$)

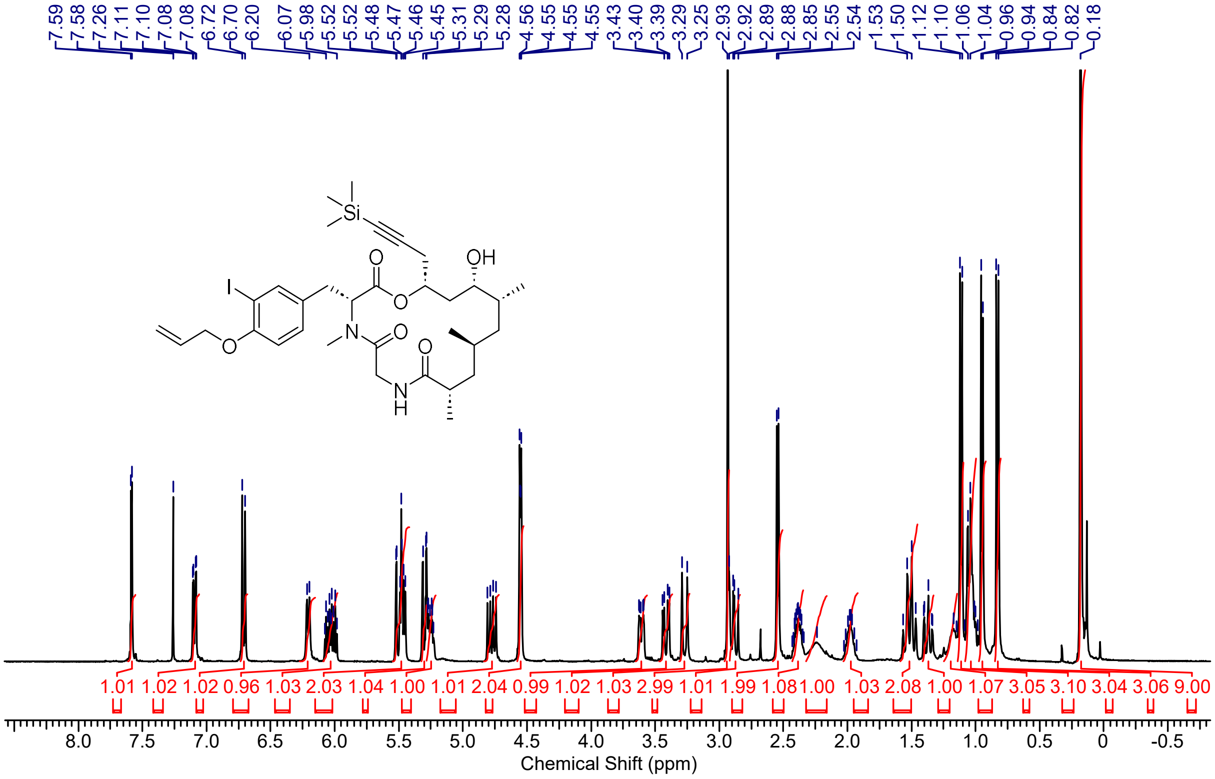

^{13}C-NMR (100 MHz, $CDCl_3$)

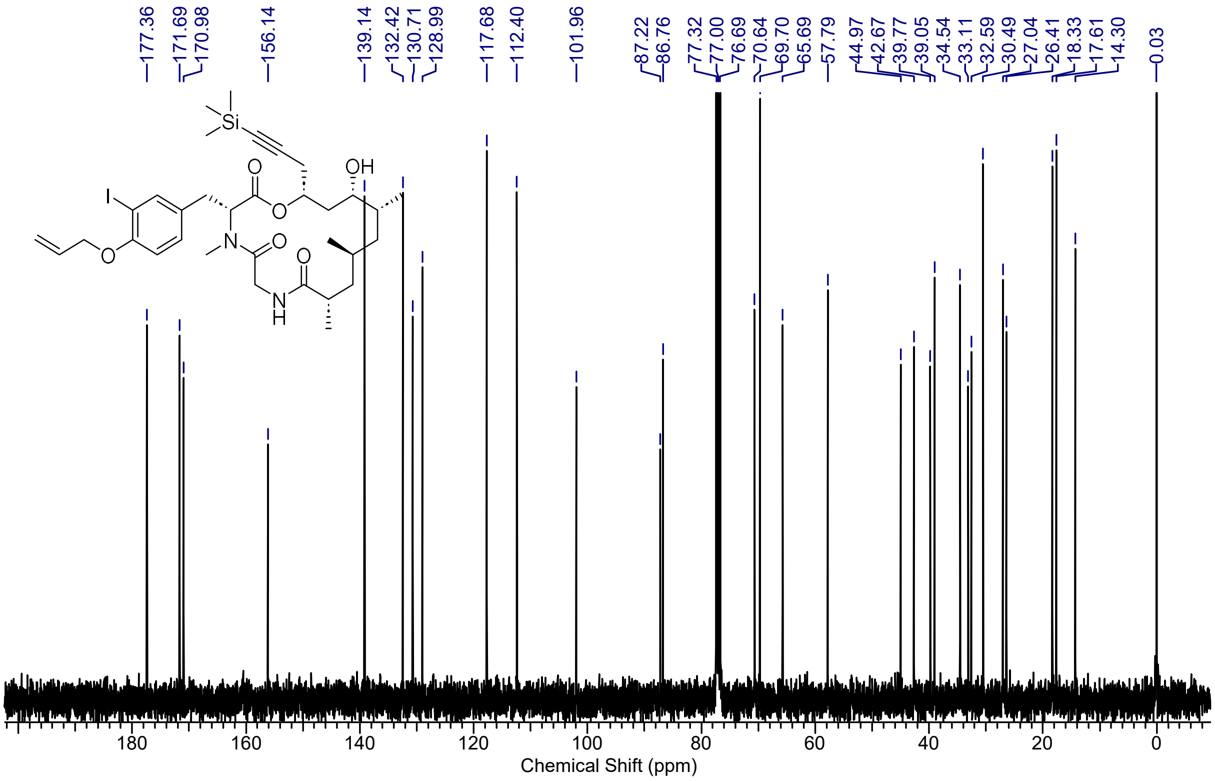

(3*R*,9*S*,11*S*,13*R*,14*S*,16*R*)-16-allyl-3-[4-(allyloxy)-3-iodobenzyl]-14-hydroxy-4,9,11,13-tetramethyl-1-oxa-4,7-diazacyclohexadecane-2,5,8-trione (7b-1)

^{1}H-NMR (400 MHz, $CDCl_3$)

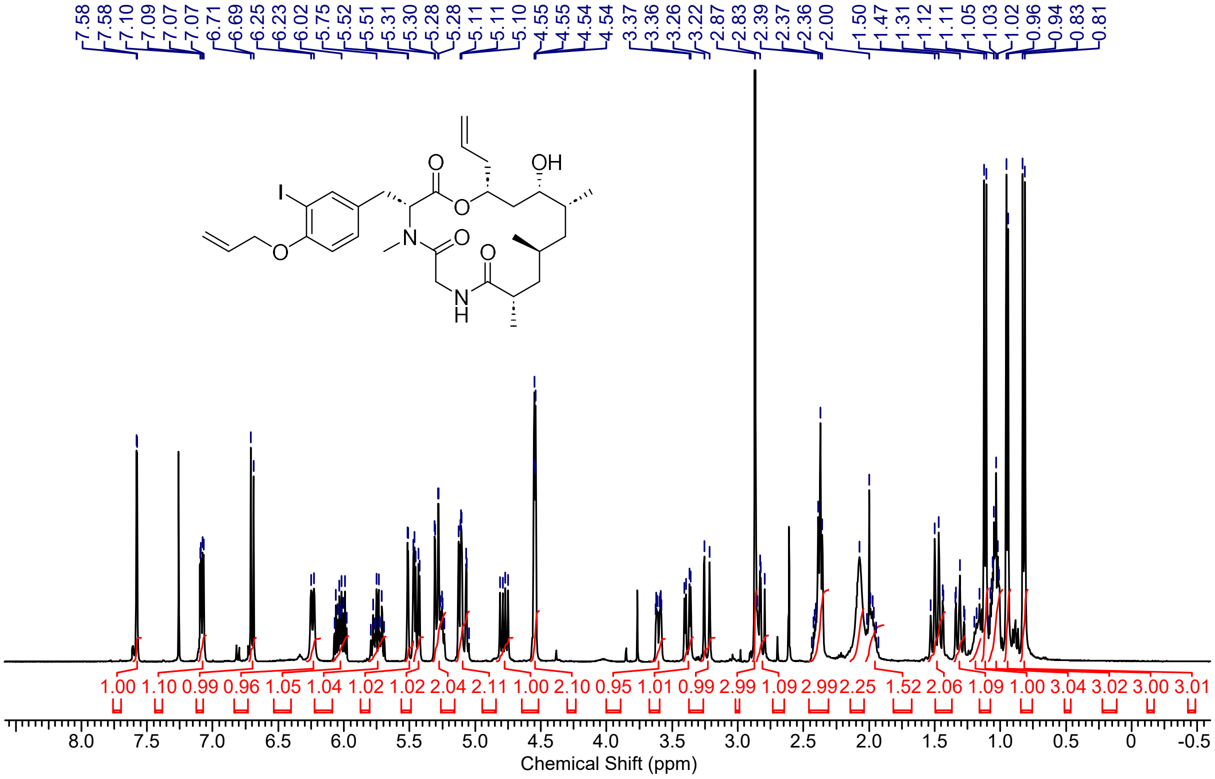

^{13}C-NMR (100 MHz, $CDCl_3$)

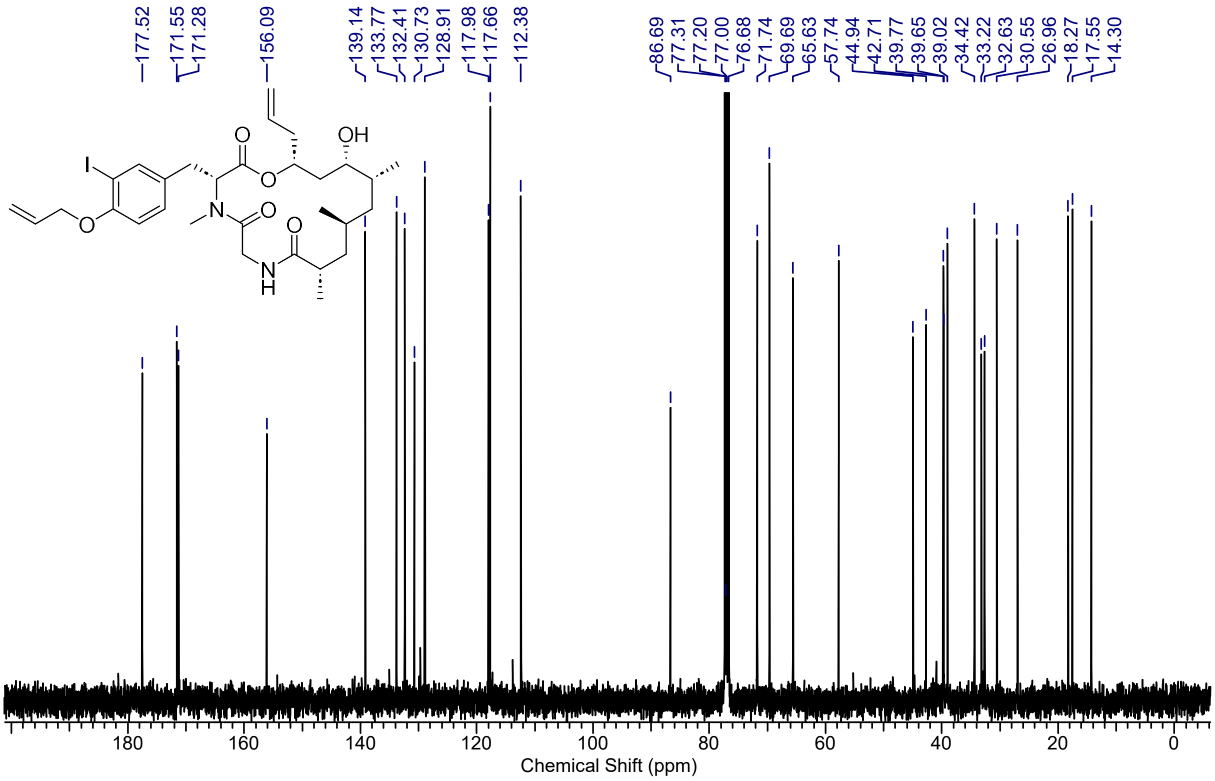

(3*R*,9*S*,11*S*,13*R*,14*S*,16*R*)-14-Hydroxy-3-(4-hydroxy-3-iodobenzyl)-4,9,11,13-tetramethyl-16-[3-(trimethylsilyl)prop-2-yn-1-yl]-1-oxa-4,7-diazacyclohexadecane-2,5,8-trione (8a)

^{1}H-NMR (400 MHz, $CDCl_3$)

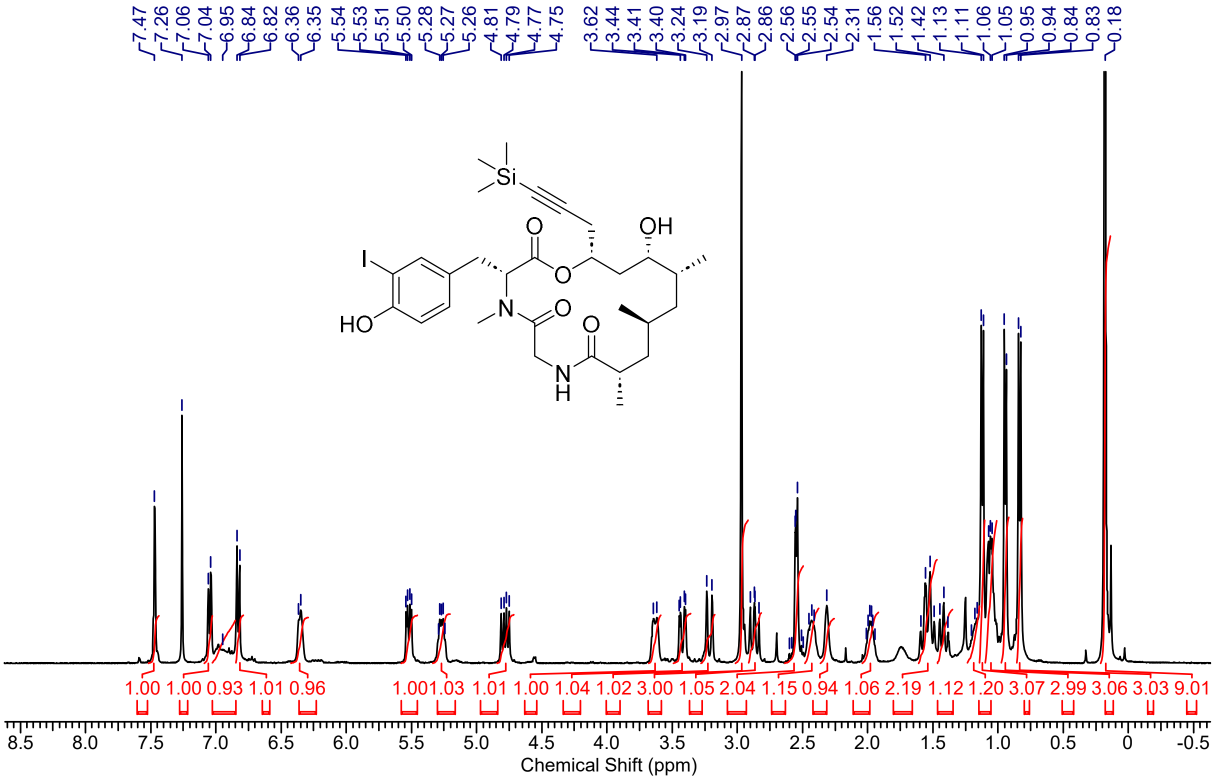

^{13}C-NMR (100 MHz, $CDCl_3$)

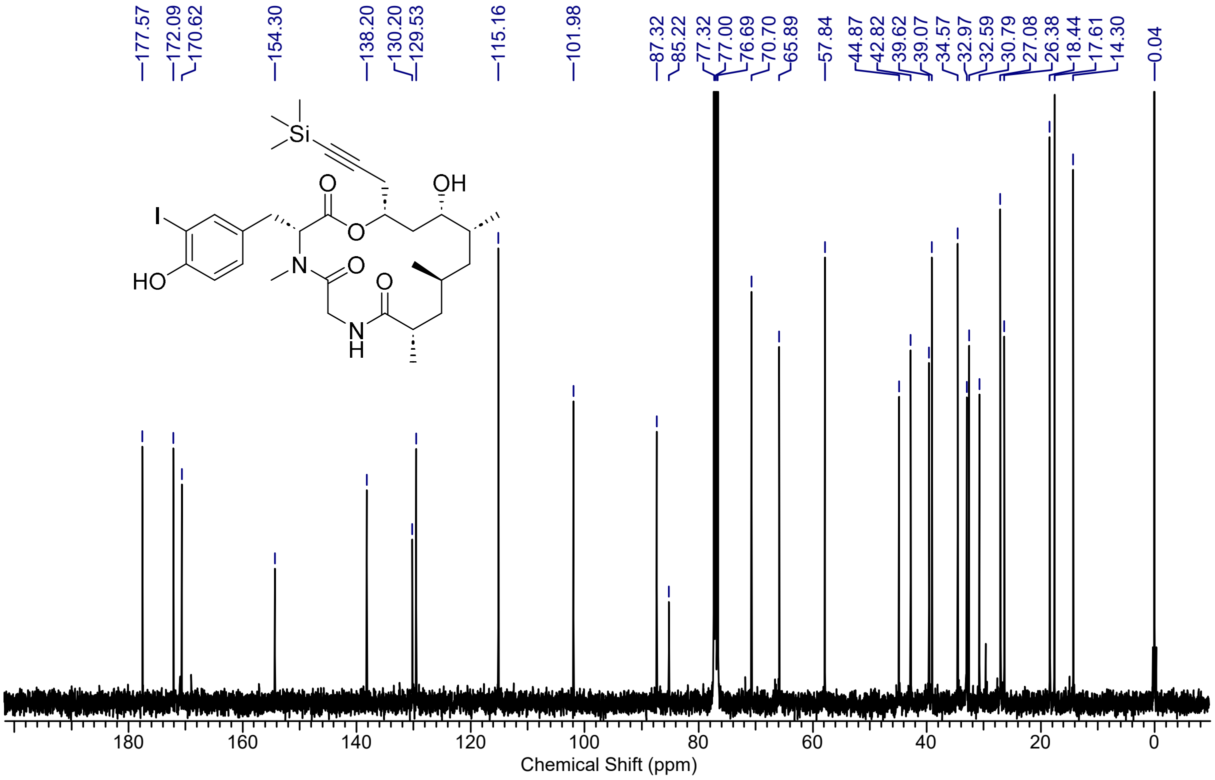

(3*R*,9*S*,11*S*,13*R*,14*S*,16*R*)-16-Allyl-14-hydroxy-3-(4-hydroxy-3-iodobenzyl)-4,9,11,13-tetramethyl-1-oxa-4,7-diazacyclohexadecane-2,5,8-trione (8b)

^{1}H-NMR (500 MHz, $CDCl_3$)

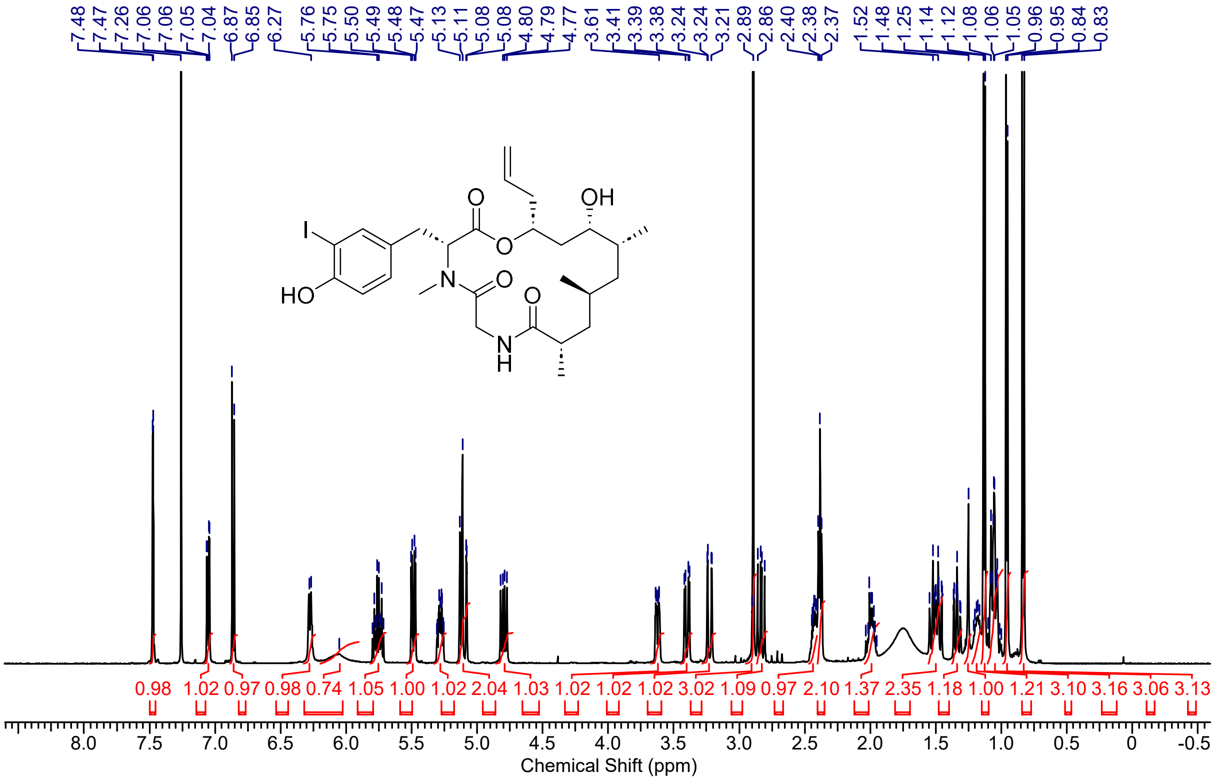

^{13}C-NMR (125 MHz, $CDCl_3$)

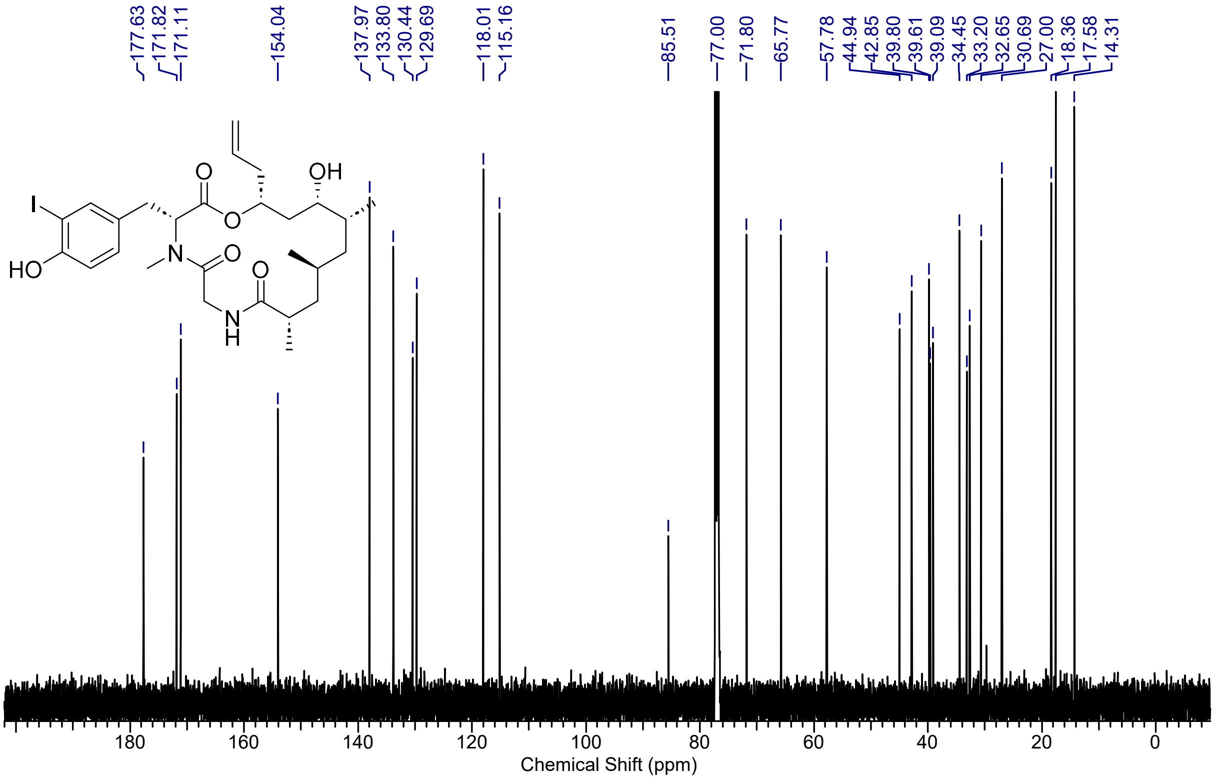

(3*R*,9*S*,11*S*,13*R*,14*S*,16*R*)-14-Hydroxy-3-(4-hydroxy-3-iodobenzyl)-4,9,11,13-tetramethyl-16-(prop-2-yn-1-yl)-1-oxa-4,7-diazacyclohexadecane-2,5,8-trione (8c)

^{1}H-NMR (500 MHz, $CDCl_3$)

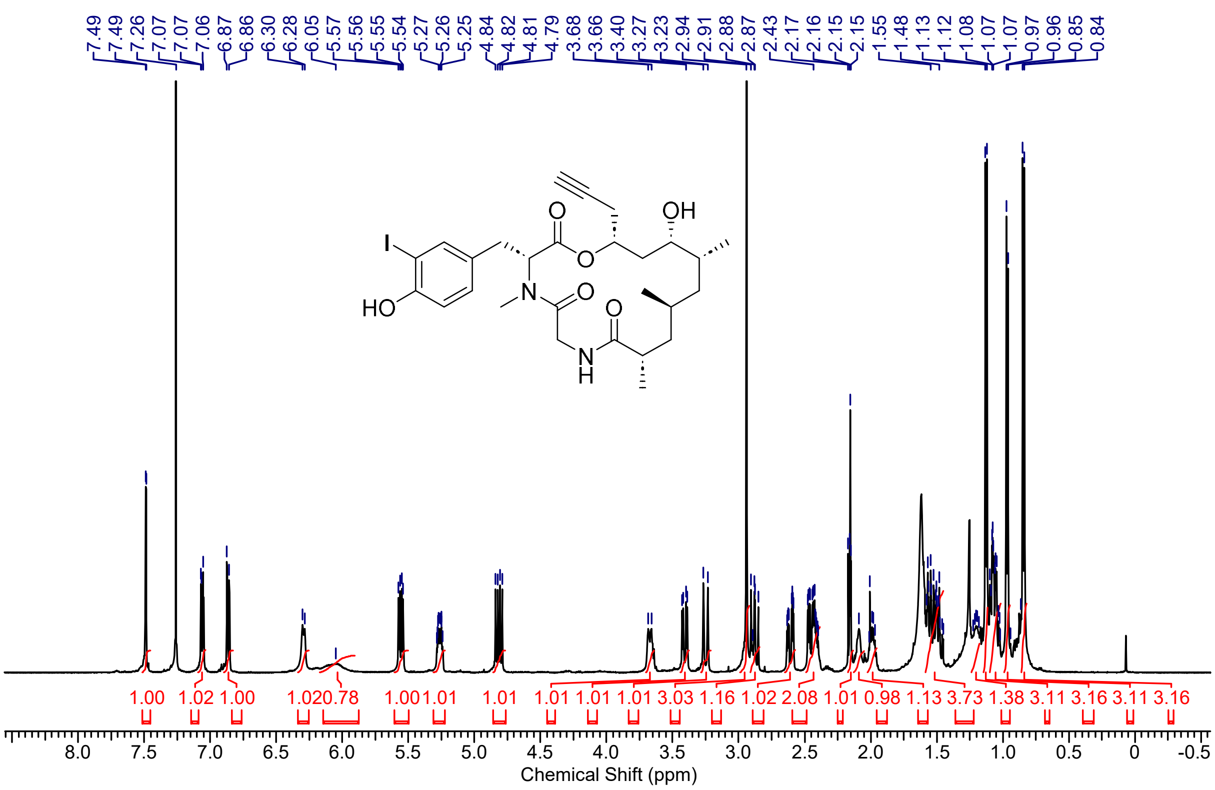

^{13}C-NMR (125 MHz, $CDCl_3$)

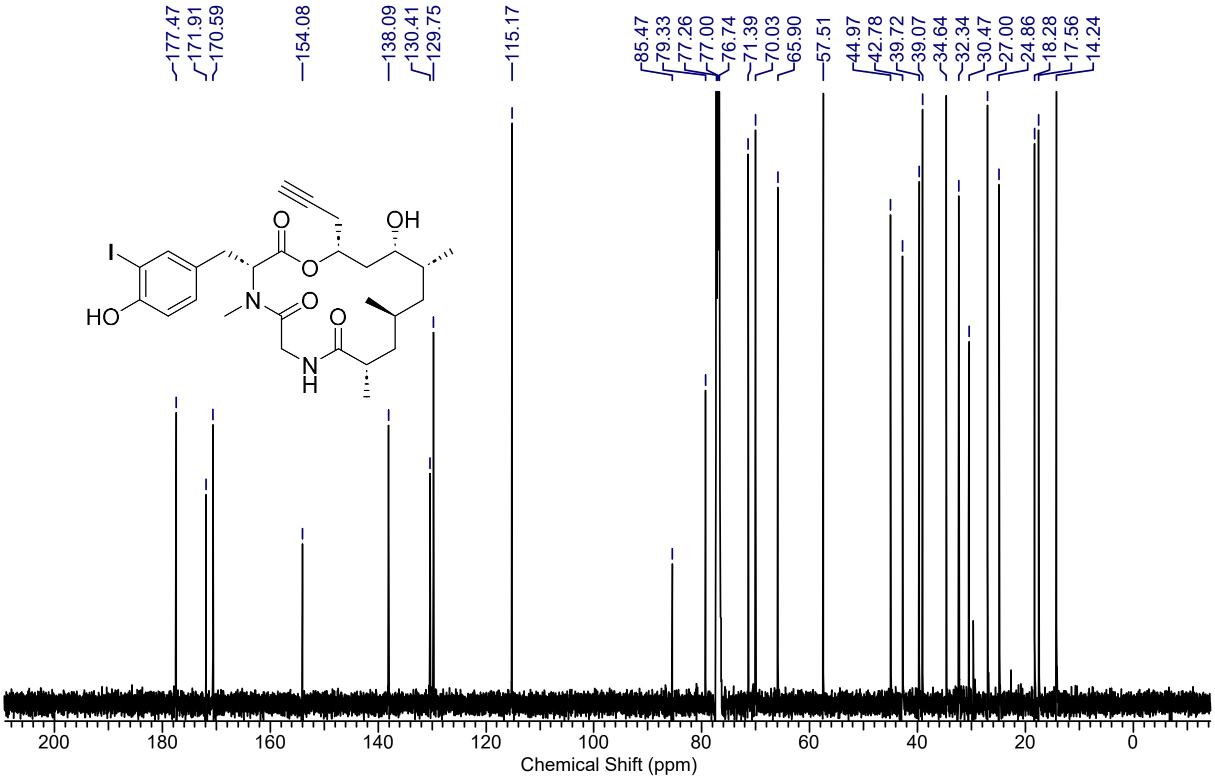

(3*R*,9*S*,11*S*,13*R*,14*S*,16*R*)-16-[(1-Benzyl-1H-1,2,3-triazol-4-yl)methyl]-14-hydroxy-3-(4-hydroxy-3-iodobenzyl)-4,9,11,13-tetramethyl-1-oxa-4,7-diazacyclohexadecane-2,5,8-trione (9a)

^{1}H-NMR (500 MHz, $CDCl_3$)

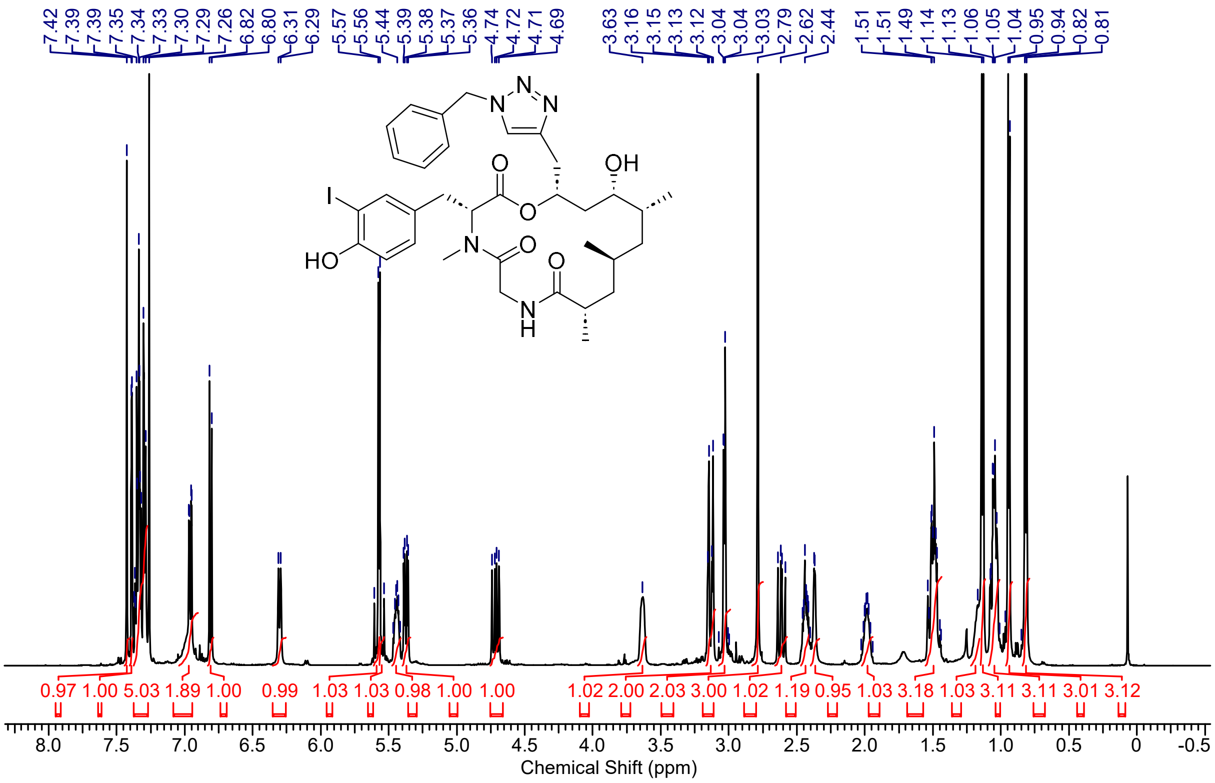

^{13}C-NMR (125 MHz, $CDCl_3$)

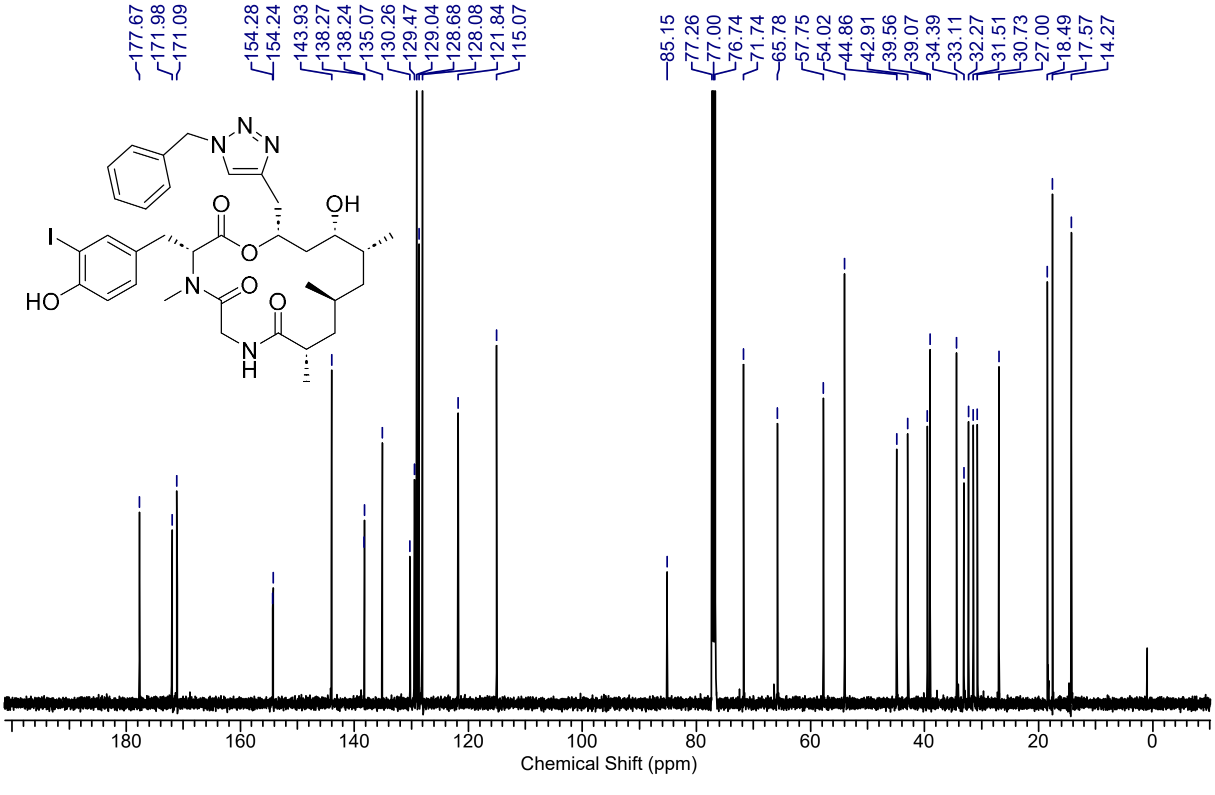

(3*R*,9*S*,11*S*,13*R*,14*S*,16*R*)-14-hydroxy-3-(4-hydroxy-3-iodobenzyl)-4,9,11,13-tetramethyl-16-((1-pentyl-1H-1,2,3-triazol-4-yl)methyl)-1-oxa-4,7-diazacyclohexadecane-2,5,8-trione (9b)

^{1}H-NMR (500 MHz, $CDCl_3$)

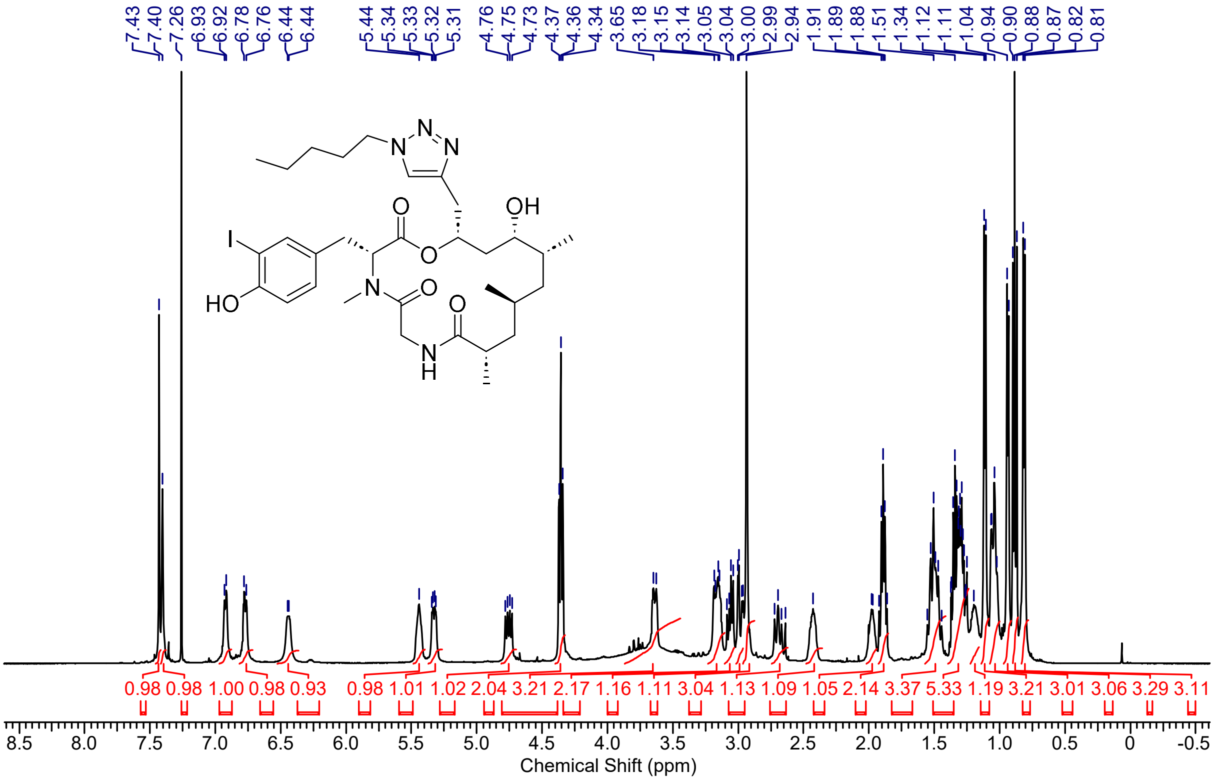

^{13}C-NMR (125 MHz, $CDCl_3$)

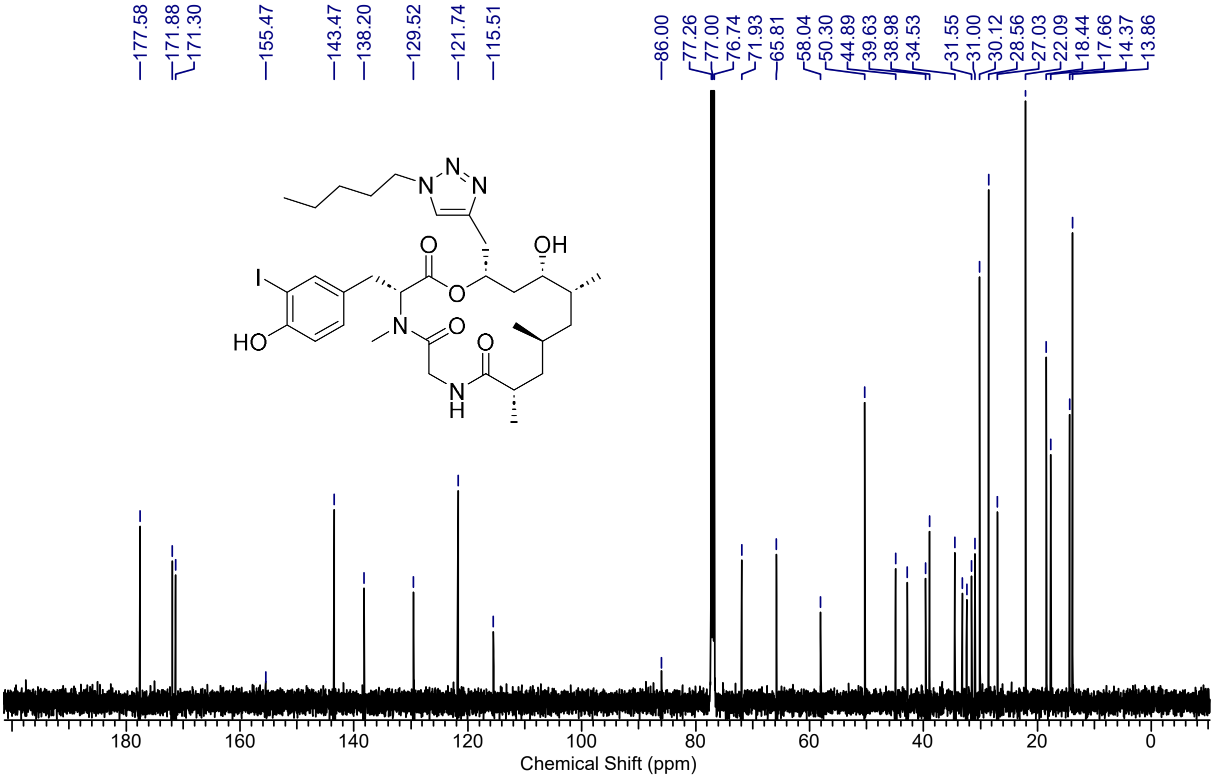

Benzyl 2-(4-{[(3*R*,9*S*,11*S*,13*R*,14*S*,16*R*)-14-hydroxy-3-(4-hydroxy-3-iodobenzyl)-4,9,11,13-tetramethyl-2,5,8-trioxo-1-oxa-4,7-diazacyclohexadecan-16-yl]methyl}-1H-1,2,3-triazol-1-yl)acetate (9c)

^{1}H-NMR (500 MHz, $CDCl_3$)

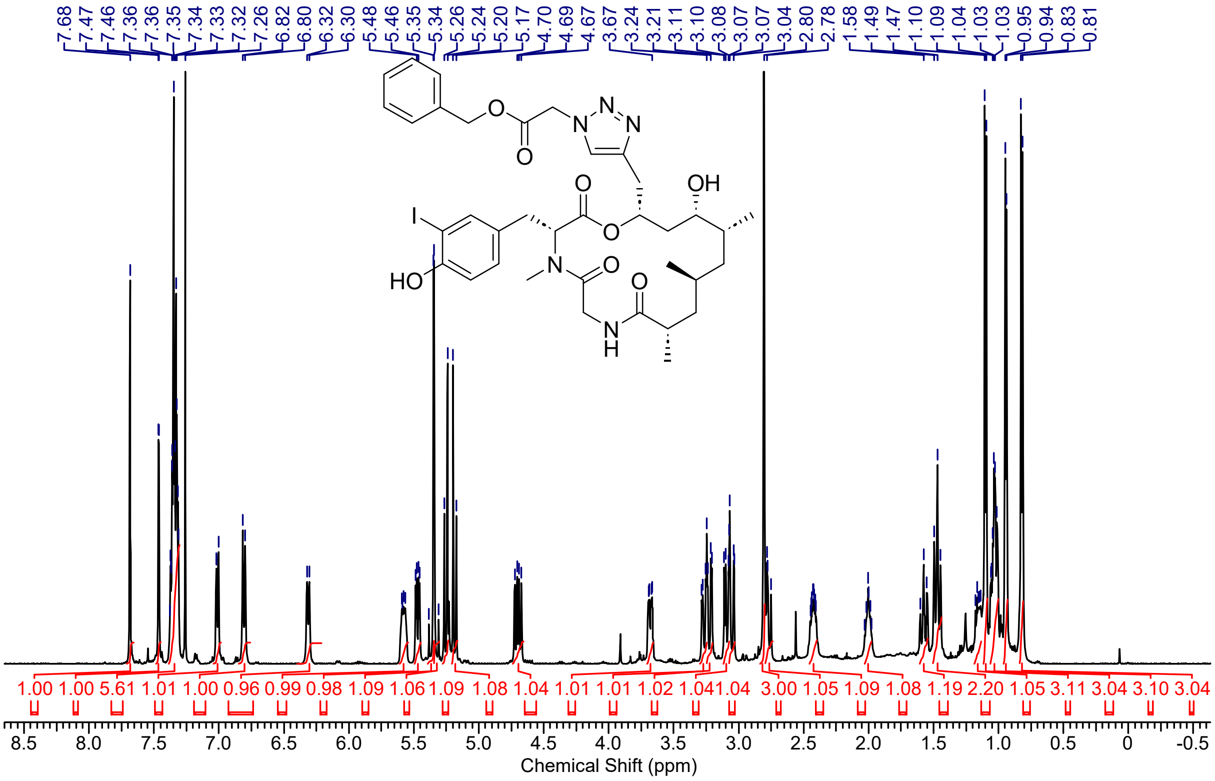

^{13}C-NMR (125 MHz, $CDCl_3$)

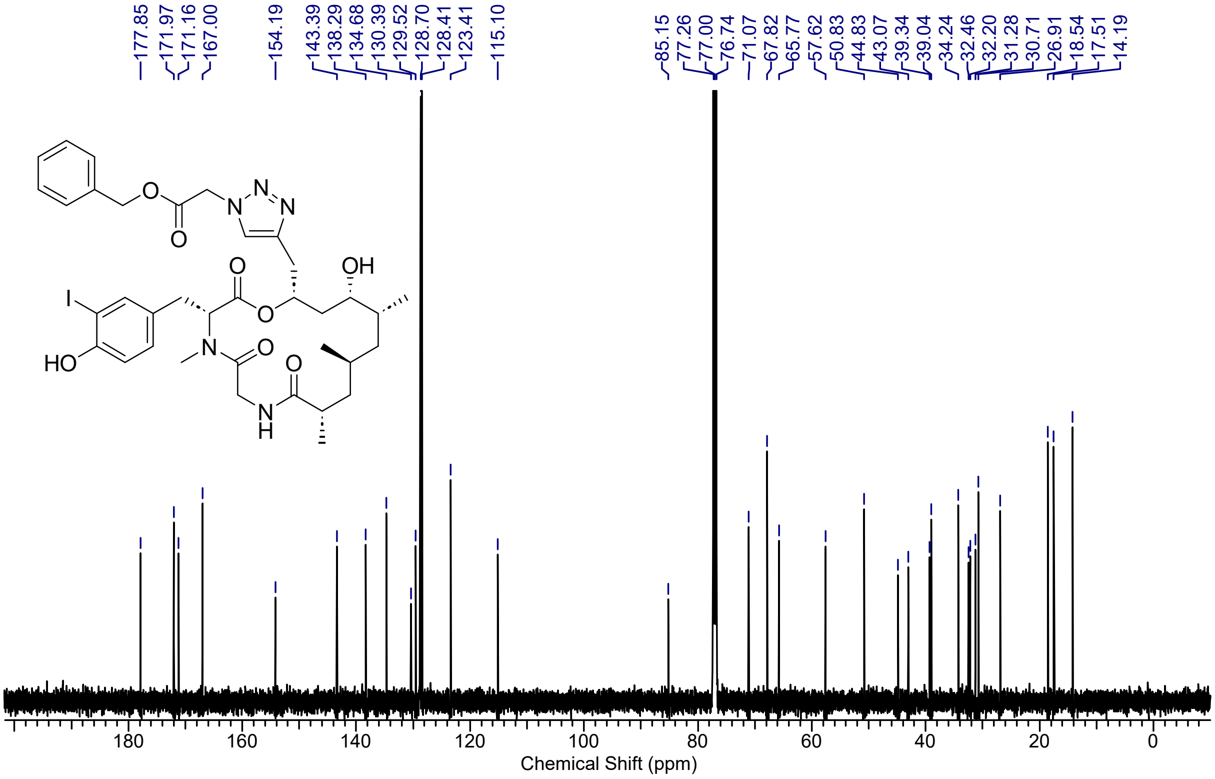

2-{2-[2-(4-{[(3*R*,9*S*,11*S*,13*R*,14*S*,16*R*)-14-Hydroxy-3-(4-hydroxy-3-iodobenzyl)-4,9,11,13-tetra-methyl-2,5,8-trioxo-1-oxa-4,7-diazacyclohexadecan-16-yl]methyl}-1H-1,2,3-triazol-1-yl)ethoxy]-ethoxy}acetic acid (9d)

^{1}H-NMR (500 MHz, DMSO-D$_6$)

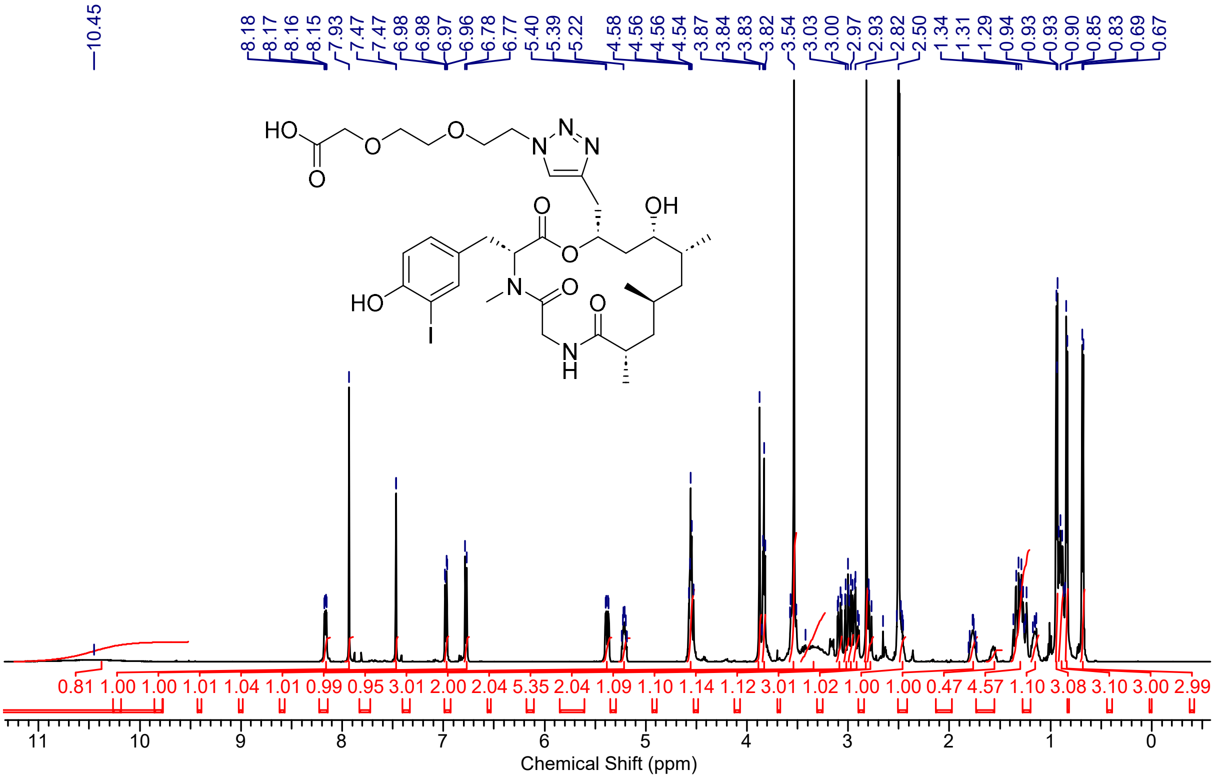

^{13}C-NMR (125 MHz, DMSO-D$_6$)

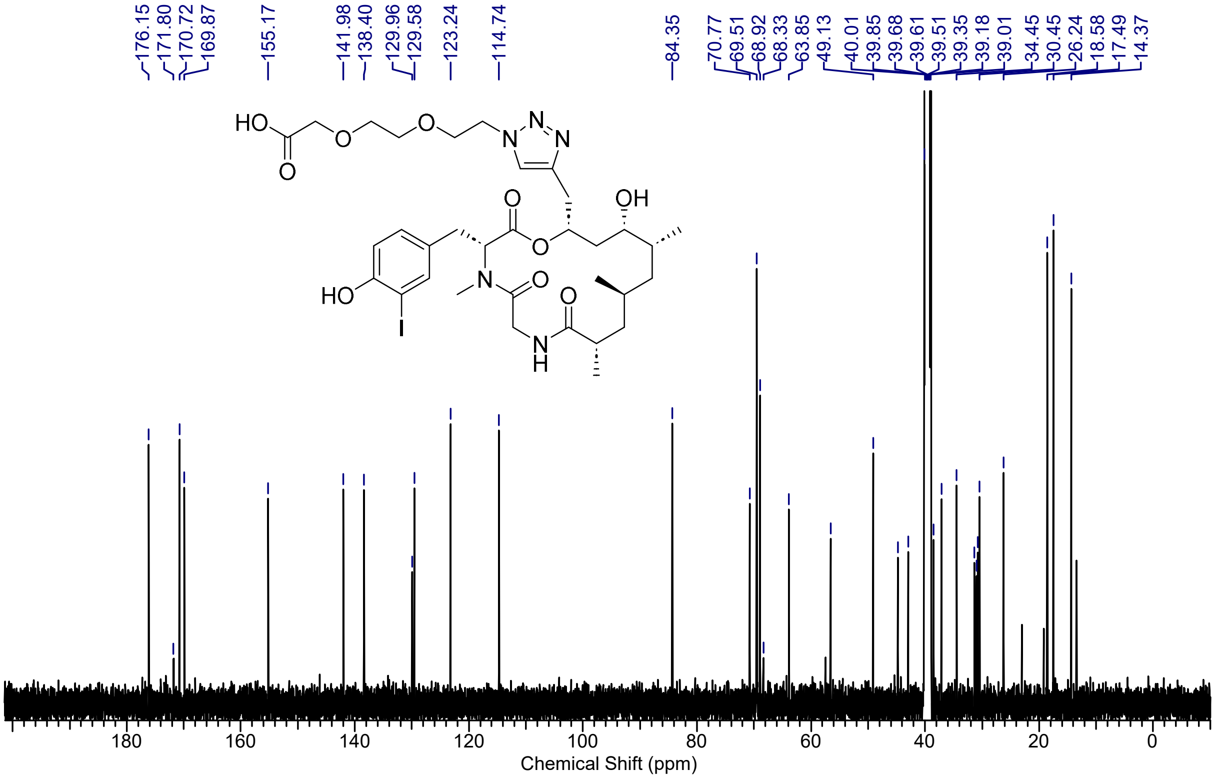

(3*R*,9*S*,11*S*,13*R*,14*S*,16*R*)-14-Hydroxy-3-(4-hydroxy-3-iodobenzyl)-16-((1-(2-(2-(2-hydroxy-ethoxy)ethoxy)ethyl)-1H-1,2,3-triazol-4-yl)methyl)-4,9,11,13-tetramethyl-1-oxa-4,7-diaza-cyclohexadecane-2,5,8-trione (9e)

^{1}H-NMR (500 MHz, $CDCl_3$)

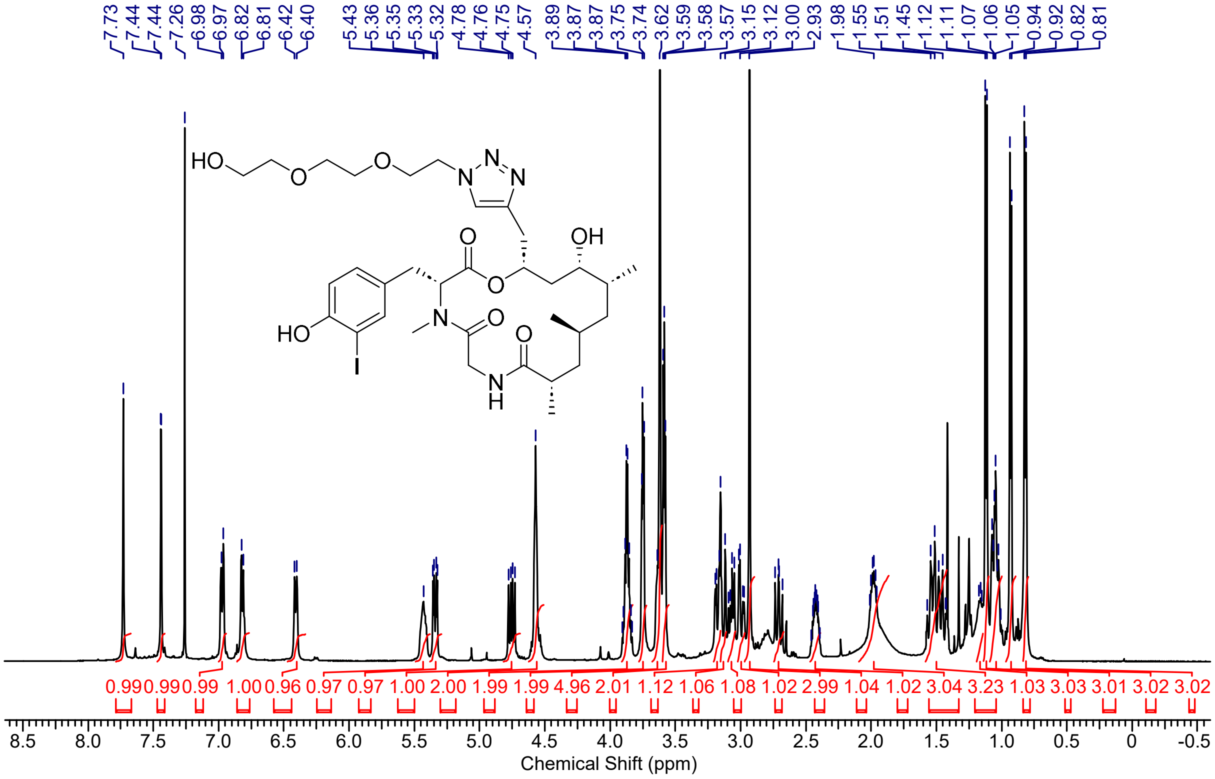

^{13}C-NMR (125 MHz, $CDCl_3$)

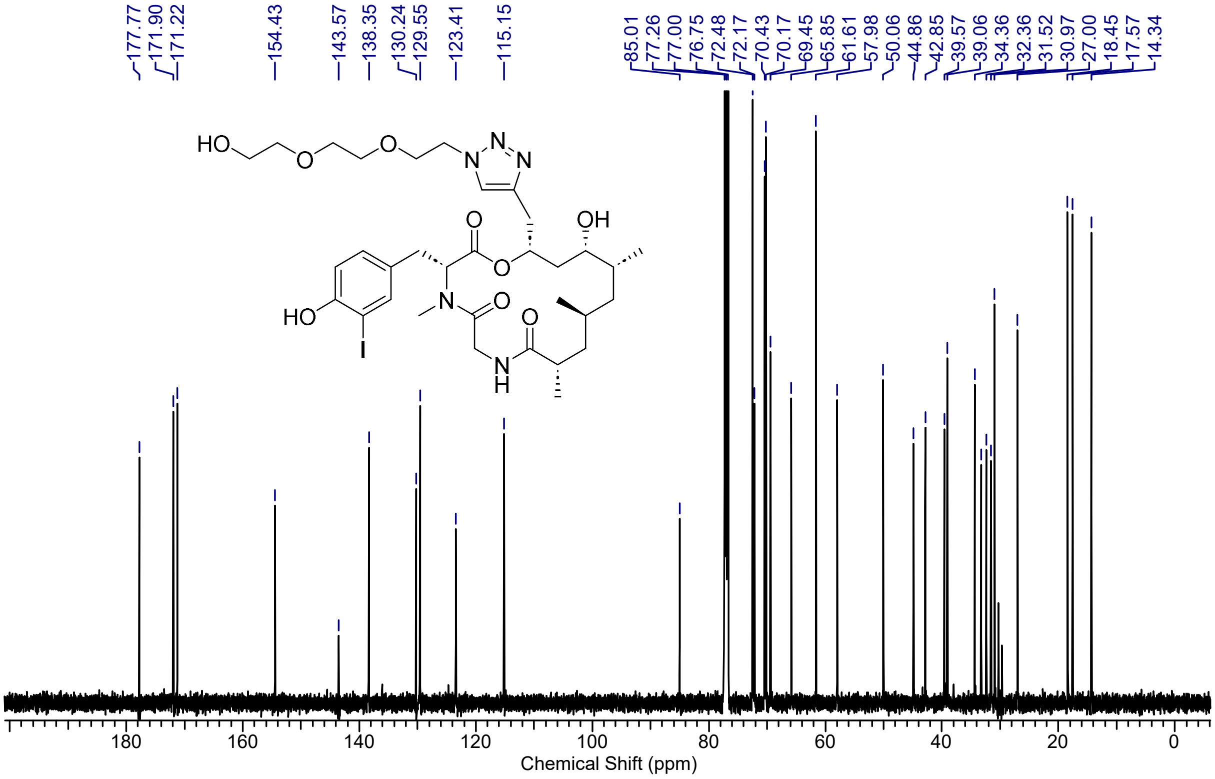

tert-Butyl (2-{2-[2-(4-{[(3_R_,9_S_,11_S_,13_R_,14_S_,16_R_)-14-hydroxy-3-(4-hydroxy-3-iodobenzyl)-4,9,11,13-tetramethyl-2,5,8-trioxo-1-oxa-4,7-diazacyclohexadecan-16-yl]methyl}-1H-1,2,3-triazol-1-yl)ethoxy]ethoxy}ethyl)carbamate (9f)

^{1}H-NMR (500 MHz, DMSO-D$_6$)

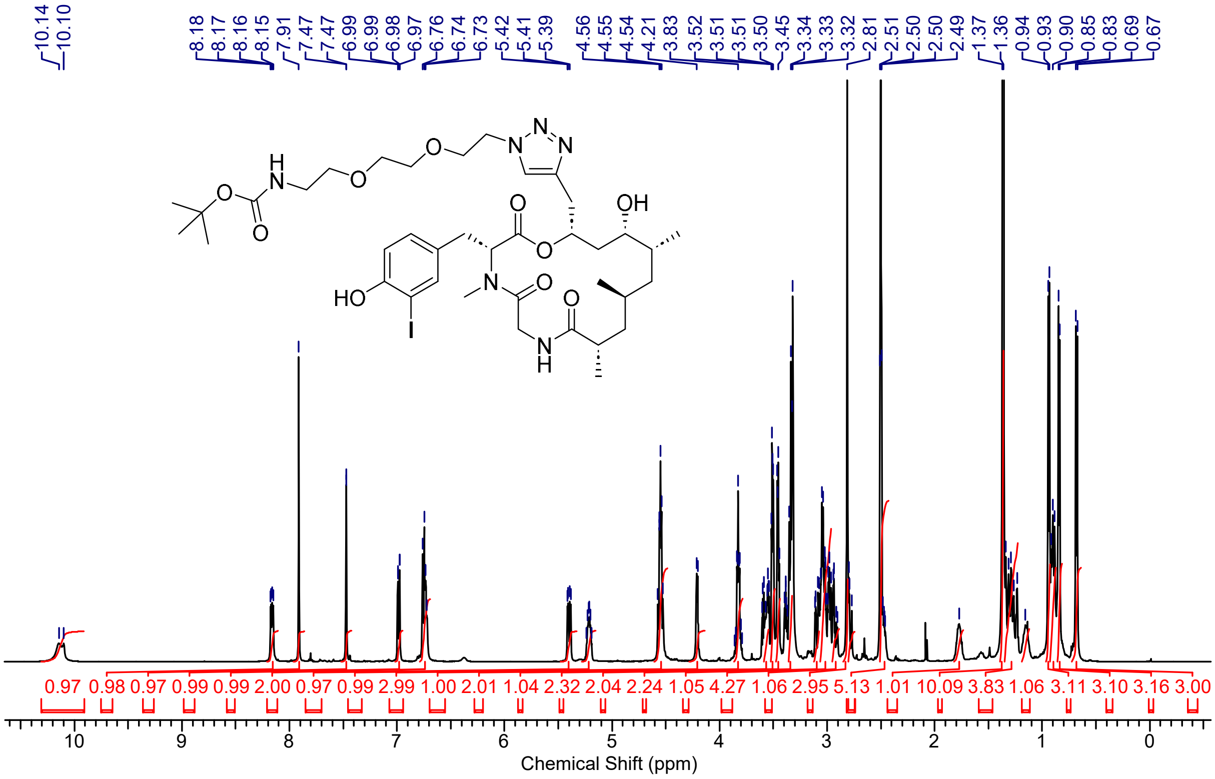

^{13}C-NMR (125 MHz, DMSO-D$_6$)

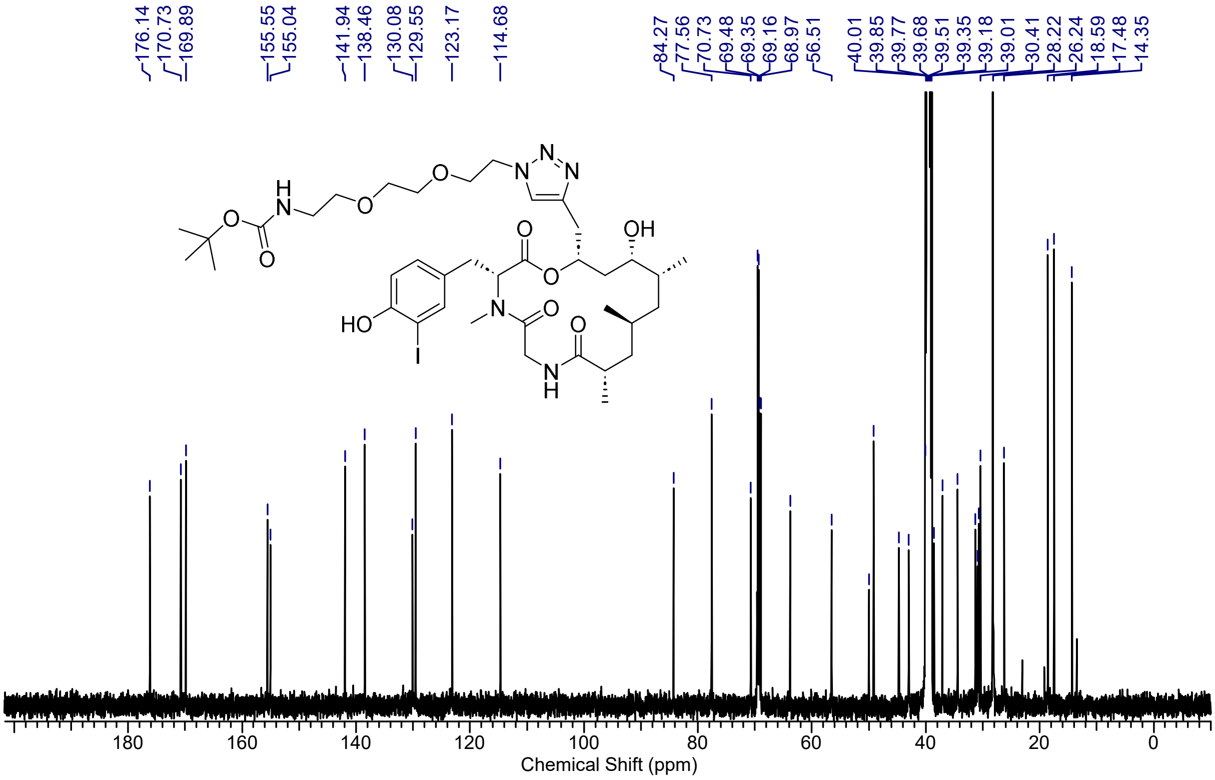

***tert*-Butyl [2-(2-{2-[2-(4-{[(3*R*,9*S*,11*S*,13*R*,14*S*,16*R*)-14-hydroxy-3-(4-hydroxy-3-iodobenzyl)-4,9,11,13-tetramethyl-2,5,8-trioxo-1-oxa-4,7-diazacyclohexadecan-16-yl]methyl}-1H-1,2,3-triazol-1-yl)ethoxy]ethoxy}ethoxy)ethyl]carbamate (9g)**

^{1}H-NMR (500 MHz, DMSO-D$_6$)

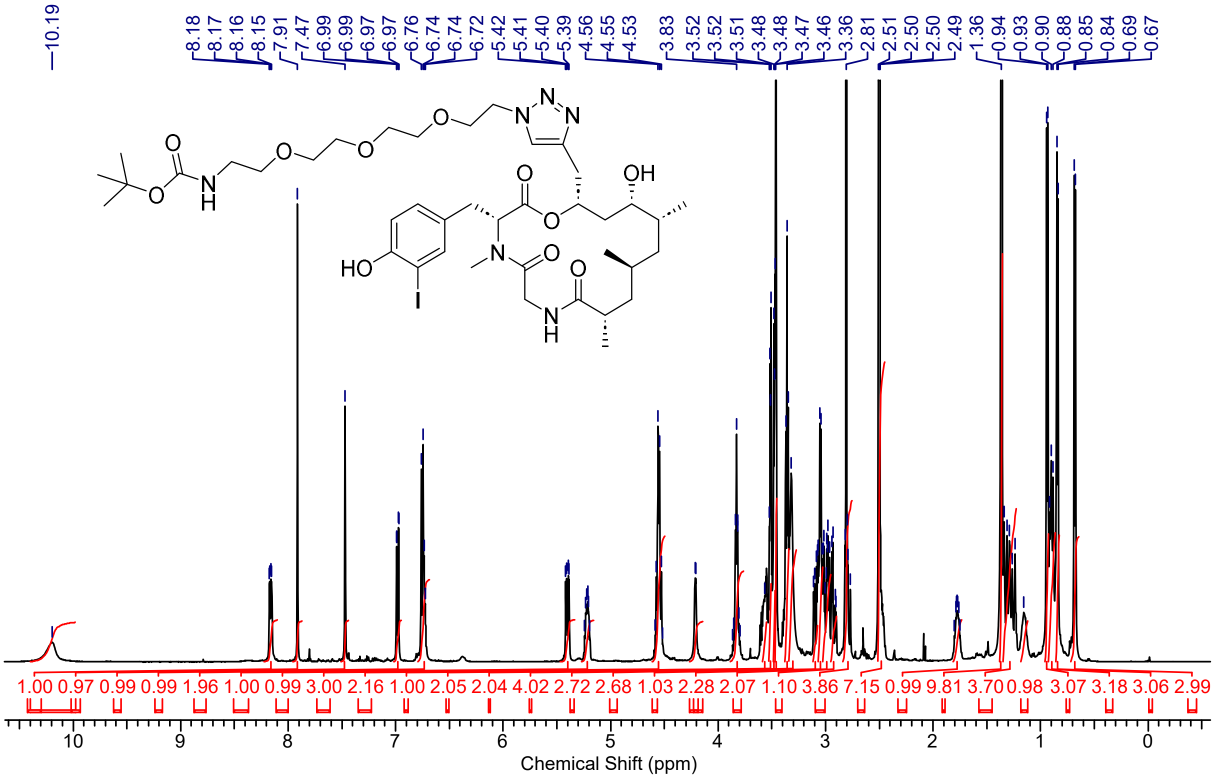

^{13}C-NMR (125 MHz, DMSO-D$_6$)

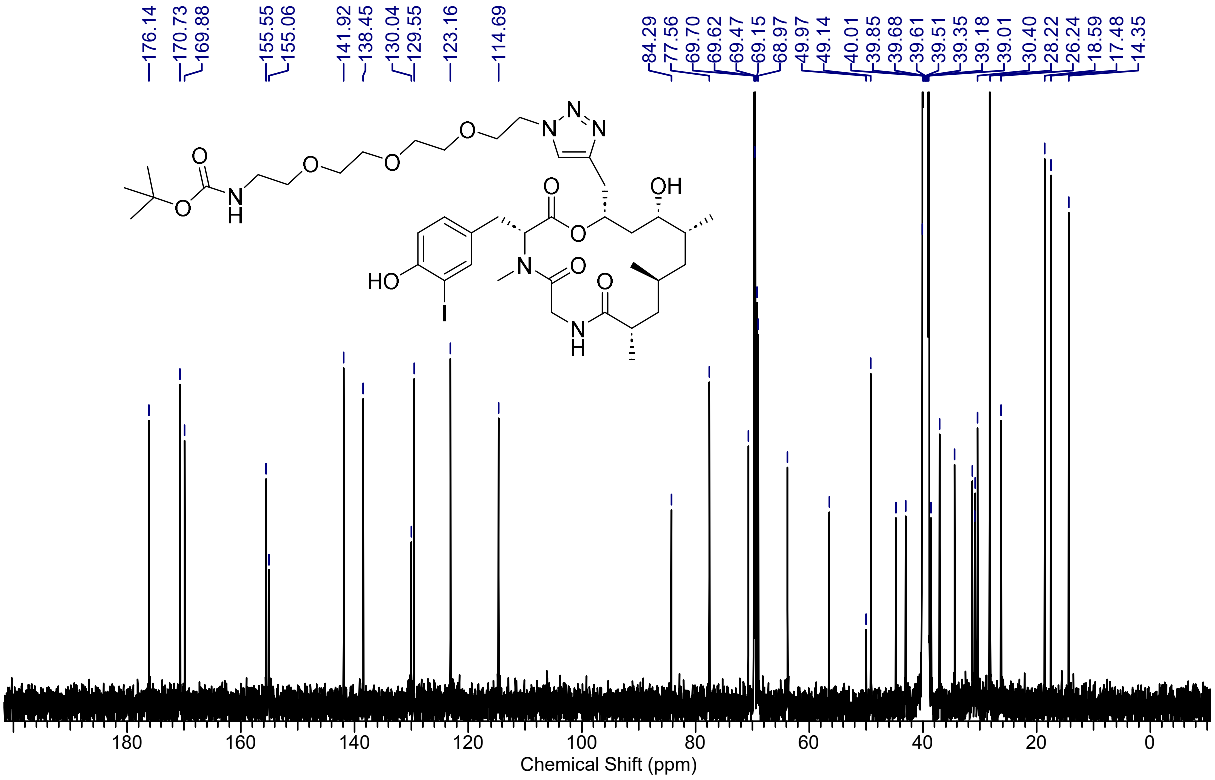

(3*R*,9*S*,11*S*,13*R*,14*S*,16*R*)-14-Hydroxy-3-(4-hydroxy-3-iodobenzyl)-4,9,11,13-tetramethyl-16-(3-phenylprop-2-yn-1-yl)-1-oxa-4,7-diazacyclohexadecane-2,5,8-trione (10a)

^{1}H-NMR (500 MHz, $CDCl_3$)

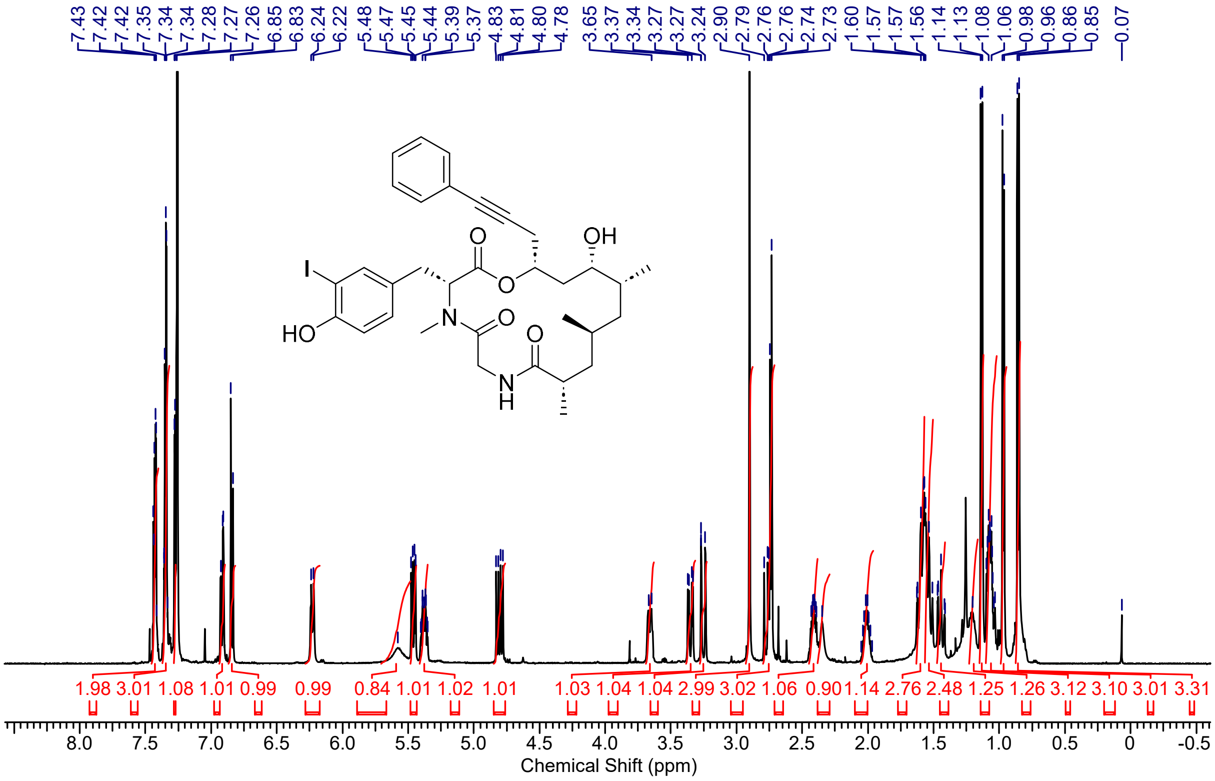

^{13}C-NMR (125 MHz, $CDCl_3$)

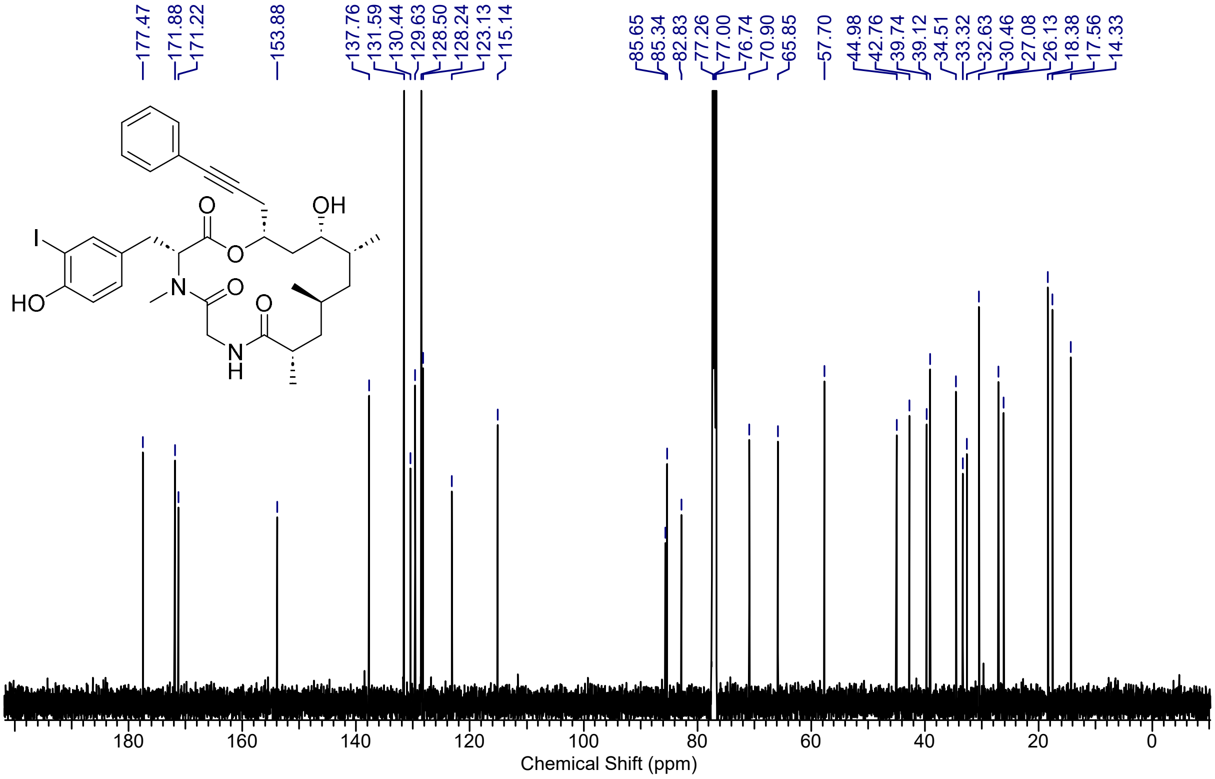

(3*R*,9*S*,11*S*,13*R*,14*S*,16*R*)-14-Hydroxy-3-(4-hydroxy-3-iodobenzyl)-16-[3-(4-methoxyphenyl)prop-2-yn-1-yl]-4,9,11,13-tetramethyl-1-oxa-4,7-diazacyclohexadecane-2,5,8-trione (10b)

^{1}H-NMR (500 MHz, $CDCl_3$)

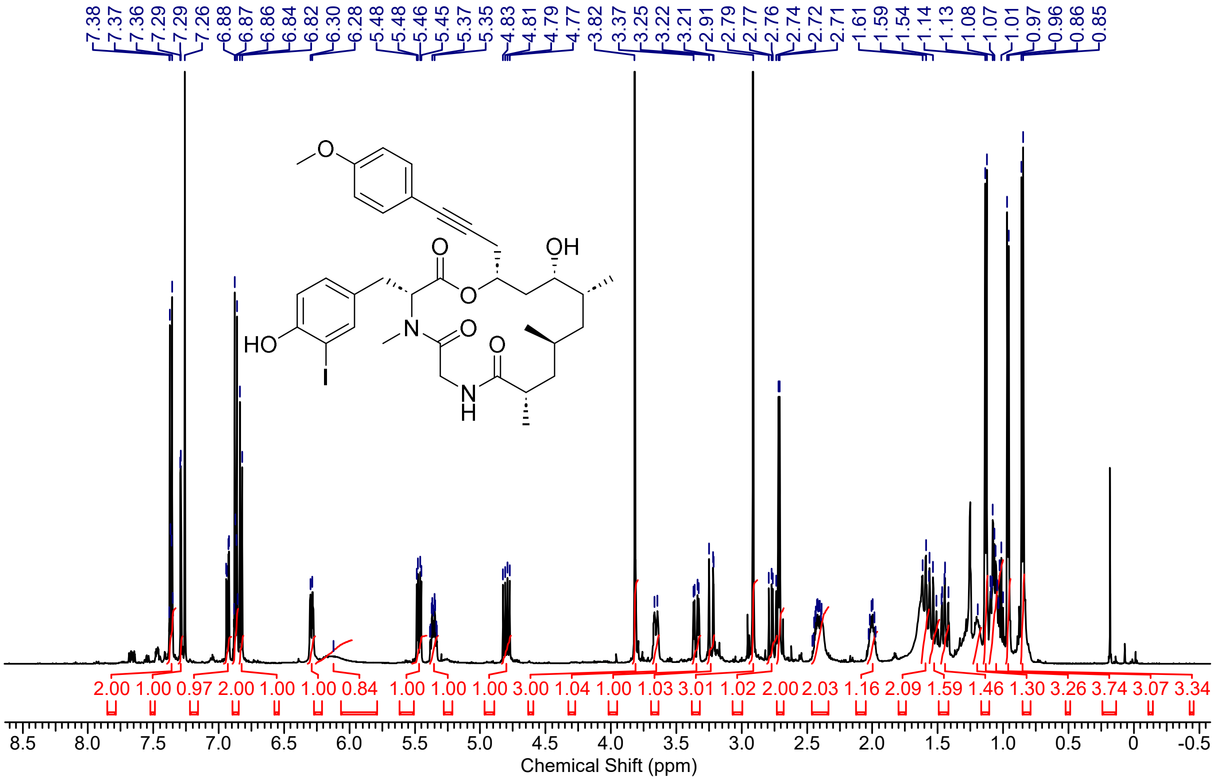

^{13}C-NMR (125 MHz, $CDCl_3$)

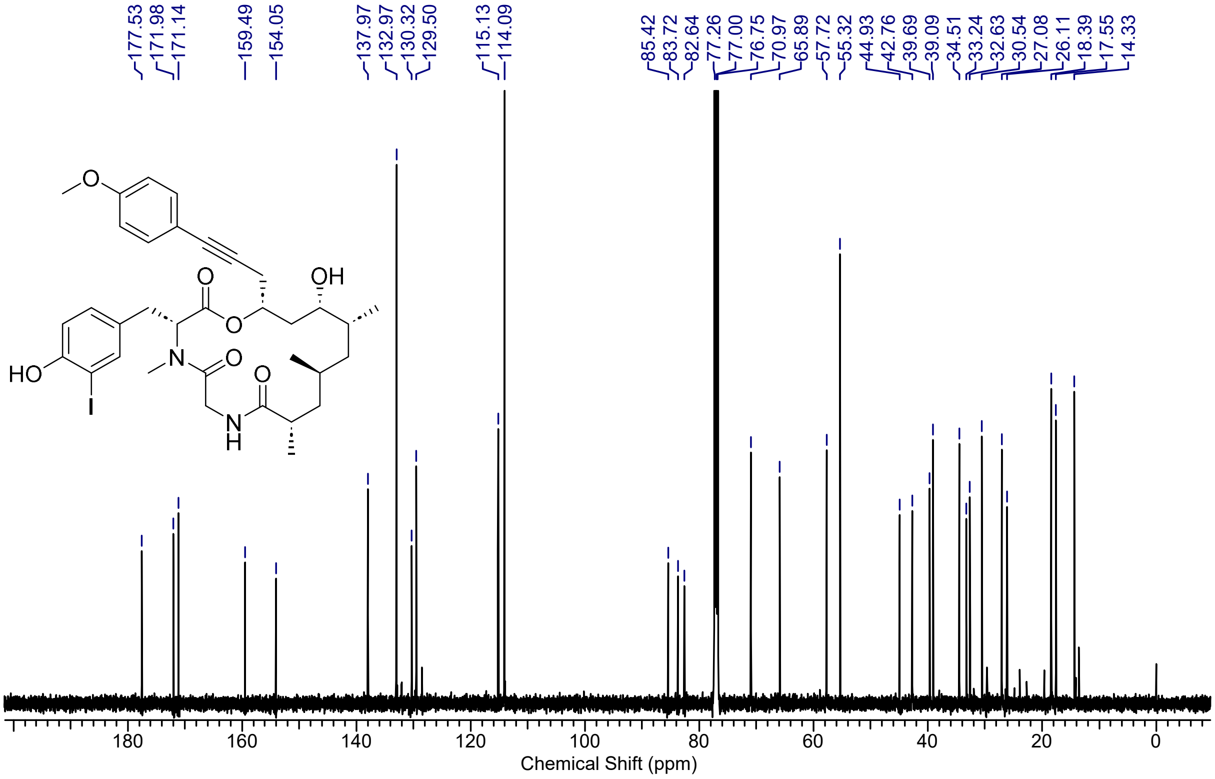

(3*R*,9*S*,11*S*,13*R*,14*S*,16*R*)-14-Hydroxy-3-(4-hydroxy-3-iodobenzyl)-4,9,11,13-tetramethyl-16-[3-(4-nitrophenyl)prop-2-yn-1-yl]-1-oxa-4,7-diazacyclohexadecane-2,5,8-trione (10c)

^{1}H-NMR (500 MHz, $CDCl_3$)

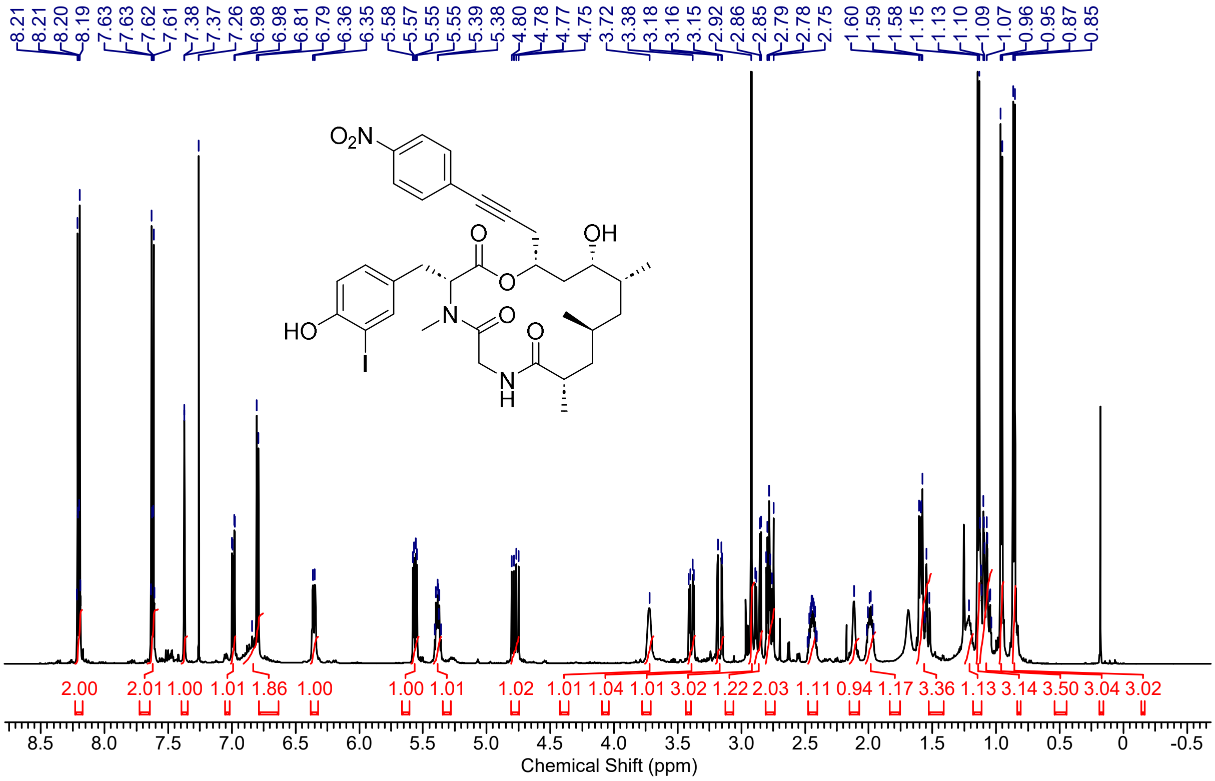

^{13}C-NMR (500 MHz, $CDCl_3$)

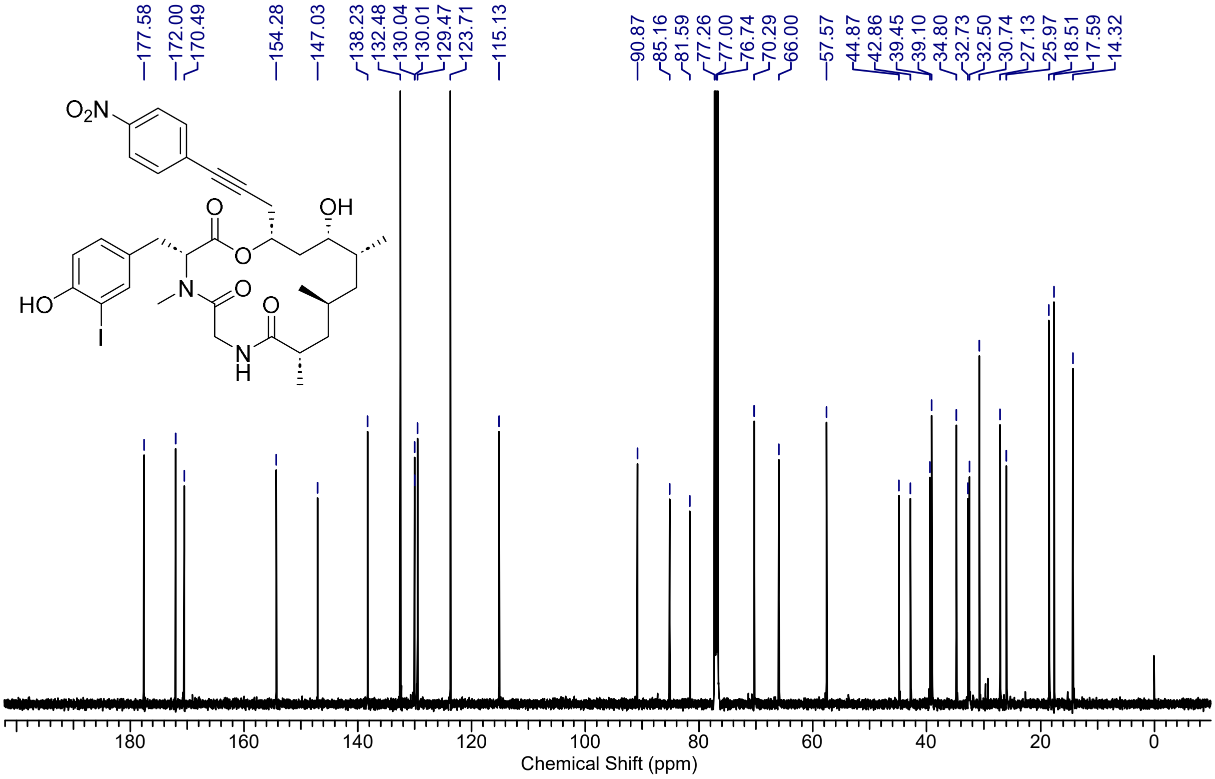

(3*R*,9*S*,11*S*,13*R*,14*S*,16*R*)-14-Hydroxy-3-(4-hydroxy-3-iodobenzyl)-16-{3-[(2-hydroxyethyl)thio]-propyl}-4,9,11,13-tetramethyl-1-oxa-4,7-diazacyclohexadecane-2,5,8-trione (11a)

^{1}H-NMR (500 MHz, $CDCl_3$)

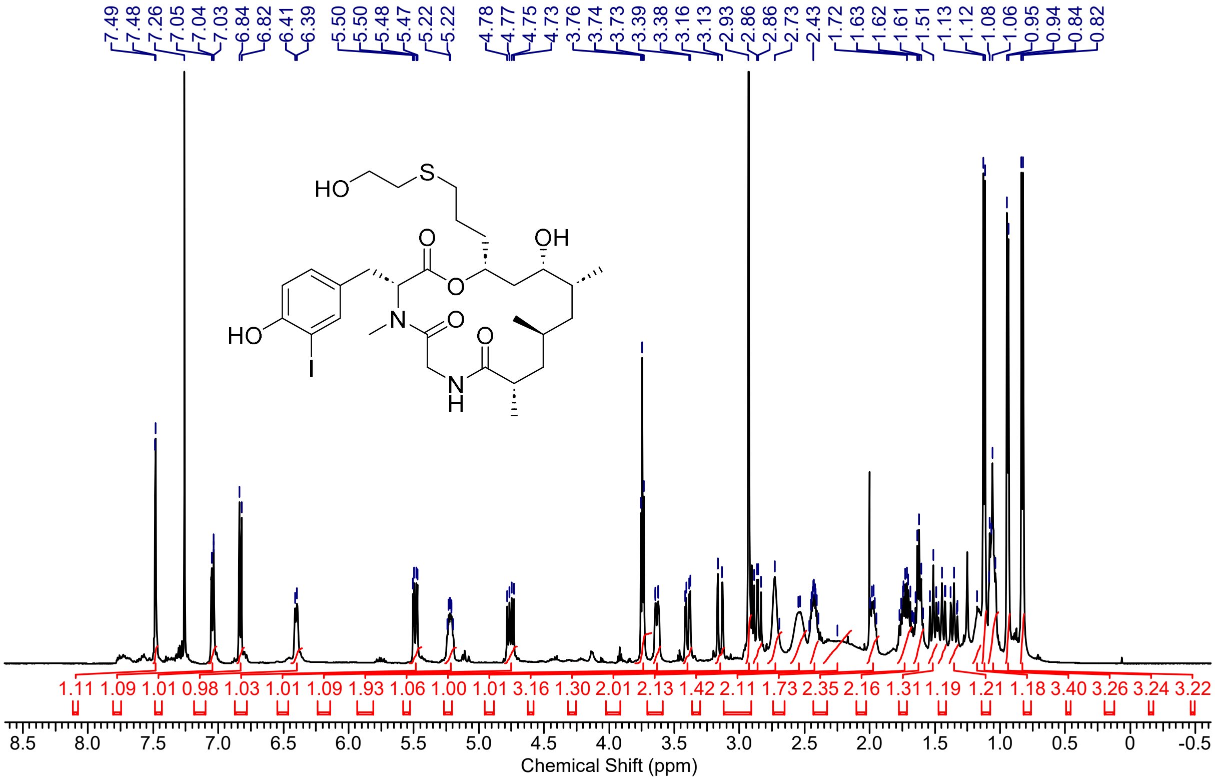

^{13}C-NMR (125 MHz, $CDCl_3$)

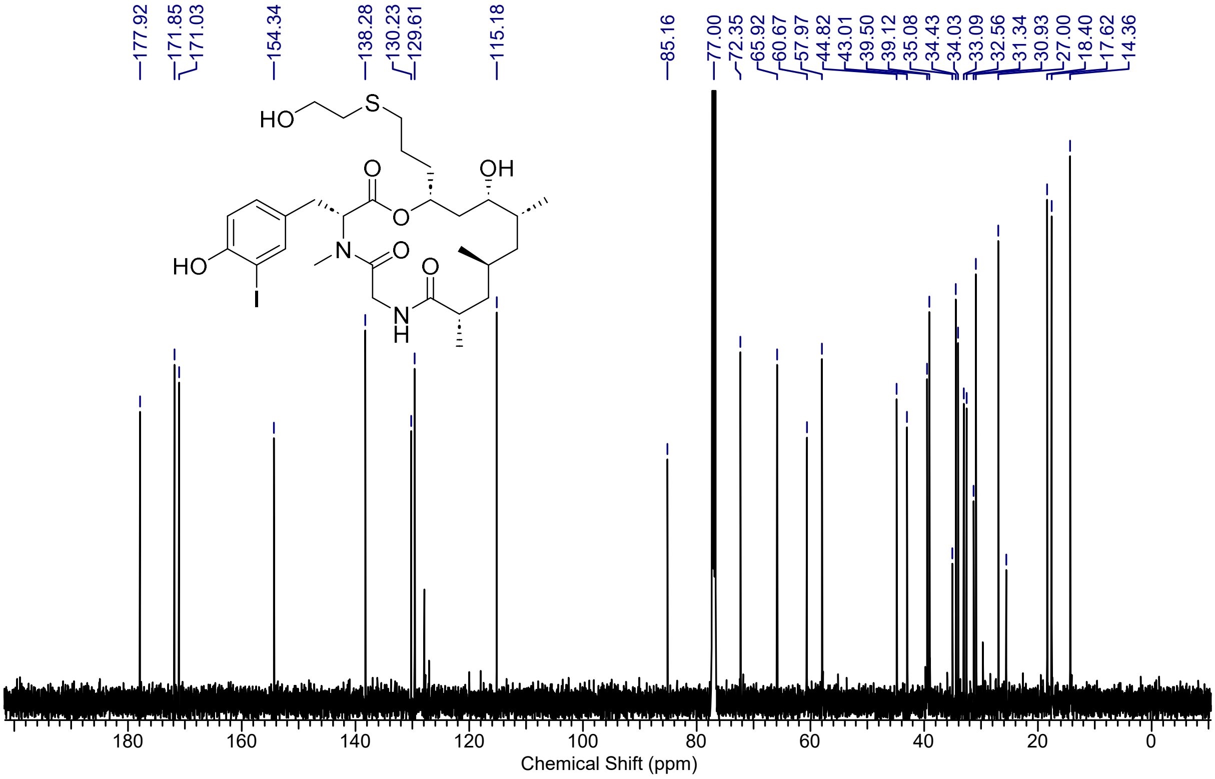

(3*R*,9*S*,11*S*,13*R*,14*S*,16*R*)-14-Hydroxy-3-(4-hydroxy-3-iodobenzyl)-4,9,11,13-tetramethyl-16-[3-(pentylthio)propyl]-1-oxa-4,7-diazacyclohexadecane-2,5,8-trione (11b)

^{1}H-NMR (500 MHz, $CDCl_3$)

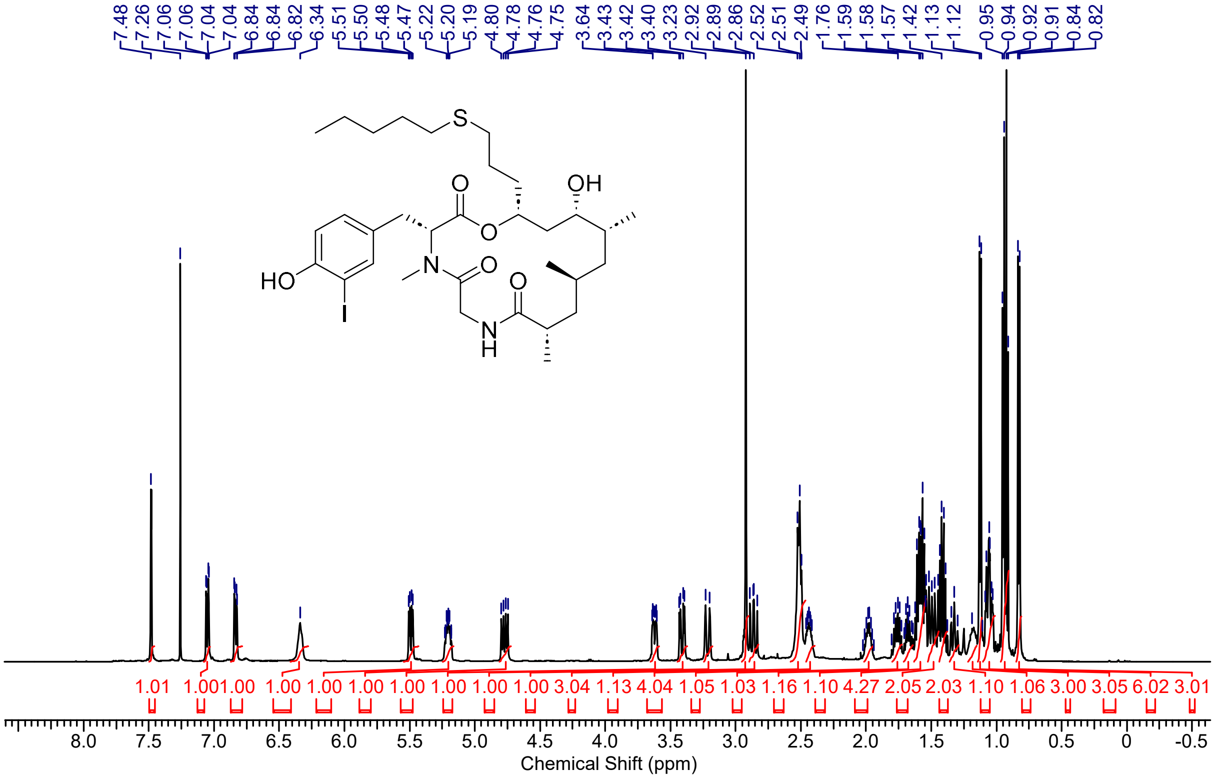

^{13}C-NMR (125 MHz, $CDCl_3$)

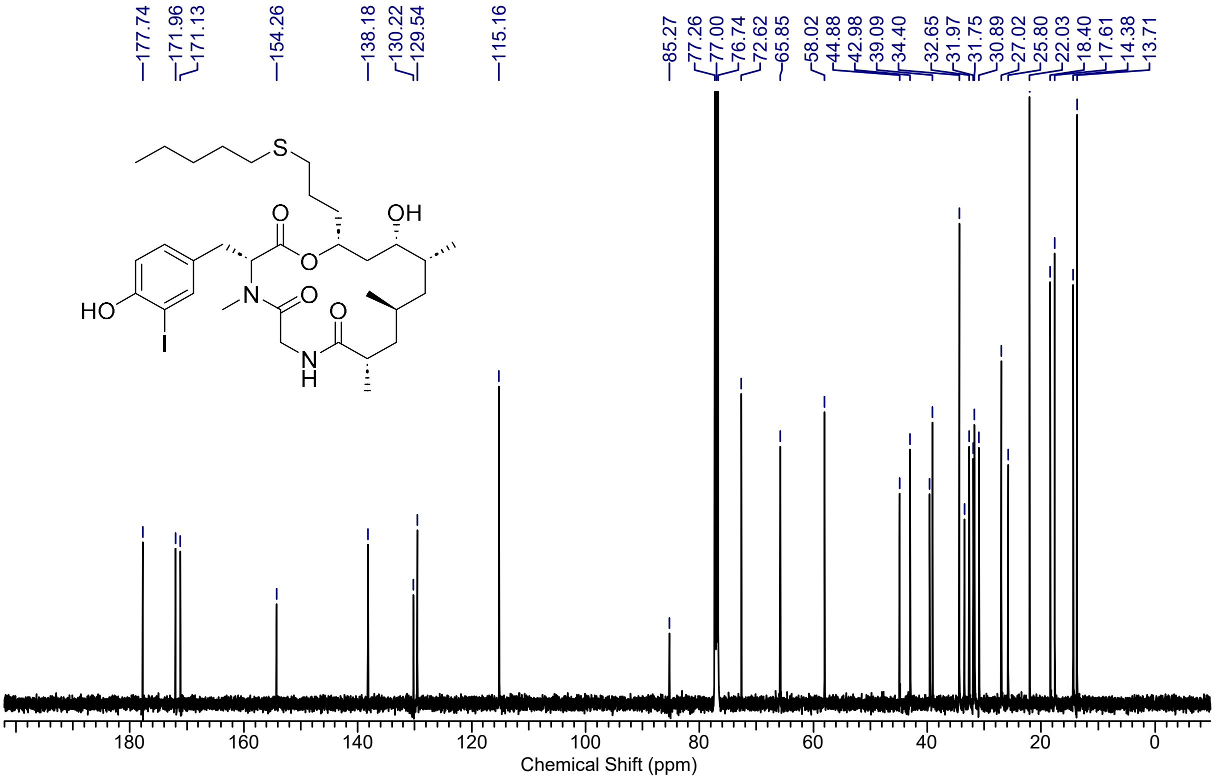

(3*R*,9*S*,11*S*,13*R*,14*S*,16*R*)-16-[3-(Benzylthio)propyl]-14-hydroxy-3-(4-hydroxy-3-iodobenzyl)-4,9,11,13-tetramethyl-1-oxa-4,7-diazacyclohexadecane-2,5,8-trione (11c)

^{1}H-NMR (500 MHz, $CDCl_3$)

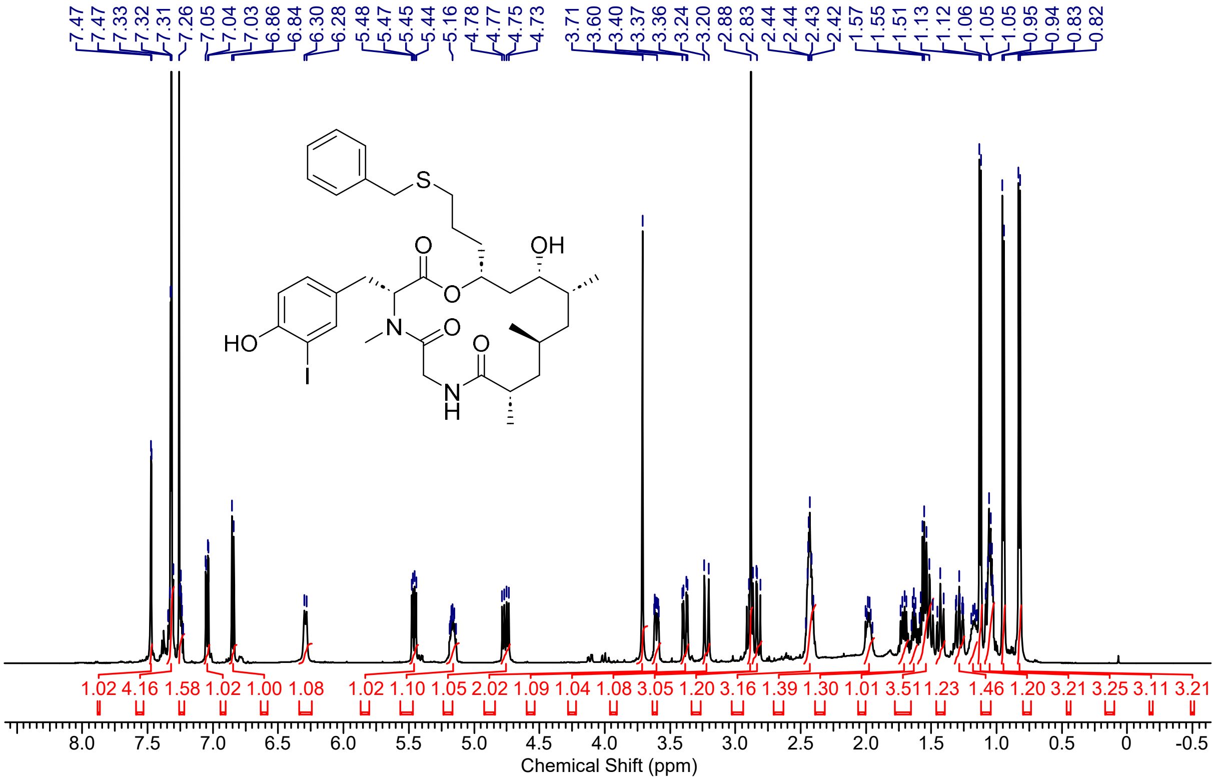

^{13}C-NMR (125 MHz, $CDCl_3$)

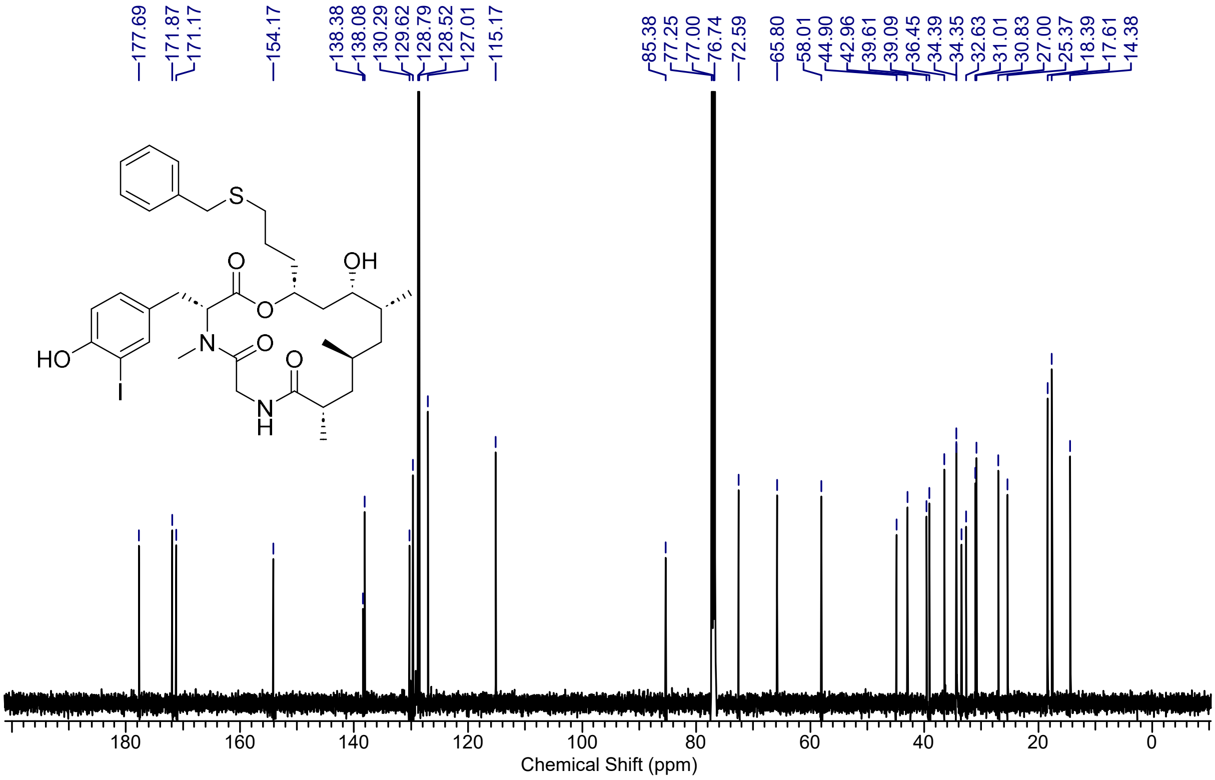

Ethyl 3-({3-[(3*R*,9*S*,11*S*,13*R*,14*S*,16*R*)-14-hydroxy-3-(4-hydroxy-3-iodobenzyl)-4,9,11,13-tetramethyl-2,5,8-trioxo-1-oxa-4,7-diazacyclohexadecan-16-yl]propyl}thio)propanoate (11d)

^{1}H-NMR (500 MHz, $CDCl_3$)

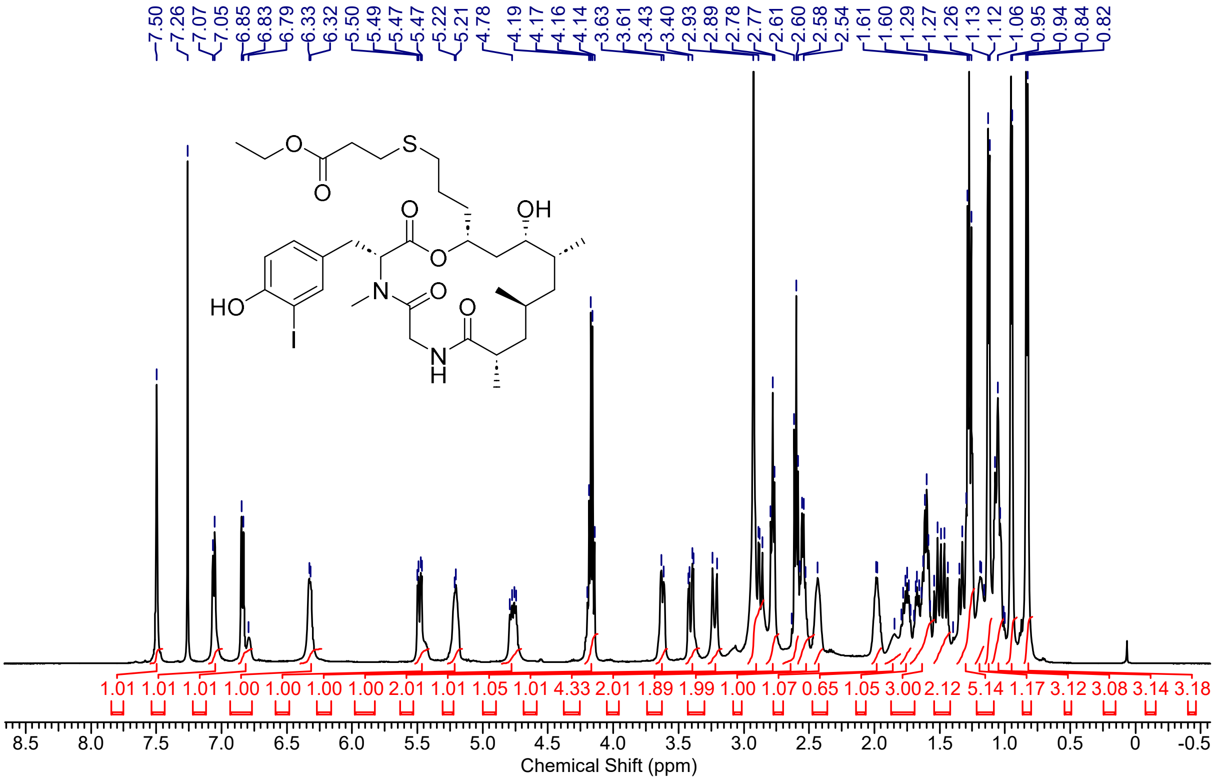

^{13}C-NMR (125 MHz, $CDCl_3$)

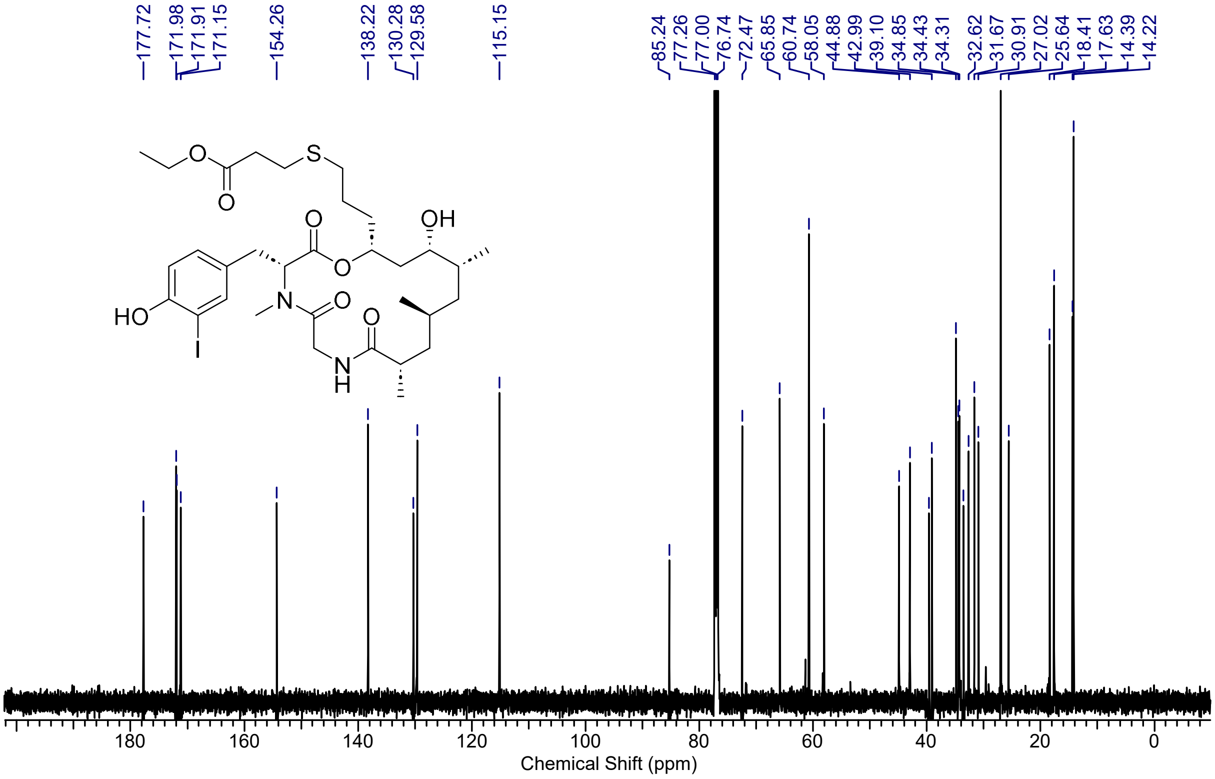

2-({3-[(3*R*,9*S*,11*S*,13*R*,14*S*,16*R*)-14-Hydroxy-3-(4-hydroxy-3-iodobenzyl)-4,9,11,13-tetramethyl-2,5,8-trioxo-1-oxa-4,7-diazacyclohexadecan-16-yl]propyl}thio)acetic acid (11e)

^{1}H-NMR (500 MHz, $CDCl_3$)

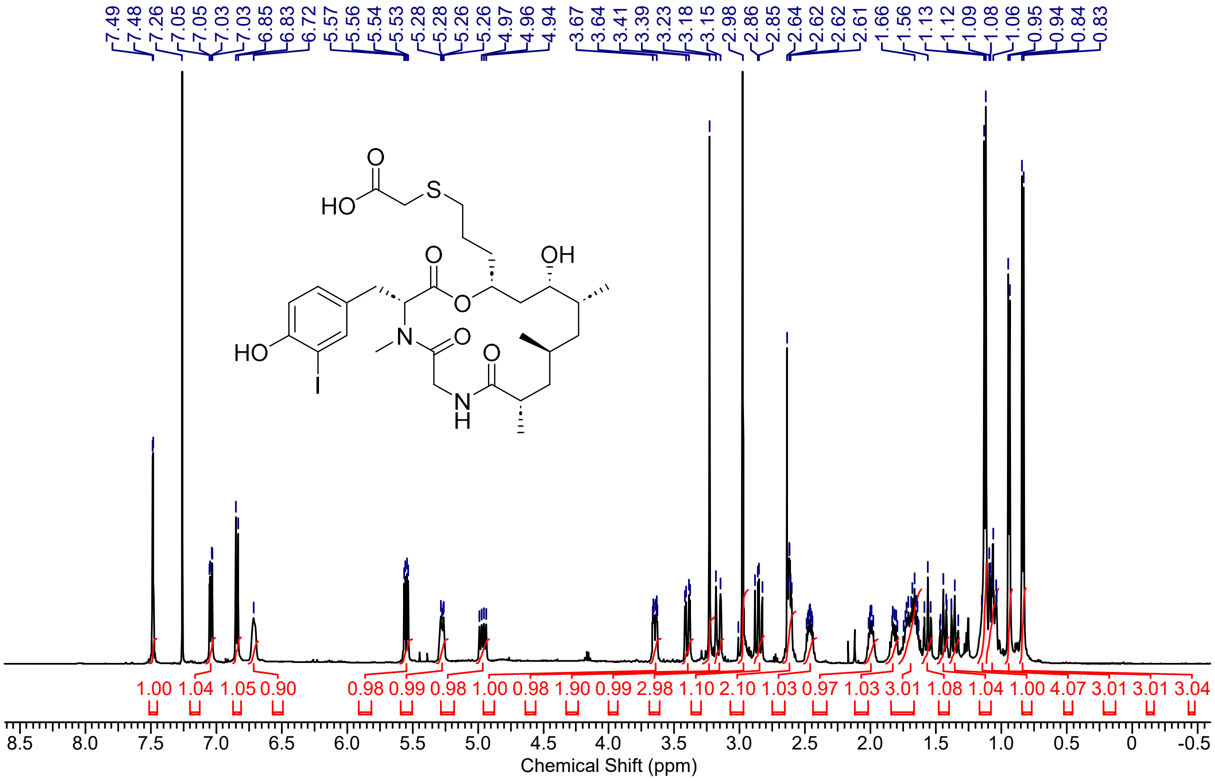

^{13}C-NMR (125 MHz, $CDCl_3$)

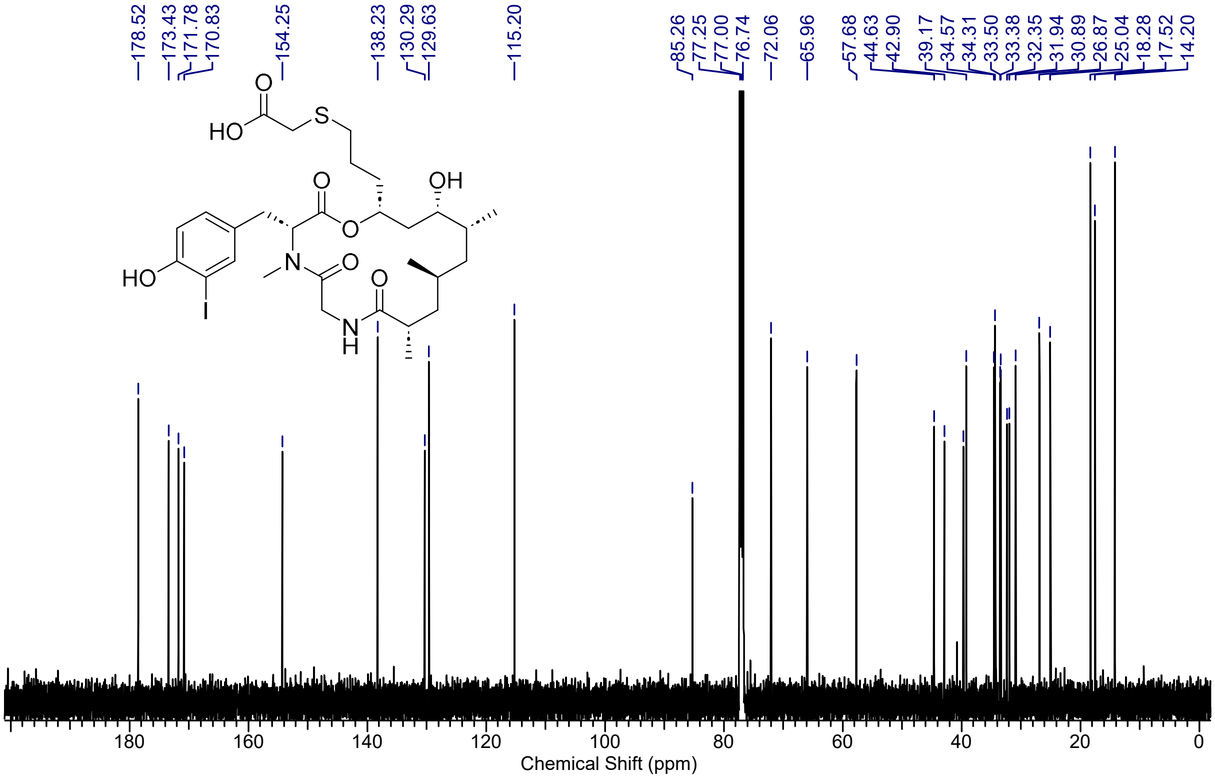

***S*-{3-[(3*R*,9*S*,11*S*,13*R*,14*S*,16*R*)-14-Hydroxy-3-(4-hydroxy-3-iodobenzyl)-4,9,11,13-tetramethyl-2,5,8-trioxo-1-oxa-4,7-diazacyclohexadecan-16-yl]propyl} ethanethioate (11f)**

^{1}H-NMR (500 MHz, $CDCl_3$)

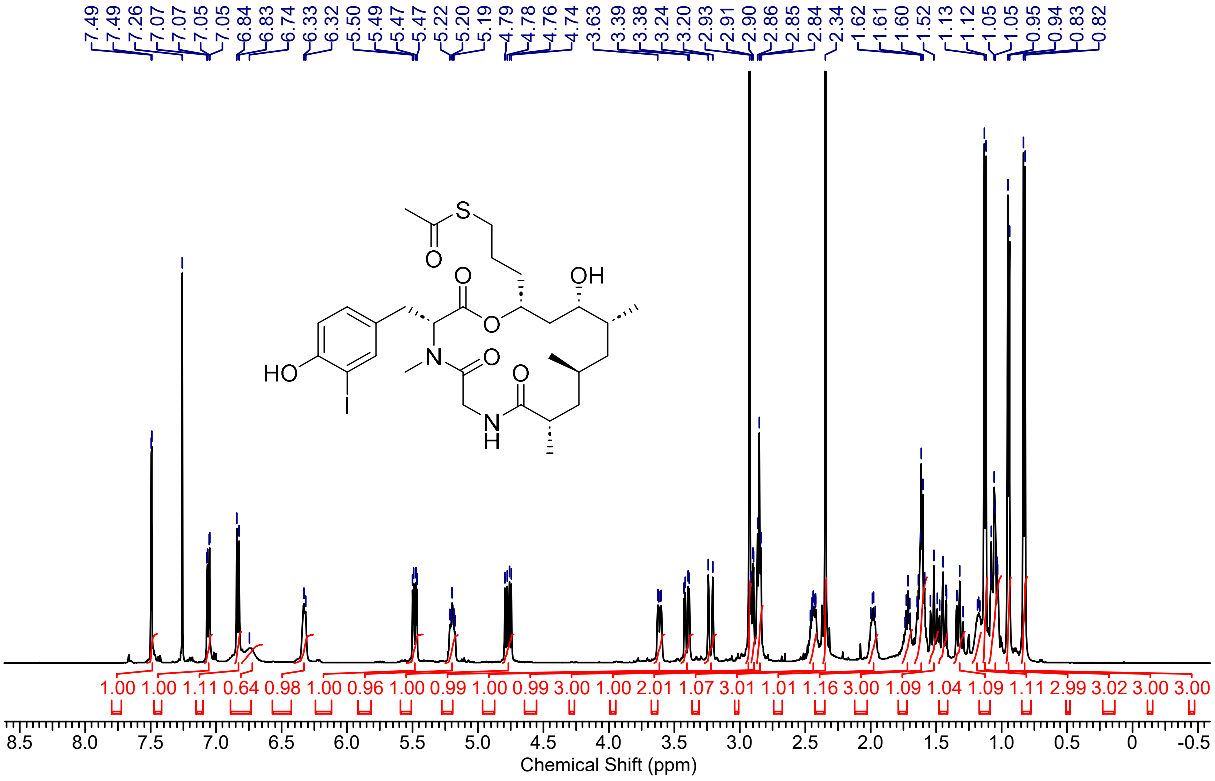

^{13}C-NMR (125 MHz, $CDCl_3$)

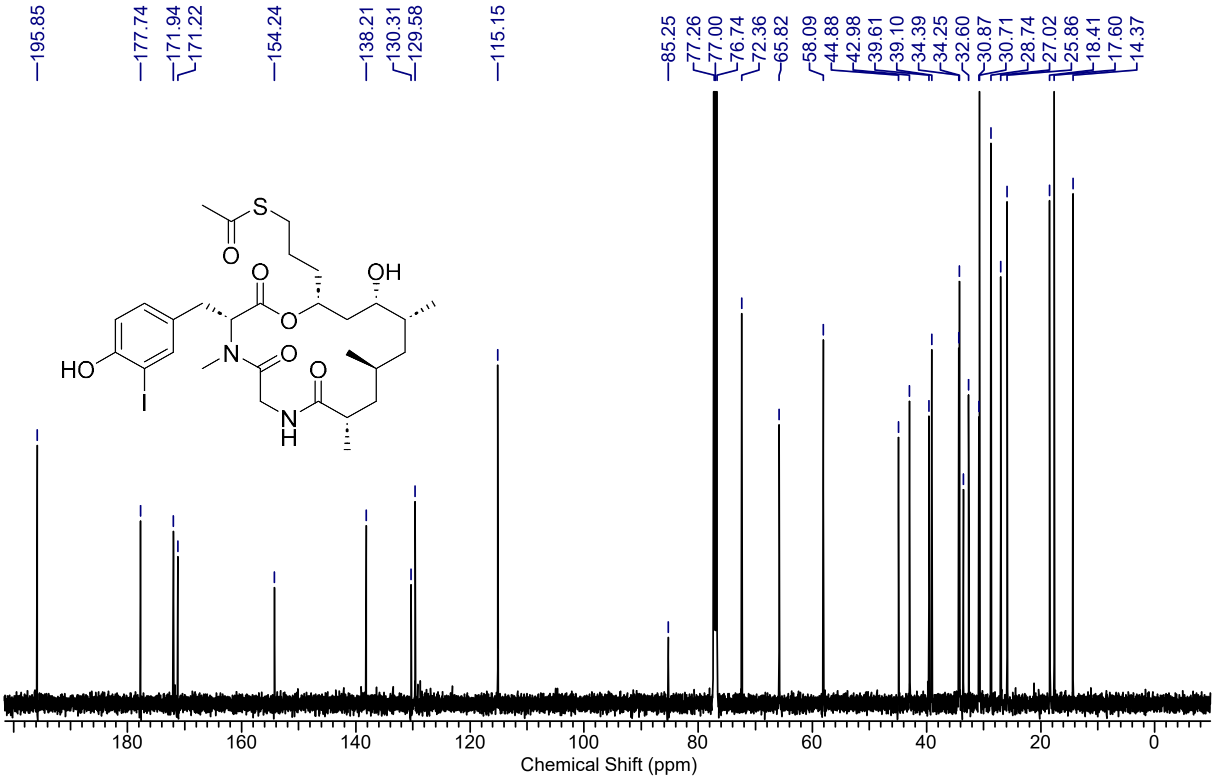

Supplement: Supplementary file 1 [file marinedrugs-22-00165-s001.zip › marinedrugs-2929901-supplementary.pdf]
